# Two way controls of apoptotic regulators consign *DmArgonaute-1* a better clasp on it

**DOI:** 10.1371/journal.pone.0190548

**Published:** 2018-01-31

**Authors:** Tanmoy Mondal, Indira Bag, Pushpavalli SNCVL, Koteswara Rao Garikapati, Utpal Bhadra, Manika Pal Bhadra

**Affiliations:** 1 Department of Chemical Biology, CSIR-Indian Institute of Chemical Technology, Tarnaka, Hyderabad, Telangana State, India; 2 Academy of Scientific and Innovative Research (AcSIR), CSIR-IICT Campus, Hyderabad, India; 3 Gene Silencing and Functional Genomics Group, CSIR-Centre For Cellular and Molecular Biology, Uppal Road, Hyderabad, Telangana State, India; Ecole normale superieure de Lyon, FRANCE

## Abstract

Argonaute family proteins are well conserved among all organisms. Its role in mitotic cell cycle progression and apoptotic cell elimination is poorly understood. Earlier we have established the contribution of *Ago-1* in cell cycle control related to G2/M cyclin in *Drosophila*. Here we have extended our study in understanding the relationship of *Ago-1* in regulating apoptosis during *Drosophila* development. Apoptosis play a critical role in controlling organ shape and size during development of multi cellular organism. Multifarious regulatory pathways control apoptosis during development among which highly conserved JNK (c-Jun N-terminal kinase) pathway play a crucial role. Here we have over expressed *Ago-1* in *Drosophila* eye and brain by employing UAS (upstream activation sequence)-GAL4 system under the expression of eye and brain specific driver. Over expression of *Ago-1* resulted in reduced number of ommatidia in the eye and produced smaller size brain in adult and larval *Drosophila*. A drastic reversal of the phenotype towards normal was observed upon introduction of a single copy of the dominant negative mutation of *basket* (*bsk*, *Drosophila* homolog of JNK) indicating an active and physical involvement of the *bsk* with *Ago-1* in inducing developmental apoptotic process. Further study showed that *Ago-1* stimulates phosphorylation of JNK through *transforming growth factor-β activated kinase 1*- *hemipterous (Tak1-hep)* axis of JNK pathway. JNK phosphorylation results in up regulation of pro-apoptotic genes *head involution defective (hid)*, *grim* & *reaper (rpr)* and induces activation of *Drosophila* caspases (cysteinyl aspartate proteinases);DRONC (Death regulator Nedd2-like caspase), ICE (alternatively Drice, Death related ICE-like caspase) and DCP1 (Death caspase-1) by inhibiting apoptotic inhibitor protein DIAP1 (Death-associated inhibitor of apoptosis 1). Further, *Ago-1* also inhibits miR-14 expression to trigger apoptosis. Our findings propose that *Ago-1* acts as a key regulator in controlling cell death, tumor regression and stress response in metazoan providing a constructive bridge between RNAi machinery and cell death.

## Introduction

Apoptosis is collection of multiple perturbations of the cellular architecture and play an important role in tissue homeostasis and pattern formation of an organism. This process allows apoptotic cells to be dismantled in a manner that minimizes damage and disruption of adjacent cells [1]. These events are orchestrated by a series of cysteine proteases family members referred as caspases. Developmental apoptotic death response of cells is mediated by JNK (c-Jun N-terminal kinase), a member of the MAPK (mitogen-activated protein kinase) family [2]. JNK activity is under tight regulation of reversible phosphorylation process stimulated by protein kinases wherein JNK–specific phosphatases are responsible for dephosphorylation and inactivation of JNK. Activation of JNK pathway releases cytochrome-c from mitochondria and activates caspase cascade to induce apoptosis [3]. Abolition of JNK signaling causes a series of defects in response to stress induced apoptotic response during developmental processes, enabling the tumor cells to bypass apoptotic cell death and achieve metastatic activity [4]. JNK1 and JNK2 are essential for suppressing the oncogenic changes and tumorigenesis. Loss of function of JNK3 is associated with development of brain tumors [5]. An upstream JNK kinase, encoded by *mkk4* gene has been identified as suppressor of tumorigenesis and metastasis [6]. In *Drosophila*, TNF ortholog *egr (eiger*) induces cell death through dTAK1 (transforming growth factor-β activated kinase 1, Fly JNK kinase kinase), *hep (hemipterous*, the *Drosophila* JNK kinase) and *Drosophila* JNK *bsk (basket*) [7–10]. Other *bsk*-mediated signaling pathways that modulate developmental apoptosis have not been elucidated fully.

AGO (Argonaute) family proteins are highly conserved across both animal and plant kingdom. In mammals both AGO and PIWI (P-element Induced WImpy testis) includes four members in their subfamily [11]. Structurally AGO proteins contain four domains- an amino terminal domain, a highly conserved mid domain, PAZ (Piwi/Argonaute/Zwille) domain & PIWI domain. AGO proteins are direct binding partners of existing small RNA. Both AGO1 and 2 are well studied, because of its role in miRNA (microRNA) biogenesis and as a major representative of miRNA effector complex (RISC complex). By interacting with AGO proteins miRNA guide the RISC (RNA-induced silencing complex) to its mRNA (messenger RNA) targets to induce translational repression or degradation. As a post-transcriptional regulator for a large number of vital genes, miRNAs play crucial role in controlling a wide range of cellular processes and their mis expression lead to development of various diseases [12]. miRNAs can act as a tumor inducer or inhibitor by altering the expression of tumor suppressor genes and oncogenes. Altered expression of miRNA biogenesis associated proteins such as DROSHA and DICER is frequently observed during tumorigenesis and cancer progression [13]. *Drosophila Ago-1* is broadly expressed in the embryo; central nervous system and imaginal discs of larvae. Maternal and zygotic *Ago-1* mutant embryos show different developmental defects. Earlier studies have also shown that *Drosophila* AGO1 is localized to the cytoplasmic region of the cell [14]. In our earlier study we have shown the regulatory role of *Ago-1* in association with the G2/M cyclin in controlling cell cycle during development. We also, identified the involvement of two embryonic miRs (miRNAs)- miR-981 and miR-317 as spatiotemporal regulator of cyclin B [15]. However, till date, little is known about the role of *Ago* on apoptotic process.

In this study, we report a connection between AGO1 and the JNK signaling pathway that opens a crosstalk with different signaling pathways and gene silencing mechanism. We demonstrate that subtle changes in the expression of *Ago-1* lead to chronic effect on gene regulation that promotes different diseases including cancer. Our results demonstrate that AGO1 activates JNK signaling pathway through JNK phosphorylation *in vivo* and mediate *Ago-1* induced apoptosis in developing fly organs and thereby control organ size and structure. Apart from that, *Ago-1* also negatively regulates the expression of apoptotic inhibitor micro RNA, miR-14. Regulation of JNK pathway in one hand and miR-14 expression in other hand allows *Ago-1* to take a better control with more perfection on developmental apoptosis. This understanding of the new role of *Ago-1* unravels the pathway dictating the principle and cause for various diseases including cancer and neuro degenerative diseases.

## Materials and methods

### Fly stocks and genetic crosses

All flies were obtained from Bloomington *Drosophila* Stock Centre and maintained at standard condition, at 24°±1°C temperature and reared on yeast-agar standard medium. All crosses were carried out at 18°C (except *GMR GAL4* and CyO marker related crosses). SEM (Scanning Electron Microscope) images of newly immerged flies were taken after maintaining them at 24°C for 3 days. All details of genetic crosses are mentioned in S2 File.

### Microarray: Labeling and hybridization

The miRNA labeling was performed using miRNA Complete Labeling and Hyb Kit (Agilent Technologies, Part Number: 5190–0456). About 200ng of total RNA was hybridized on the miRNA 8x60K Arrays. The hybridization was carried out at 55°C for 20 hours followed by washes using Gene Expression Wash Buffer1 (Agilent Technologies, Part Number 5188–5325) at room temperature for 5 minutes and Gene Expression Wash Buffer 2 (Agilent Technologies, Part Number 5188–5326) at 37°C for 5 minutes. Slide was scanned on a G2600D scanner (Agilent Technologies).

### Chromatin immunoprecipitation (ChIP)

Chromatin immunoprecipitation (ChIP) assay was performed after isolating chromatin from ~10,000 *UAS Ago-1/GMR GAL4* adult fly heads followed by crosslinking DNA-protein with formaldehyde. Target amplification was carried out using region-specific primer sets (Table-B in S1 File).

### Immuno Precipitation

Immuno Precipitation (IP) was carried out using RNA pol II antibody (detailed method has mentioned in supplemental section). The eluted proteins were run on SDS-PAGE along with 5% input sample and western blot was carried out using RNA pol II (Abcam# ab817) and AGO1 (Abcam# ab5070)antibody.

### Statistical analysis

Mean values from three independent experiments are presented with error bars corresponding to ±S.D. or ±S.E.M. as indicated. Student's t-test was performed between control and tested groups. Statistical analysis was performed using Microsoft Excel 2013 and Prism Graphpad. Significance is indicated as ***P<0.001,**P<0.01 and *P<0.05.

## Results and discussion

### Results

#### Over expression of *Ago-1* leads to increased apoptosis in *Drosophila*

**Fig 1:**
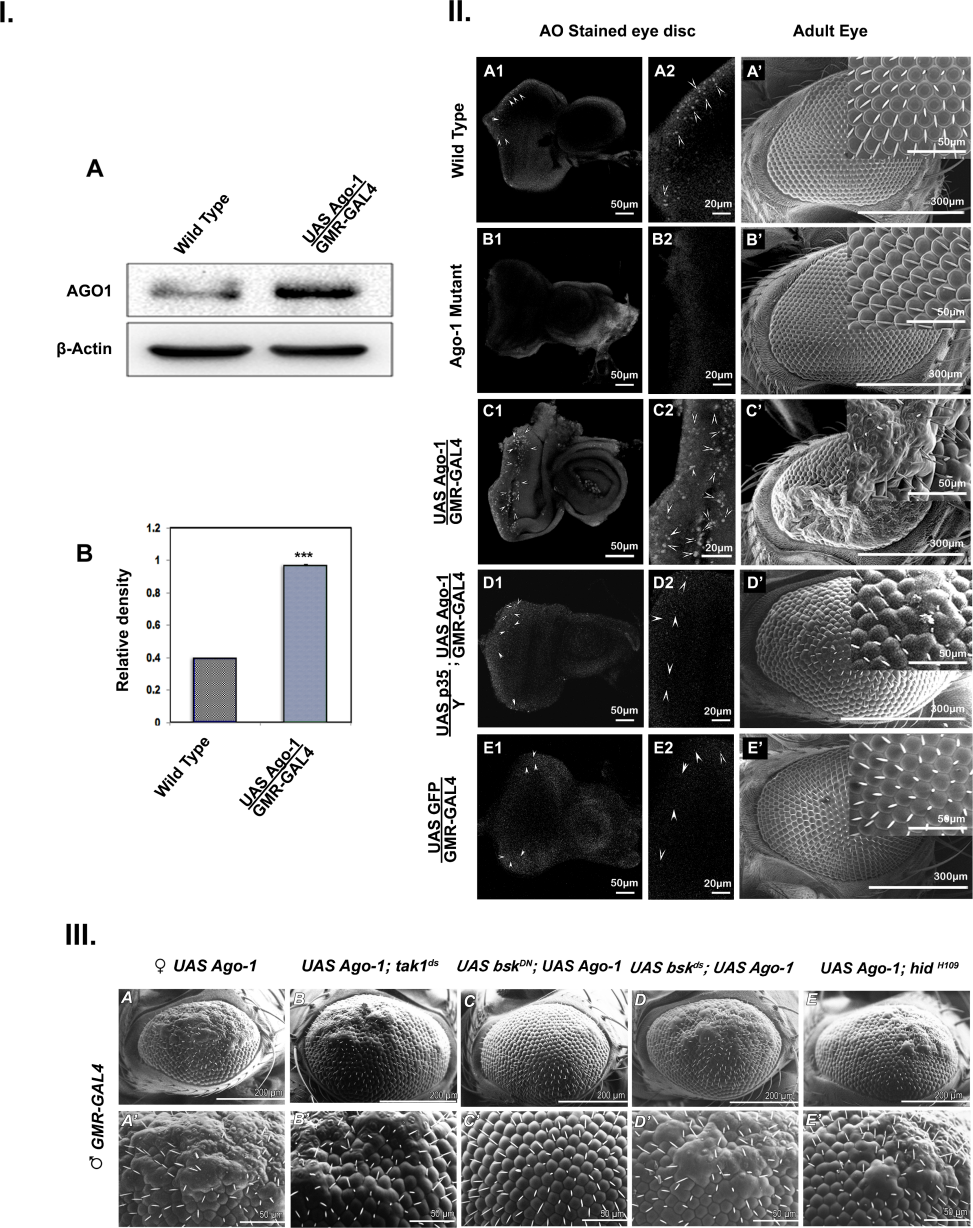
*Ago-1* over expression induces apoptosis in *Drosophila* eye. **I.** (**A)** Western blot analysis showing the expression of proteins isolated from adult fly heads of wild type and *Ago-1* over expressed stocks. (**B)** Graphical presentation of AGO1 band intensity relative to β-Actin loading control. The average from three independent experiments was taken and plotted. **II.** (**A1-A2, B1-B2, C1-C2)** Acridine Orange (AO) staining showing more apoptotic population (marked by arrow head)in *Ago-1* over expressed 3^rd^ instar larval eye imaginal disc compared to wild type and down regulated group. (**D1 and D2**) AO positive cells reduced significantly in *Ago-1* over expressed eye disc upon introduction of baculovirus p35 trans-gene in fly eye. (**E1 and E2**) Over expression of transgenic control protein (GFP) in developing eye can’t increase AO positive cells in developing fly eye. (**A’, B’, C’)** Scanning electron micrograph of adult eyes showing reduced number of normal ommatidia in *Ago-1* over expressed line. (**D’**) baculovirus p35 protein can inhibit *Ago-1* over expression induced apoptotic phenotype in fly eye (**E’**) GFP over expression can’t induce *Ago-1* over expression like eye phenotype (**III.)** *Drosophila* JNK pathway is triggered by *Ago-1* over expression. **(A)** Adult eye phenotype of *Ago-1* over expressed fly under Scanning Electron Microscope (SEM). **(A’)** Ommatidia structure of same fly. **(III. B.)** Adult eye phenotype of *tak1* deficient mutant, **(B’)** same in higher magnification. **(C)** Adult eye phenotype of dominant negative mutation of *basket*, **(C’)** magnified view of same showing complete recovery of ommatidia morphology. **(D)** Adult eye phenotype of *bsk* deficient with *Ago-1* over expression showing partial recovery **(D’)** ommatidia morphology. **(E)** Mutation in *hid* gene (*hid*^*H109*^) prevents *Ago-1* over expression induced apoptotic phenotype in adult fly eye as well as **(E’)** Ommatidia structure of the same.

In our earlier study we had identified the role of *Ago-1* in cell cycle control via the regulation of cyclins. We therefore wanted to see whether it plays any role in apoptosis, as programmed cell death during cell cycle is extremely essential in maintaining a check for proper development. We therefore generated *Drosophila* lines that ectopically overexpress *Ago-1* gene in different organs of *Drosophila*. *UAS Ago-1* flies were crossed with *GMR-GAL4* driver carrying flies, thereby ectopically expressing *Ago-1* in the developing eye. Ectopic expression of *Ago-1* at both transcriptional and translational level was confirmed by performing quantitative real time PCR and western blot analysis. Results showed an increase in the intensity of expression of both *Ago-1* transcripts and proteins (S1 Fig and Fig 1IA and 1IB). Interestingly, ectopic overexpression of *Ago-1* in the adult eyes lead to phenotypic changes that resembled apoptotic cell death compared to the control flies that contained endogenous copy of *Ago-1*. Observation of adult eyes under SEM (Scanning Electron Microscopy) showed reduced and deformed omatidia in *Ago-1* overexpressed flies compared to control flies (Fig 1IIA’–1IIC’). Number of fully formed omatidia from each individual eye was counted and average number from twenty individual flies presented as a graphical format (S2 Fig). To understand whether apoptotic cell death is responsible, we performed AO (Acridine Orange) staining in eye imaginal discs of third instar larvae arising from stocks that contained overexpressed *Ago-1* (*UAS Ago-1/GMR GAL4*) as well as control stocks (+/GMR GAL4) and *Ago-1* loss of function allele mutant fly stock (*P*{*hs-Ago-1*}; *Ago-1*^*k08121*^/ *Ago-1*^*k08121*^). Due to absence of *Ago-1* functionality, homozygous mutants arising from this stock do not complete their embryonic development [14, 16, 17], resulting in embryonic lethality. However, these homozygous mutants are capable of surviving to adulthood with the insertion of transgene *P*{*hs-Ago-1*} along with homozygous mutant genotype and upon receiving daily heat shock treatment consequently that supplies the amount of AGO1 needed for completion of its embryonic development. To revive a minimum number of survivals till the larval stage for this study we performed a series of heat shock experiments by subjecting embryos to heat shock for different time periods. Proteins were isolated from emerging larvae for performing western blot analysis. Result showed that a minimum expression of AGO1 was seen when the embryos were subjected to one hour heat shock per day to achieve the basal level of AGO1 for completion of embryonic development and reach to larval stage. Acridine Orange staining (AO) in the eye imaginal discs of the third instar larvae showed a large number of positively stained apoptotic cells in the discs arising from larvae in which *Ago-1* is overexpressed (*UAS Ago-1/GMR GAL4*) compared to discs isolated from wild type stock as well as discs isolated from stock in which *Ago-1* is down regulated (Fig 1IIA1–1IIC2) confirming that apoptosis was indeed induced due to over expression of *Ago-1* (S2 Fig). For further confirmation of apoptosis, we co-expressed p35 protein, a well known inhibitor of apoptosis in *Ago-1* over expressed eye disc. A clear reduction of Ago-1 induced apoptosis was observed clearly indicating that over expression of Ago-1 indeed lead to induction of apoptosis in developing fly eye (Fig 1IID1, 1IID2 and 1IID’). To check whether the observed apoptosis upon over expression of *Ago-1* is clearly due to its effect and not related to any stress response, we over expressed Green Fluorescence Protein (GFP) in developing fly eye. Interestingly, no significant apoptotic cell death was observed indicating clearly that the observed apoptotic cell death in the fly eye was due to the over expression of *Ago-1* (Fig 1IIE1, 1IIE2 and 1IIE’). We also performed a similar experiment to see the generalized effect of inducing apoptosis by over expression of *Ago-1* using *ptc GAL4* that shows its expression in the developing wing discs as well as in the adult wings. AO staining was performed in the wing discs of 3^rd^ instar larvae. Similar positively stained cells were seen in the discs arising from *Ago-1* overexpressed stocks compared to controls. Moreover, over expression of *Ago-1* produced by *ptc GAL4* also resulted in the loss of anterior cross vein and led to narrowing of the space between longitudinal vein3 and longitudinal vein4 in adult fly wings demonstrating induction of apoptosis (S3 Fig).

#### Over expression of *Ago-1* stimulates *Drosophila* JNK pathway

Argonaute family proteins play an important role in RNAi pathway. As overexpression of *Ago-1* resulted in increased cell death, we wanted to see its effect on the players of cell death inducers NF-κB and JNK pathway proteins. JNK cascade comprises of several members among whom JNKs are MAPK superfamily signal-transduction protein involved in apoptotic signaling [2, 18]. Also MAPKKK superfamily protein TAK1 known as D-tak, DmTak1 in *Drosophila* is an important component of JNK signaling pathway and play a critical role in JNK induced cell death in the development of fly [19]. Flies were generated that carried a copy of *Tak1* (*Tak1*^*ds*^) along with over expressed *Ago-1*(*Tak1*^*ds*^*;UAS Ago-1/GMR GAL4*). Number of omatidia from adult eyes of these heterozygous mutant flies was compared to the control and results indicated a significant level of recovery towards normal (Fig 1IIIA, 1IIIA’, 1IIIB and 1IIIB’). Quantitative real time PCR analysis of *Tak1* expression under same genetic background showed an increase in transcript level (S1 Fig) in the eye of adult flies confirming that indeed *Tak1* interacted with *Ago-1* in recovering the eye phenotype. *Drosophila* homolog of mammalian JNK also known as DJNK is encoded by *bsk* [20] and acts downstream of *Tak1[19]. bsk* mutants lead to defects in dorsal closure [21]. It is a stress activated protein kinase involved in upstream activator functions required for morphogenetic movements in embryos and in maintaining cell and tissue polarity in adults [20]. JNK-mediated apoptosis triggers apoptosis in intrinsic tumor suppression process, wherein activation of JNK functions as a cell editor by removing epithelial aberrant cells [22]. As *bsk* is a major component of the JNK signaling cascade in *Drosophila*, we generated flies carrying one copy of over expressed *Ago-1* along with one copy of mutant *bsk* gene (*UAS Ago-1/GMR GAL4; UAS bsk*^*ds*^*/+*). We generated two heterozygous stocks; one carrying single copy of *Ago-1* over expression under the GMR driven GAL4 driver along with one copy of P element inserted RNAi construct against *bsk* (*UAS Ago-1/GMR GAL4; UAS bsk*^*ds*^*/+*), and another one containing same single copy of *Ago-1* over expression along with a dominant negative form of *bsk* allele (*UAS bsk*^*DN*^*; UAS Ago-1/GMR GAL4*). Adult fly eyes visualized under SEM showed an significant increase in the number of omatidia with the introduction of one copy of RNAi mutant of *bsk* (*bsk*^*ds*^) (Fig 1IIID and 1IIID’) in comparison to control (Fig 1IIIA and 1IIIA’), whereas introduction of one copy of the dominant negative form of *bsk* (*bsk*^*DN*^) resulted in complete rescue of eye phenotype (Fig 1IIIC and 1IIIC’).

*Hid*, a member of the *Drosophila* pro-apoptotic protein family is a known inducer of apoptosis [23–25]. *Drosophila* HID is a member of the RHG (REAPER, HID and GRIM) group of proteins that act by binding to the IAPs (Inhibitor of Apoptosis Proteins). We combined a loss of function mutation of *hid (hid*^*H109*^) with the stock having an over expression of *Ago-1* (*UAS Ago-1/GMR GAL4; hid*^*H109*^*/+*). Introduction of a single copy of loss of function mutation of *hid* was capable of reverting the eye phenotype towards normal with a strong increase in the number of ommatidia (Fig 1IIIF and 1IIIF’).

#### *Ago-1* induces apoptosis by regulating JNK phosphorylation

JNKs are members of MAP kinase superfamily that regulate cell proliferation, differentiation and apoptosis [26, 27]. JNK functions downstream of the TNF homologue *egr (eiger)* and its receptor *Wengen* via a conserved kinase signaling cascade that includes fly JNK kinase kinase *Tak1*, JNK kinase, *hep* and *Drosophila* Jun kinase *bsk*. Following the same route, downstream to JNK, the core apoptotic machinery that includes *Hid* and caspases get activated to cause an apoptotic response.

**Fig 2:**
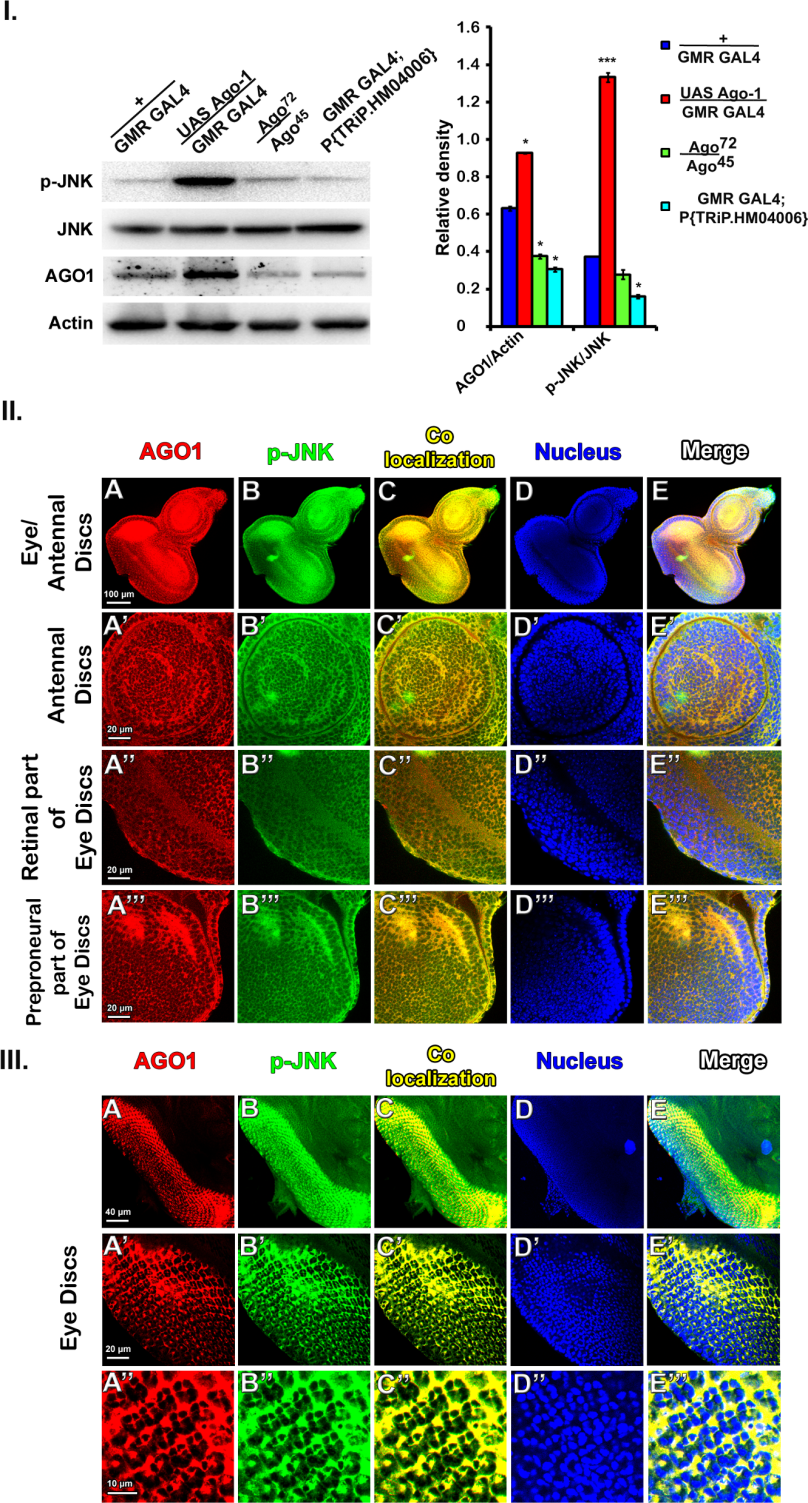
*Ago-1* over expression results in elevated levels of JNK phosphorylation. **I.** Western blot analysis showing the increased level of JNK phosphorylation in *Ago-1* over expressed (*UAS Ago-1/GMR GAL4*) and reduced level in mutant background (*Ago-1*^*72*^*/Ago-1*^*45*^ and *GMR GAL4*; *TRiP*.*HM04006*). Histogram presents mean relative band density (±SEM). **II. (A)** *Ago-1* over expression in 3^rd^ instar larval eye/antennal disc by imaginal discs specific GAL4 [*P{w[+mW*.*hs] = GawB}32B*]. **(A’)** *Ago-1* over expression in the antennal part of the same disc, **(A”)** Retinal part, **(A”‘)** Preproneural part. **(B-B”‘)** JNK phosphorylated areas of the disc. **(C-C”‘)** *Ago-1* over expression co localizes with JNK phosphorylated area of the disc. **(D-D”‘)** DAPI stained part of the same disc. **(E-E”‘)** Merged figure. **III.** *Ago-1* over expression in 3^rd^ instar larval eye disc by eye discs specific GAL4 (*GMR GAL4*). **(A-A”)** *Ago-1* over expression in the eye disc, **(B-B”)** JNK phosphorylated areas of the disc. **(C-C”)** *Ago-1* over expression co localizes with JNK phosphorylated area of the disc. **(D-D”‘)** DAPI staining indicates nuclear region of eye disc cells **(E-E”)** Merged image.

**Fig 3:**
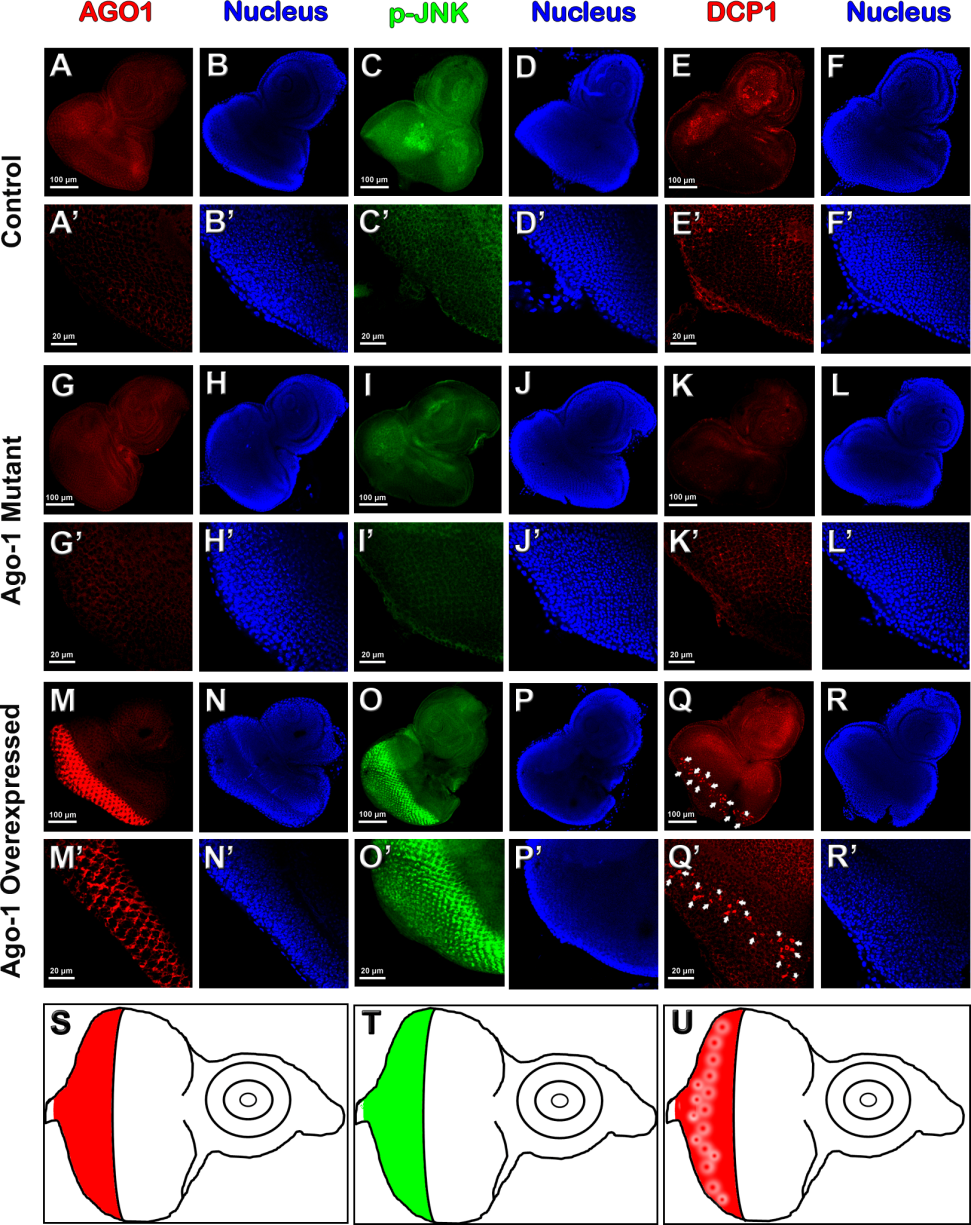
Level of JNK phosphorylation and consequent effector caspase activation with *Ago-1* expression. **(A-F’)** Control group showing normal level of (A, A’) *Ago-1* expression; (C,C’) JNK phosphorylation and (E-E’) Dcp-1 activation. **(G-L’)** *Ago-1* mutant group expressing low level of (G,G’) *Ago-1*, (I,I’) JNK phosphorylation and (K,K’) active Dcp-1 level. **(M-R’)** *Ago-1* over expressed group showing high level of (M,M’) *Ago-1* expression along with (O,O’) elevated JNK phosphorylation and subsequent activation of (Q,Q’) effector caspase, Dcp-1. **(S,T** and **U)** Diagram showing the over expressing area of discs for (S) AGO1, (T) p-JNK and (U) active Dcp-1.

To inspect whether the JNK phosphorylation is induced by *Ago-1*, we produced transient expression of *Drosophila Ago-1* in adult eyes using eye specific GMR-GAL4 driver and checked the expression of phospho-JNK (active form of JNK) by performing western blot analysis with proteins isolated from the adult eyes of the overexpressed *Ago-1* flies. Significant increase in the expression of phospho JNK was seen in flies carrying overexpressed *Ago-1* compared to control (Fig 2I) providing strong evidence that phospho JNK is a physiological target of *Ago-1*. Furthermore, to see the association of JNK with AGO1 we performed immunofluorescent staining (co-localization) using anti AGO1 and anti phospho-JNK antibodies in the eye imaginal discs isolated from late third instar larvae arising from flies carrying over expressed *Drosophila Ago-1 (UAS Ago-1; P{w[+mW*.*hs] = GawB}32B*). These larvae were collected from fly stock produced by crossing *UAS Ago-1* lines to imaginal disc specific GAL4 [*P{w[+mW*.*hs] = GawB}32B*] lines that produced a specific expression only in the 3^rd^ instar larval imaginal disc. A clear activation of JNK in the area of AGO1 overexpression in the eye-antennal imaginal discs of the larvae, confirmed that AGO1 is an activator of JNK (Fig 2IIA–2IIE’”). To check the phosphorylation status of JNK in developing eye we further performed immuno staining studies using p-JNK antibodies in the eye disc of *UAS Ago-1/GMR GAL4* larvae. Simultaneously the same eye discs were double stained with anti AGO1 antibody to visualize and confirm the level of co-expression of AGO1 and p-JNK protein in eye discs isolated from *Ago-1* over expressed lines (Fig 2IIIA–2IIIE”). Staining performed with the same antibodies in eye discs isolated from larvae produced by crossing *UAS Ago-1* with *GMR GAL4* to induce over expression line and *Ago-1* mutant (*UAS Ago-1/GMR GAL4 and P*{*hs-Ago-1*}; *Ago-1*^*k08121*^/*Ago-1*^*k08121*^) along with *GMR GAL4* stocks, served as control. There was a significant increase in the expression and association of p-JNK antibody in eye discs from *Ago-1* overexpressed lines compared to control and mutant lines. As expected, AGO1 anti body showed an increased level of expression of AGO1 in the discs isolated from *Ago-1* over expressed lines (Fig 3 and S4 Fig). Taken together the results clearly confirmed that phosphorylation of JNK is indeed induced by *Ago-1*.

#### Hep activation and JNK phosphorylation by *Ago-1* is related to *Tak1* expression

**Fig 4:**
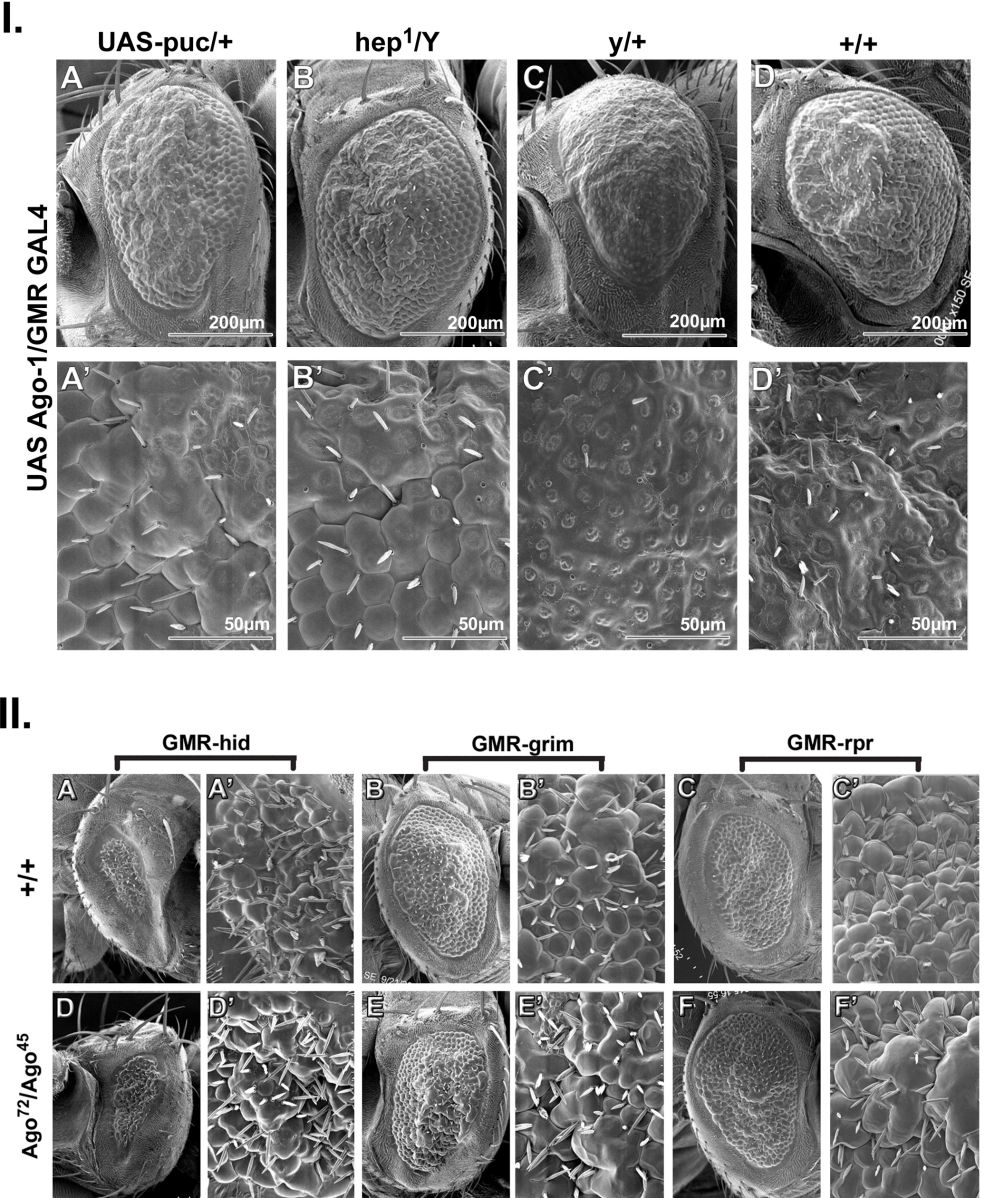
**I.** *puc* over expression and *hep* mutation prevent *Ago-1* induced apoptosis. (**A &A’**) Partial recovery of eye phenotype observed as a result of ectopic *puc* over expression along with *Ago-1*. (**B-B’**) *hep* mutation (*hep*^*1*^) producing recovery of *Ago-1* over expression induced apoptotic eye phenotype. Eye phenotype of (**C-C’**) *+/y; UAS Ago-1/GMR GAL4* and (**D-D’***) +/+; UAS Ago-1/GMR GAL4* used as control. **II.** *Ago-1* functions upstream of *hid*, *grim* and *reaper*. Heteroallelic mutation of *Ago-1*(*Ago-1*^*72*^*/Ago-1*^*45*^) can not affect more on *hid*, *grim* and *rpr* over expression induced apoptosis in fly eye. SEM image of adult fly eye with enlarge view to show ommatidia phenptype of (**A-A’**) *GMR-hid*, (**B-B’**) *GMR-grim*, (**C-C’**) *GMR-rpr* and (**D-D’**) *Ago-1*^*72*^*/Ago-1*^*45*^*; GMR-hid*, (**E-E’**) *Ago-1*^*72*^*/Ago-1*^*45*^*; GMR-grim*, (**F-F’**) *Ago-1*^*72*^*/Ago-1*^*45*^*; GMR-rpr*. *hid*, *grim*, *rpr* over expression induced small eye phenotype not changed significantly in over expression of those genes along with *Ago-1* heteroallelic mutation background (*Ago-1*^*72*^*/Ago-1*^*45*^).

Our next question was to see whether *Ago-1* dependent apoptosis is directly regulated via activation of JNK or is dependent on its upstream players that in turn activate JNK. We generated flies that carry *hep* mutation (*hep*^*1*^), homologue of mammalian MKK7 (also known as JNKK) that encodes *Drosophila* MAP Kinase Kinase in *Ago-1* over expressed flies (*hep*^*1*^*; UAS Ago-1/GMR GAL4*). *hep* mutation inhibits JNK phosphorylation and blocks epithelial cell movement required for maintaining the morphogenetic activity. Flies carrying overexpression of *Ago-1* under the background of *hep* mutation produced a partial reversion of eye phenotype (Fig 4I) implicating the requirement of *Drosophila* JNK kinase *hep* in *Ago-1* dependent phosphorylation and activation of JNK. More importantly, suppression of *Ago-1* resulted in induction of apoptosis in eye imaginal discs under *hep* mutant background clearly demonstrating that JNK pathway is indispensable for the *Ago-1* induced apoptosis. Quantitative real time PCR studies and western analysis in *Ago-1* over expressed flies showed a significant increase in the level of expression of *hep* both in RNA (S1 Fig) and protein level (S5 Fig).

**Fig 5:**
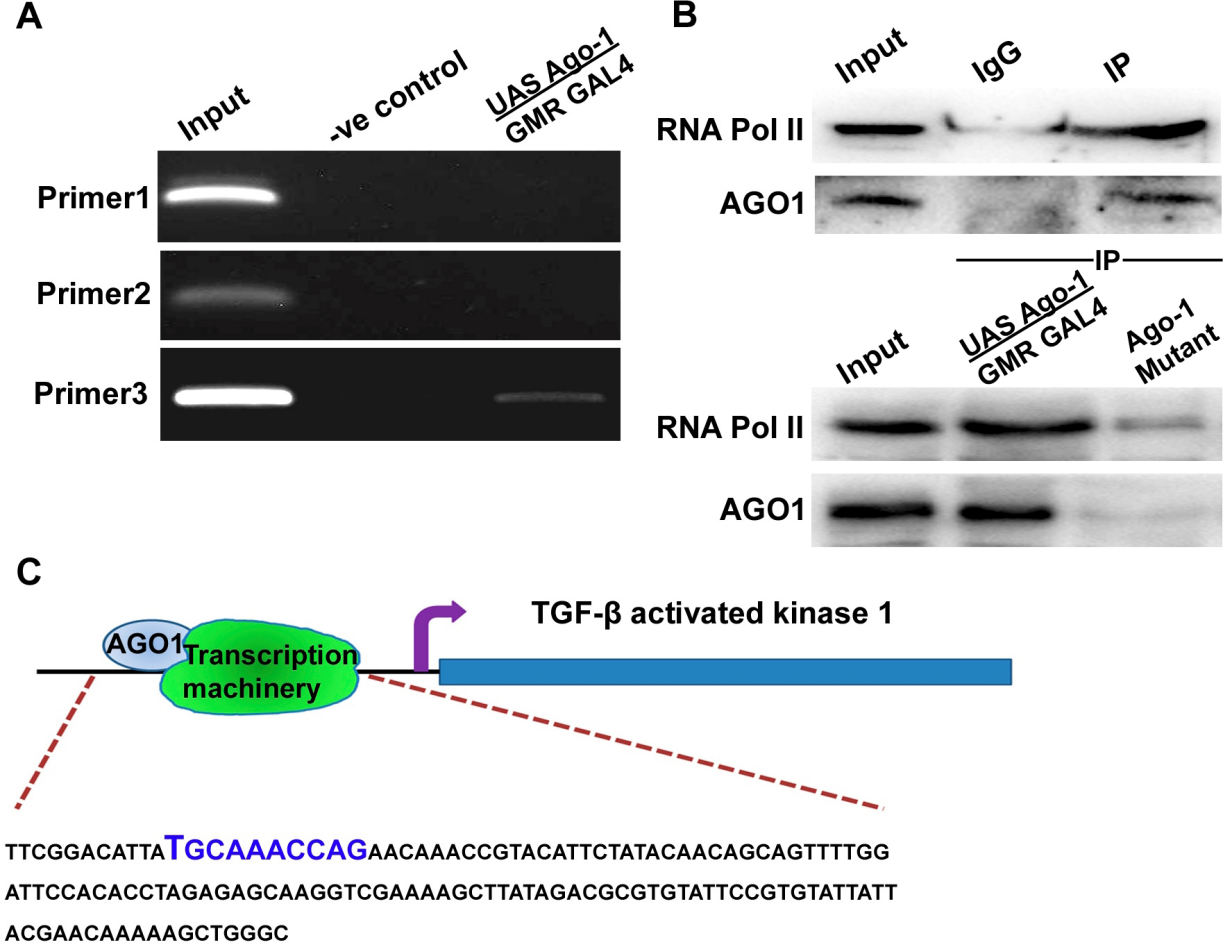
AGO1 interacts with transcription machinery at the promoter region of TGF-β activated kinase 1 (*Tak1*) gene (**A**) Gel image showing bands of amplified regions (predicted promoter regions) of *Tak1* 5’ upstream sequence DNA getting from Chromatin Immuno Precipitation (ChIP) using AGO1 antibody, 5% crosslinked fragmented DNA was used as an input and the sample from “no antibody beads (IgG+beads)” was used as negative control, (**B**) Blot image showing the interaction between nuclear AGO1 and RNA Pol II. Immuno Precipitation (IP) of nuclear extract, extracted from *Ago-1* over expressed eye was carried out using RNA Pol II antibody, followed by detected with RNA Pol II and AGO1 antibody (upper two). Same experiment was performed using nuclear extract, extracted from *Ago-1* over expressed as well as *Ago-1* mutant fly eye (lower two) (**C**) Model figure showing interaction of AGO1 with transcription machinery at the 5’ upstream region of *Tak1* gene.

TAK1 an upstream player of JNKK (*hep*), activates a number of intracellular kinases (p38 MAPK, JNK and IKK) by inducing or inhibiting apoptotic cell death in a number of cells depending upon cell types [28, 29]. Role of TAK1 in the regulation of autophagic cell death has been reported [30]. To explore whether there is any direct involvement of TAK1 we performed both Immuno Precipitation (IP) and ChIP assay using chromatin extracted from *Ago-1* over expressed head & eye. PCR amplification was performed using three different primer sets, designed against predicted (by Neural Network Promoter Prediction tool, http://www.fruitfly.org/seq_tools/promoter.html) promoter sequences. Results demonstrated a significant protein-DNA interaction at upstream sequence of *Tak1* (Fig 5A). Interestingly, IP study with nuclear extract (extracted from *Ago-1* over expressed head along with eye) showed a remarkable interaction of nuclear AGO1 with RNA Pol II (Fig 5B & 5C) concluding the possibility of AGO1 interaction with transcription machinery at *Tak1* promoter region to induce high level of *Tak1* expression which in turn activates its downstream partner *hep* to induce JNK phosphorylation; though possibility may still exist that AGO1 may interact with RNA Pol II on different, other promoters also. Interestingly, *Tak1* mutation under *Ago-1* over expressed background (*UAS Ago-1/GMR GAL4; Tak1*^*ds*^) showed reduced level of JNK phosphorylation (S6 Fig) confirming the involvement of *Tak1* with *Ago-1* over expression induced phosphorylation of JNK.

We next introduced *puc* a known *Drosophila* MAPK phosphatase, that inhibits apoptosis by preventing JNK phosphorylation in *Ago-1* over expressed flies (*UAS Ago-1/GMR GAL4; UAS puc*). Relatively less *Ago-1* induced apoptosis under *puc* over expressed background in fly eye was observed (Fig 4IA and 4IA’) indicating the involvement of *puc* in *Ago-1* induced JNK signaling. Collectively, *Ago-1* activates JNK by the phosphorylation process, which is dependent on JNK kinase *hep* and JNK phosphatase *puc* and controls apoptotic process in *Drosophila melanogaster*.

#### Pro apoptotic gene *hid*, *grim* and *reaper* are downstream to *Ago-1*

**Fig 6:**
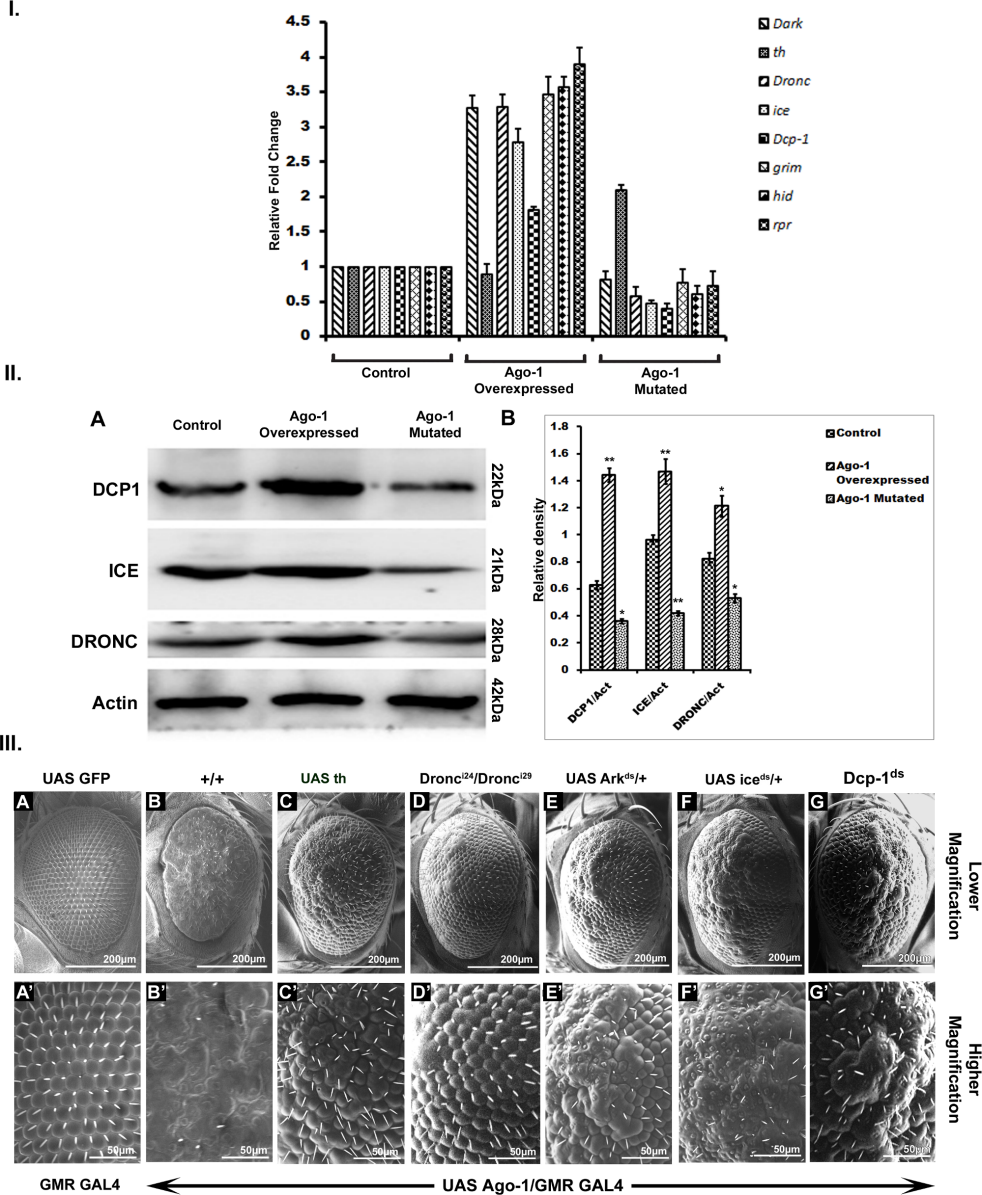
Apoptotic inhibitors and caspases are important for *Ago-1* regulated apoptotic cell death I. Real time PCR analysis showing increased level of expression of pro apoptotic and caspase genes in *Ago-1* over expressed line whereas the same line showing a decreased expression level of *th* gene transcript. II. (**A**) Western blot bands showing activation of both initiator (DRONC) and effector (ICE and DCP1) caspases upon *Ago-1* over expression and reduced level activation was observed in mutant line (**B**) Graphical presentation of western blot band intensities (±SEM) **III. (A, A’)** *GMR GAL4* driven *UAS GFP* carrying adult eye phenotype of *D*. *melanogaster* **(C, C’)** *UAS Ago-1/GMR GAL4* adult eye phenotype **(C, C’)** *th* encodes an apoptotic inhibitor, DIAP-1. Co expression of *th* with high level of *Ago-1* inhibits the apoptotic effect of *Ago-1*. **(D, D’)** Hetero allelic mutation of apical *Drosophila* caspase, *Dronc* with *Ago-1* over expression recovers adult eye phenotype towards wild type. **(E, E’)** *Ark (Dark)* is an ortholog of mammalian Apaf1; deficient mutation of *Ark* reduces the effect of over expressed *Ago-1*. **(F, F’)** Single copy deficient mutations of effector caspase *Drice* (*ice*) cannot rescue *Ago-1* induced eye phenotype significantly. **(G, G’)** Deficient mutation of another effector caspase *Dcp-1* can inhibit *Ago-1* induced apoptosis; but, fails to recover normal phenotype.

Although apoptotic defects found in *Ago-1* mutants do not show exactly similar phenotypes as seen in other *Drosophila* deficient mutants of major pro apoptotic genes, such as *hid*, *grim* and *rpr* but activation of apoptotic initiator also induced extensive activation of caspases. Therefore we speculated probability of pro apoptotic genes involvement in *Ago-1* induced apoptosis. We therefore introduced one mutant copy of *hid* gene (*hid*^*H109*^) into *Ago-1* over expressed flies and observed the eye phenotype under SEM and also checked the expression of RNA and proteins. Interestingly, reducing the dose of *hid* (*hid*^*H109*^) prevented *Ago-1* over expressed induced apoptosis significantly (Fig 1IIIF & 1IIIF’). However, the trans-heterozygotic mutation of *Ago-1* (*Ago*^*72*^*/Ago*^*45*^) produced minimum effect on *hid* induced apoptosis. Further, this trans-heterozygotic mutation was not capable of reversing the *hid*, *grim* and *rpr* induced apoptosis significantly (Fig 4IIA–4IIF’), implicating that *Ago-1* exists upstream of these pro apoptotic genes. A similar pattern of expression was observed at both the transcript (Fig 6I) and protein level. Further immuno staining under similar *Ago-1* over expression showed a clear induction of HID, GRIM and RPR (S7 Fig) Taken together these results clearly indicate that *hid*, *grim*, *rpr* function downstream to *Ago-1* in JNK pathway induced apoptotic process.

#### *Ago-1* over expression activates an IAP-sensitive cell death pathway

In *Drosophila*, initiation of apoptosis is mainly controlled by *hid*, *reaper* and *grim* who share a short region of homology at the N terminus which interacts with DIAP-1 to regulate its function negatively [31]. *thread* (*th*) gene encodes *Drosophila* Inhibitory apoptotic protein, DIAP1 which functions similar to the inhibitor of apoptosis protein. Evolutionarily conserved DIAP1 is homologous to baculovirus IAPs and also share common domain with mammalian IAPs and prevents apoptosis [32]. To investigate the role of DIAP-1 in inducing cell death under *Ago-1* over expression, we inserted UAS carrying *th* allele (*UAS th*) in flies having over expression of *Ago-1* (*UAS Ago-1/GMR GAL4; UAS th*). Over expression of *th* rescued several ommatidia that were lost as a result of *Ago-1* over expression induced cell death in developing eye (Fig 6IIIC, 6IIIC’ and S8 Fig). Low level of expression of *th* transcript was observed in *Ago-1* over expressed background (Fig 6I), whereas with the introduction of *UAS th* in the same *Ago-1* over expressed flies recovered the expression of *th* transcript as well as the expression level of DIAP protein.

#### *Ago*-*1* induced apoptosis is caspase dependent

**Fig 7:**
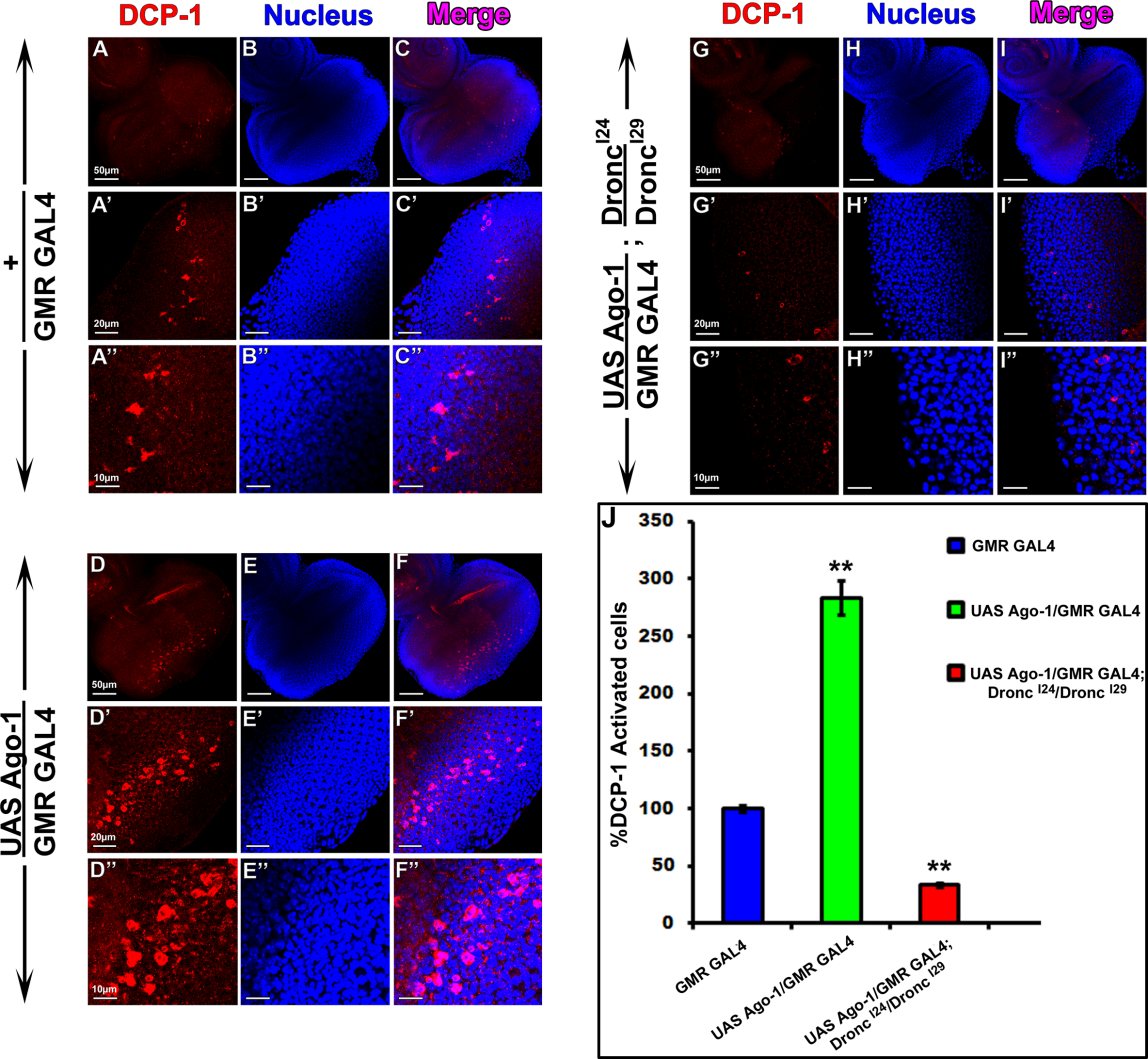
*Ago-1* over expression induces high level of DCP-1 activation in developing fly eye. *Dronc* mutation (*Dronc*^*I24*^*/ Dronc*^*I29*^) resulted inhibition of *Ago-1* induced DCP-1 activation. **(A-C”)** Normal activation of DCP-1 in developing eye of control disc. **(D-F”)** *Ago-1* over expression resulted increased level of DCP-1 activation **(G-I”)** Dronc mutation (*Dronc*^*I24*^*/ Dronc*^*I29*^) inhibits *Ago-1* over expression induced high level of DCP-1 activation. **(J)** Bar diagram shows the percentage of DCP-1 activated cells in 3^rd^ instar eye imaginal discs; genotype mentioned in the figure.

CARD-containing *Drosophila* caspase DRONC or Nedd2-like caspase are protein factors that regulate programmed cell death during *Drosophila* development. DRONC which is under regulation of DIAP1 [33] act as ICE interacting caspases to activate downstream apoptotic processes [34, 35]. To understand the role of *Dronc* in *Ago-1* induced cell death, trans-heterozygous *Dronc* mutant flies were generated (*Dronc*^*I24*^*/Dronc*^*I29*^) along with *Ago-1* over expression {(*UAS Ago-1/GMR GAL4; Dronc*^*I24*^*/Dronc*^*I29*^; homozygous double mutants (*Dronc*^*I24*^*/ Dronc*^*I24*^ or *Dronc*^*I29*^*/Dronc*^*I29*^) are lethal}. Introduction of trans-heterozygous *Dronc* mutant produced almost near to full recovery of rough eye phenotype (Fig 6IIID and 6IIID’) indicating a clear involvement of *Dronc* in *Ago-1* over expression induced cell death. To observe the involvement of *Ark* (*Dark*), a homolog of mammalian Apaf-1 and *C*. *elegans* CED-4 cell death protein, we introduced RNAi induced silenced *Dark* gene in *Ago-1* over expressed flies (*UAS Ago-1/GMR GAL4; Ark*^*ds*^). Heterozygous flies carrying silenced *Ark* partly recovered the eye phenotype towards normal (Fig 6IIIE and 6IIIE’), whereas, introduction of a single copy of RNAi silenced *Drice (ice)*, a *Drosophila* effector caspase in *Ago-1* over expressed flies failed to recover *Ago-1* induced apoptotic cell death phenotype in fly eye (Fig 6IIIF and 6IIIF’). An increased level of both ice RNA and ICE protein in *Ago-1* over expressed line indicated clear involvement of *ice* in *Ago-1* induced apoptosis. But, failure to suppress the *Ago-1* induced apoptosis by *ice* mutant suggests the involvement of other effector caspase. Expression studies of *Dronc* transcript (Fig 6I) and protein (Fig 6IIA and 6IIB) showed an increased level of expression in *Ago-1* over expressed flies. Immuno staining assay in eye imaginal discs isolated from the growing third instar larvae arising from *Ago-1* over expressed stocks showed an increased level of initiator caspase activity (S9 Fig). On other hand, eye discs isolated from larvae carrying heterozygous mutation of CARD domain containing *Drosophila* caspase, *Dronc* (*Dronc* ^*I24*^*/Dronc*^*I29*^) along with *Ago-1* over expression showed reduced level of expression indicating it’s incapability of inducing apoptosis significantly. These observations clearly demonstrate the involvement of caspases in *Ago-1* induced apoptosis. To identify involvement of other caspases in *Ago-1* induced apoptosis, we studied the expression of effector caspase *Drice* both at transcript and protein level. Although *Drice* level was slightly increased in *Ago-1* over expressed flies both at transcript (Fig 6I) and protein level (Fig 6II), the genetic data showed that single copy RNAi silenced *Drice* with *Ago-1* over expression was unable to recover *Ago-1* induced apoptotic cell death (Fig 6IIIF and 6IIIF’) indicating the involvement of other effector caspases in this process. We therefore checked the transcript and protein expression of *Dcp-1*, another effector caspase known to be actively involved in apoptotic process [36, 37]. *Ago-1* over expressed flies showed more expression of Dcp-1 mRNA (Fig 6I) along with high cleavage level of Dcp-1 protein (Fig 6II). To confirm the elevated level of cleaved Dcp-1 under *Ago-1* overexpression, we performed immunostaining experiments in wing discs of larvae arising from *ptc GAL4* driven *Ago-1* over expressed stocks. These wing discs expressed high level of cleaved Dcp-1 at AGO1 over expressing areas of the disc (S10 Fig). Interestingly the adult flies arising from this stock also showed loss of one anterior cross vein between 3^rd^ and 4^th^ longitudinal vein (S3 Fig). Similar result of elevated accumulation of active Dcp-1 was also observed in the eye imaginal discs (driven by *GMR GAL4*) isolated from 3^rd^ instar larvae arising from *Ago-1* over expressed lines. A clear decrease in the accumulation of the active Dcp-1 in mutant line (*P*{*hs-Ago-1*}; *Ago-1*^*k08121*^/ *Ago-1*^*k08121*^), confirmed that elevated expression was indeed due to over expression of *Ago-1* (Fig 3E–3Q’ and S4 Fig). Interestingly, increased level of JNK phosphorylation was observed at the same area of eye imaginal discs of *Ago-1* over expression and Dcp-1 activation (Fig 3M–3M’, 3O–3O’, 3Q–3Q’and graphics S, T and U). Together results suggest active involvement of *Ago-1* in JNK phosphorylation and association of JNK phosphorylation with the activation of Dcp-1 in the *Ago-1* over expression induced apoptotic process. Moreover, immune staining results showed few DCP-1 positive cells in eye discs arising from *UAS Ago-1/GMR GAL4; Dronc* ^*I24*^*/Dronc*^*I29*^ larvae (Fig 7). These observations confirm that *Ago-1* induced apoptosis is caspase dependent.

#### *eiger* is not essential for *Ago-1* induced apoptosis

*egr (eiger)*, TNF (Tumor necrosis factor) ligand of *Drosophila* and TRAF1 (TNF-receptor-associated factor1) have been reported as a critical inducer of JNK-dependent apoptotic process [7, 38, 39]. To uncover the role of *Ago-1* in *egr*-induced apoptosis, flies co-expressing inverted repeat induced silencing of *egr* with *Ago-1* over expression (*UAS Ago-1/ GMR GAL4; UAS egr-IR*) were generated. No phenotypic changes were observed upon introduction of *UAS egr-IR* in the eye phenotypes (Panel A and B in S11 Fig) demonstrating no involvement of *egr* in *Ago-1* induced apoptosis. Furthermore, ectopic expression of *egr* in the eye region, driven by GMR-GAL4 induced JNK dependent apoptosis (Panel C in S11 Fig) was also not reversed upon introduction of RNAi induced down regulation of *Ago-1* (Panel D in S11 Fig), suggesting that *Ago-1* is not essential for ectopic *egr*-induced apoptosis in *Drosophila* development. Moreover studies at the transcript level showed that *Ago-1* over expression produced no change in *dTraf1* and *msn* (*misshapen*) other component of *egr* signaling pathway (Panel E in S11 Fig). Collectively, results demonstrate that there is no direct involvement of *egr* in *Ago-1* induced apoptosis and *Ago-1* is not essential for *egr/TRAF1* induced apoptosis.

#### Endogenous *Ago-1* is necessary for developmental apoptotic process

**Fig 8:**
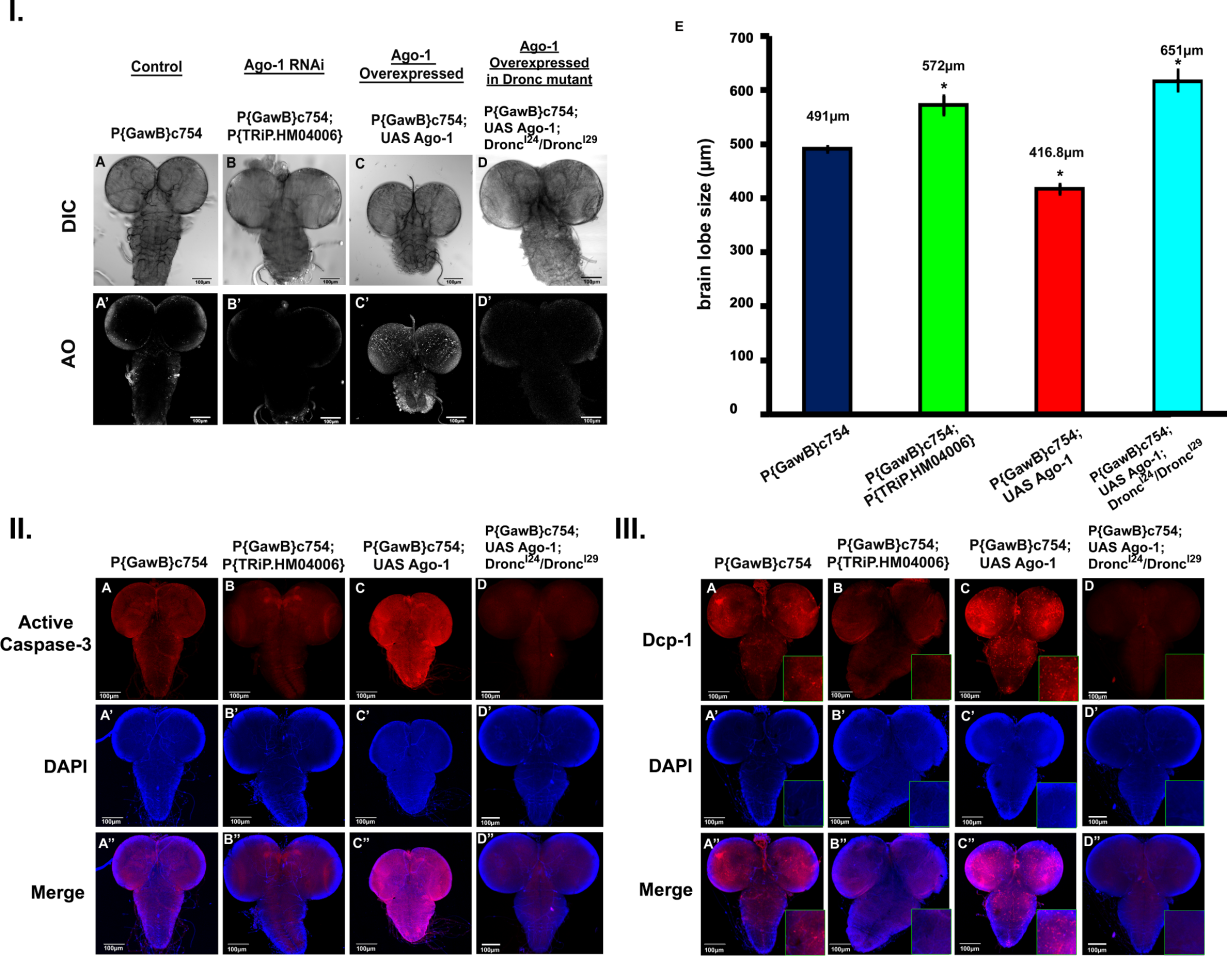
**I.** Endogenous *Ago-1* is important for proper developmental apoptosis. **(A)** Normally developed brain with proper size from *Drosophila* 3^rd^ instar larva, **(A’)** Acridine Orange (AO) staining showing apoptotic cell population in developing brain of normal 3^rd^ instar larva. **(B)** 3^rd^ instar larval brain of *Ago-1* RNAi mutant group showing relatively larger brain size compared to normal, as a result of apoptotic depletion. **(B’)** Reduced number of apoptotic cells in the same brain as depicted by AO staining. **(C)** *Ago-1* over expression resulted in smaller sized brain **(C’)** Increased apoptotic cell population (AO staining) in developing brain lobe. **(D)** *Ago-1* over expression can’t recover Dronc mutation induced brain hyperplasia. **(D’)** Very reduced number of apoptotic cell population (AO staining) was noticed in developing brain lobe of same genotype (mentioned in the figure) larvae. **(E)** Graphical representation of brain lobe size from all four types of fly larvae.**II.** Expression of *Ago-1* is essential for activation of initiator caspase, DRONC **(A)** Normal level of caspase activation in *Drosophila* 3^rd^ instar larval brain. **(A’ & A'')** DAPI and Merge image of the same brain. **(B)** 3^rd^ instar larval brain from *Ago-1* RNAi mutant group showing less activation of caspases compared to control, **(B’)** Same brain stained with DAPI and **(B'')** Merge. **(C)** Over expression of *Ago-1* in larval brain by larval brain specific GAL4 results increased caspase activation in developing brain. **(C')** DAPI stained area of the same brain. **(C”)** Merge Figure. **(D)** Over expression of *Ago-1* in Dronc mutant (*Dronc*^*I24*^*/ Dronc*^*I29*^) larval brain by larval brain specific GAL4 can’t induce efficient caspase activation in developing brain. **(D')** DAPI stained area of the same brain. **(D”)** Merge Figure**. III.** Expression of *Ago-1* is necessary for proper activation of *Drosophila* effector caspase Dcp-1. (A-A”) Normal level of Dcp-1 activation in response to normal *Ago-1* expression. (B-B”) Silencing of *Ago-1* expression using larval brain specific GAL4, reduces Dcp-1 activation in developing brain. (C-C”) Ectopic over expression of *Ago-1* activates Dcp-1 expression in developing brain. (D-D”) Ectopic over expression of *Ago-1* in Dronc (*Dronc*^*I24*^*/ Dronc*^*I29*^) mutant developing brain fails to activate Dcp-1 to induce proper apoptosis in developing organ.

As apoptosis is a crucial process throughout development in maintaining organ size and shape [40], our next question was to see whether endogenous *Ago-1* is required to regulate the developmental apoptosis and thereby control organ size in *Drosophila*. We therefore generated an RNAi mutant *Ago-1* stock that suppresses *Ago-1* expression in larval brain by crossing *P{TRiP*.*HM04006}attP2* (BSC#31700) flies with larval brain and fat body specific GAL4- *P{GawB}c754* (BSC#6984) flies. Larval developing brain, eye and wing imaginal discs from growing 3^rd^ instar larvae arising from the *Ago-1* over expressed stock, mutant stock as well as from control stocks were isolated and AO staining was performed. Larval brain from RNAi silenced *Ago-1* mutants showed brain hyperplasia compared to wild type controls; similar to a brain condition as seen in the *Apaf-1* (Apoptotic protease activating factor-1) null mutants which is a key regulator of apoptosis. Similar bigger sized brain was also seen in *Drosophila* 3^rd^ instar larvae, carrying mutation in its initiator caspase, *Dronc* (S12 Fig). In depth view of third instar larval brain lobes along with ventral ganglion from wild-type *Ago-1* over expressed and *Ago-1* RNAi mutants showed significantly enlarged brain hemispheres and ventral ganglion in *Ago-1* mutant larvae, compared to those in wild type whereas in *Ago-1* over expressed line the size was significantly smaller (Fig 8IA–8IC’ and 8IE). Moreover, results from AO staining and flow cytometry showed cells from brain hemispheres and ventral ganglion of *Ago-1* over expressed line with more apoptotic population as compared to cells from mutant brain (Fig 8IA’–8IC’ and 8IE and S13 Fig). Ectopic expression of *Ago-1* was capable of suppressing the *Ago-1* deficient big brain phenotype to normal size suggesting that the brain hyperplasia produced by *Ago-1* silencing was indeed due to lack of proper *Ago-1* activity.

Larval brain, dissected from *P{GawB}c754* (served as control), *P{GawB}c754; P{TRiP*.*HM04006}attP2* (*Ago-1* RNAi mutant) and *P{GawB}c754; UAS Ago-1* (*Ago-1* over expressed) larve were immunostained with human cleaved caspase-3 antibody (which indicates Dronc activity in fly [41]) and fly specific effector caspase, Dcp-1 antibody. High level of both initiator (Dronc) and effector (Dcp-1) caspase activation was observed in *Ago-1* over expressed developing brain (Fig 8IIA–8IIC” and 8IIIA–8IIIC”) providing a clear evidence that endogenous *Ago-1* is highly essential for controlling organ size of the CNS by inducing developmental apoptosis at the time of organogenesis.

Interestingly, *Ago-1* over expression was neither able to change the brain phnotype produced by Dronc mutation (Fig 8ID, 8ID’ and 8IE, S12 Fig) nor was able to change the status of caspase activation (Fig 8IID–8IID” & 8IIID–8IIID”).

#### *Ago-1* regulates miR-14 expression to sensitize cells for apoptosis and control organ development

**Fig 9:**
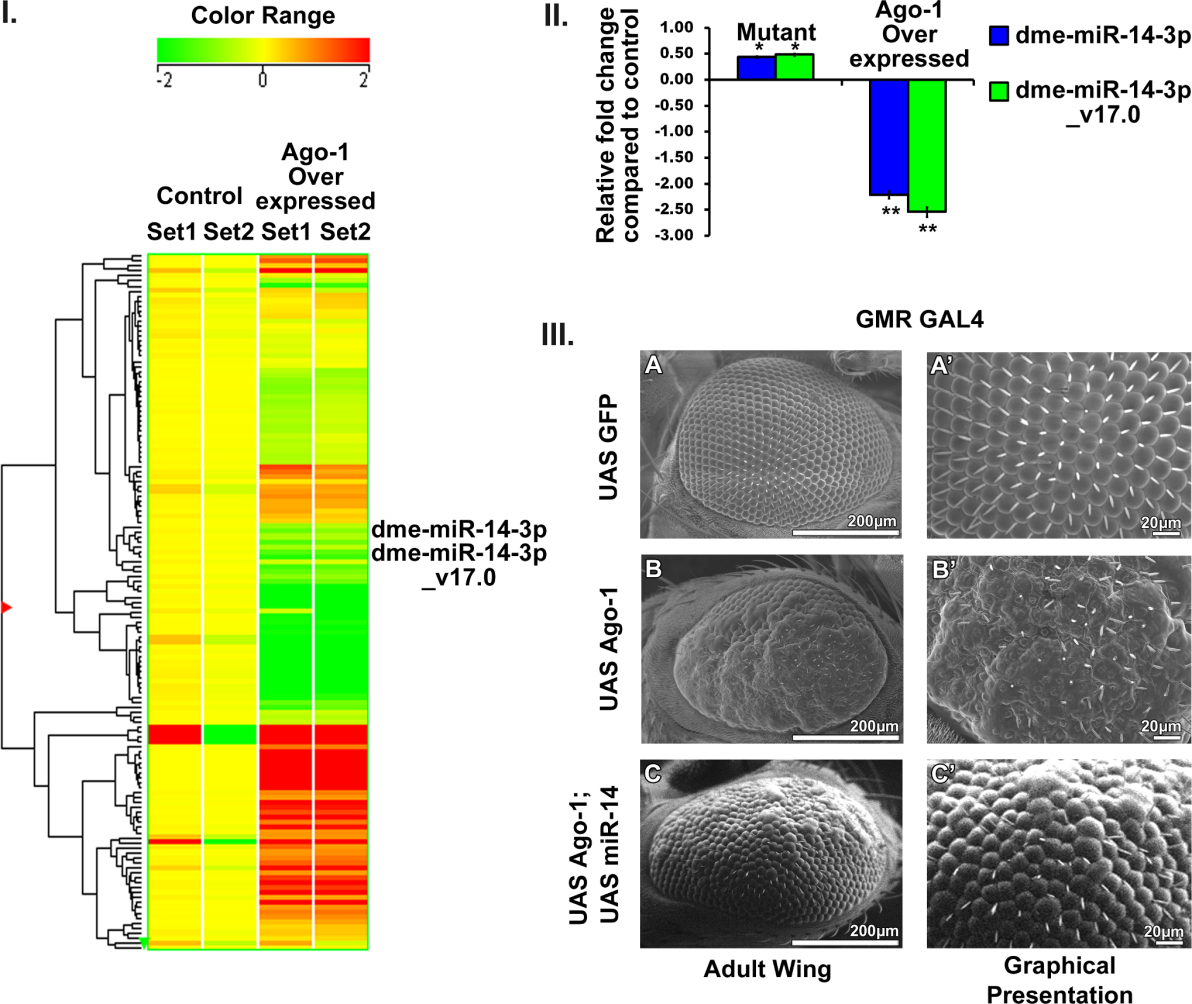
*Ago-1* regulates miR-14 expression to get a better grip on the control of apoptosis. **I.** micro RNA micro array analysis clearly showed a significant down regulation of both miR-14 variant as a result of *Ago-1* over expression. **II.** Up regulation of miR-14 in *Ago-1* mutant and strong down regulation in *Ago-1* over expression was noticed in quantitative PCR analysis. **III.** Ectopic expression of miR-14 successfully rescued the *GMR-GAL4* driven *Ago-1* over expressed phenotype of fruit eye. (**A, A’**) Control eye, (**B, B’**) *Ago-1* over expressed eye, (**C, C’**) *Ago-1* and miR-14 co-expressed ectopically in fly eye.

As miRs are one of the crucial regulators of various cellular processes [42–46], our interest was to see whether any change in their expression occurs under *Ago-1* overexpression. miRNA microarray analysis was carried out using total RNA extracted from control (*GMR-GAL4*) and *Ago-1* overexpressed (*UAS Ago-1/GMR GAL4*) and *Ago-1* mutant (*Ago-1*^*72*^*/ Ago-1*^*45*^) adult eye. miRNAs which has significant difference in their expression are presented in the form of heat map after statistical analysis of the array data (P < 0.05). Several miRNAs were upregulated and several showed down regulation. Interestingly a huge down regulation of mir-14 which is related to apoptosis was observed (Fig 9I and S3 File). miR-14 is expressed throughout development of *Drosophila*. It has already been established as a suppressor of cell death, induced by *hid*, *grim* and *rpr* [47] Further, miR-14 has also been reported as a negative regulator of Dronc-dependent cell death and a regulator of Drice expression either directly or indirectly [47]. Several gene transcripts including Drice, Dcp-1 and grim possesses potential target sites for miR-14 binding (S14 Fig). Drice is essential for cell death events and the apical caspase, Dronc acts as an activator of it, that inflate *rpr*, *hid* and *grim* induced cell death [48, 49]. For further validation of the involvement of miR-14 with *Ago-1* regulated apoptotic cell death quantitative PCR analysis was also performed. A little up regulation of miR-14 expression in *Ago-1* mutant line and high down regulation in *Ago-1* over expressed group was noticed (Fig 9II). Over expression of *Ago-1* produced flies with deformed organ development. Since miR-14 expression was extremely down regulated in *Ago-1* overexpressed lines, we wanted to see whether it plays any role in reverting the organ shape and size to normal when it is over expressed under *Ago-1* over expressed condition. We therefore introduced over expressed miR-14 (*UAS miR-14*) in flies having over expressed *Ago-1*. Interestingly, *GMR-GAL4* driven over expression of miR-14 (*UAS miR-14*) under *Ago-1* over expressed background (*UAS Ago-1*/*GMR-GAL4; UAS miR-14*) successfully recovered *Ago-1* over expression induced eye phenotype to normal (Fig 9III and S15 Fig). Further, flies carrying over expressed *Ago-1* under *sd*-*GAL4* (*scalloped* GAL4) resulted in flies having wings with reduced width compared to normal were also recovered with the introduction of over expressed miR 14 (*sd-GAL4; UAS Ago-1; UAS miR-14*) when driven with *scalloped GAL4* in the wings of adult *Drosophila*. This ectopic expression of miR-14 by *sd-GAL4* successfully rescued *Ago-1* over expression induced wing phenotype (S16 Fig) to nearly normal phenotype. These observations positively suggest that *Ago-1* regulates miR-14 expression to take a better hold in the control of developmental apoptosis in fruit fly.

## Discussion

Programmed cell death is very crucial to maintain proper shape and size of organs by removing excess number of cells. It is also important to eliminate defective and abnormal cells from living system to maintain proper development and activity of organism. JNK pathway is one of the conserved pathways that play a lead role to maintain this activity. Activation of kinase cascade by any intrinsic or extrinsic signal turns on the pathway switch to induce apoptosis.

*Ago-1*, a well conserved protein factor and documented for its role in RISC and RITC complex serve as a RNAi effector complex in RNAi pathway. Apart from this, *Ago-1* plays a role in maintaining proper cellular activity. Here, for the first time we report the role of *Ago-1* in controlling apoptosis during organ development. A cross talk between core RNAi component *Ago-1* and apoptosis via JNK circuit elucidate an in depth mechanism for RNAi role in apoptosis and progammed cell death in *Drosophila*. In continuity of earlier findings these results provide an explicit explanation of how RNAi factor *Ago-1* controls caspase-dependent apoptosis in developing organs to maintain the proper size and shape of organs. Over expression of *Ago-1* produce less number of ommatidia in adult eye (Fig 1IIC’) and suppression of this phenotype (Fig 1IIIC and 1IIIC’) by dominant negative form of *bsk*, a *Drosophila* JNK (*bsk*^*DN*^) indicates a clear involvement of JNK pathway with developmental apoptotic process. Our study revealed an interesting finding that *Ago-1* promotes cell death and controls JNK phosphorylation by regulating *Drosophila* JNK kinase *hep* through its upstream activator, *Tak1* to trigger the pathway in one hand and down regulate onco-miR miR-14 expression in another hand. In flies, the pro apoptotic genes *reaper*, *grim* and *hid* promotes caspase activation by performing their antagonistic activity against DIAP (*Drosophila* inhibitor of apoptosis proteins). Our findings confirm the involvement of these pro apoptotic genes with *Ago-1* induced apoptosis; as *Ago-1* triggered cell death was suppressed by the co-expression of mutant alleles of these pro-apoptotic genes (*hid*^*H109*^; Fig 1IIIE and 1IIIE’) and co-expression of elevated Diap1 as well (Fig 6IIIC and 6IIIC’). Further, significant recovery of eye phenotype upon introduction of trans-heterozygous mutant of *dronc* (*dronc*^*i24*^*/dronc*^*i29*^) in *Ago-1* over expressed background (Fig 6IIID and 6IIID’) confirms *Ago-1* induced cell death is caspase dependent. Moreover, a clear overexpression of cleaved DRONC, ICE and Dcp-1 level at *Ago-1* over expressed condition further supports the involvement of caspases with *Ago-1* induced cell death. Our findings clearly demonstrate the need of endogenous *Ago-1* expression for proper regulation of apoptosis during organ development. Though *Ago-1* is not a strong inducer of apoptosis like *hid*, *grim*, *reaper* or *Dronc*; it plays a crucial role in fine tuning of apoptosis during development. *Ago-1* mutants accumulate cells due to low level of apoptosis (Fig 8 and S13 Fig) during development. *Ago-1* is required for physiological elimination of cells during development to maintain the cell death process in a well controlled manner.

**Fig 10:**
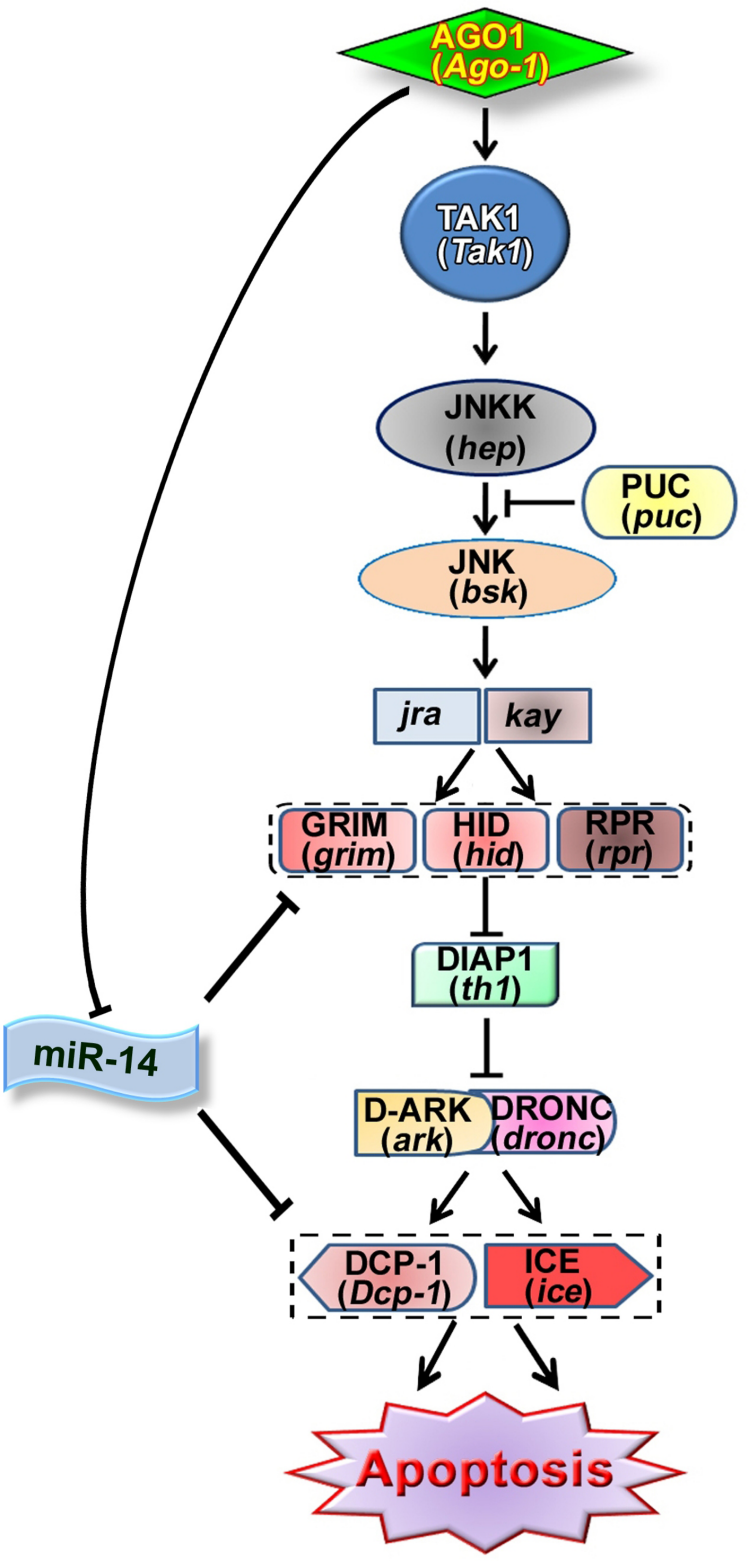
Schematic presentation showing the action mechanism of *Ago-1* to induce apoptosis via JNK pathway.

Development and organogenesis are vital processes wherein nature maintains various control mechanism to eliminate developmental errors. Micro RNAs are also known to play key role in controlling apoptosis. Here, we’ve identified miR-14 as one of the key regulators of *Ago-1* mediated developmental apoptotic process. In *Drosophila*, miR-14 is expressed throughout development and has already been demonstrated as a negative cell death regulator at different stages of insect development by targeting pro apoptotic genes and caspases [47, 50]. Therefore, miR-14 expression equips *Ago-1* to apprehend developmental apoptosis in more disciplined manner. This study sheds a new beam of light on the crosstalk and involvement of *Ago-1* in the induction of cell death through JNK signaling in one hand and through regulating miR-14 in other hand (Fig 10) during organ development. As JNK is implicated in apoptotic process induced by variety of death stimuli including oncogenic transformation and defective transformation in a number of cell types therefore it is clear that JNK signaling plays a pivotal role on tumor formation and metastasis also. Deregulation of this well orchestrated process result in abnormal organ development or progression of oncogenesis. Based on the present study in *Drosophila* it is resolved that, *Ago-1* acts as a regulator of cell death, which is crucial for proper development of organs and might act as a tumour suppressor by inhibiting onco-miR-14 expression as well as by promoting JNK-dependent apoptosis.

## Conclusions

In this study we have uncovered *Ago-1* as a critical regulator of JNK mediated cell death process in *Drosophila melanogaster*. Our genetic and biochemical analysis established *Ago-1* as a potent cell death modulator downstream of *egr*. Moreover, we have shown that *Ago-1* is independent of *egr* to activate JNK and induce JNK dependent apoptotic process through *hep* and *puc*. Furthermore, *Ago-1* acts as a regulator of *puc* through *Tak1* to control JNK phosphorylation and an inducer of JNK dependent cell death. It also regulates miR-14 expression to control the cell death with better control. Evolutionarily conserved nature of *Ago-1* gives a hope that similar process might have a major contribution in modulating JNK-mediated cell death in other animals also.

## Supporting information

S1 Fig Relative fold change of gene expression in *Drosophila* eye under normal and *Ago-1* over expressed condition. (TIFF)

S2 Fig Graphical presentation of (A) ommatidia number in adult eye (n = 20) and (B) percentage of acridine orange (AO) positive cells in eye discs of different (genotype mentioned in the figure) flies.
(TIF)

S3 Fig *Ago-1* over expression induces apoptosis in *Drosophila* wing. *Ptc GAL4* driven *Ago-1* over expression results lack of one cross vein in fly wing (Right panel), where as RNAi down regulation of *Ago-1* in the same region causes relatively thicker cross vein (middle panel) compared to control (left panel).
(TIFF)

S4 Fig High phospho JNK level was detected in the cytoplasmic part of *Ago-1* over expressed eye discs. P-JNK is very low in mutant disc. Phospho JNK level is very high in the AGO1 over expressing part of the fly disc and the same region showing the more activation of *Drosophila* effector caspase DCP-1 (C-D’).
(TIFF)

S5 Fig High level of TAK1 and JNKK was observed in *Ago-1* over expressed line. (TIFF)

S6 Fig Tak1 mutation (*Tak1*^*ds*^) results reduced level of JNK phosphorylation in *Ago-1* over expressed eye disc. (TIF)

S7 Fig HID, GRIM and RPR is induced by *Ago-1* expression in *Ago-1* over expressed eye disc. (TIFF)

S8 Fig Graphical presentation of fully formed ommatidia in adult eyes of different flies (genotype mentioned in the figure). (TIFF)

S9 Fig *Ago-1* over expression results caspase activation in fly eye disc. Eye discs dissected from *Ago-1* over expressed (by eye specific GAL4 line- *GMR GAL4*) 3^rd^ inster larvae and *GMR GAL4* larve (used as a control) and probed using Human active caspase-3 antibody which actually reflects *Drosophila* initiator caspase, DRONC activity. Figure showing high level of DRONC activity as a result of *Ago-1* over expression.
(TIFF)

S10 Fig *Ago-1* over expression induces Dcp-1 activation in *Drosophila* wing disc. *Ptc GAL4* driven *Ago-1* over expression results increased AGO1 expression and Dcp-1 activation (Right panel), where as RNAi down regulation doesn’t show that pattern of expression (middle panel) compared to control (left panel).
(TIF)

S11 Fig *Ago-1* and *egr* are independent of each other to induce apoptosis. (A) Eye phenotype of *Ago-1* over expressed line (B) Same with egr silenced line. (C) Eye phenotype of *egr* over expressed fly and (D) *Ago-1* RNAi silenced flies having over expressed *egr* Note: *Ago-1* RNAi silenced flies cannot recover over expressed *egr* induced small eye phenotype. (E) Real time PCR amplification graph indicates, *Ago-1* over expression and mutation can not affect the expression of *dTraf1* and *msn*.
(TIFF)

S12 Fig Bigger sized larval brain (3rd instar larvae) of Hetero allelic Dronc mutant (upper right panel) was observed as a result of reduced apoptotic population (confirmed by Acridine Orange staining, lower right panel) compared to normal control brain (lower left panel). (TIFF)

S13 Fig Annexin V- FITC Apoptotic detection showing very high level of apoptotic cell population in cells isolated from *Ago-1* over expressing *Drosophila* 3^rd^ inster larval brain. Cells from mutant line showing less apoptotic population compared to control. Right side image panels showing morphology of apoptotic cells (captured in the time of flow by 20X objective lense fitted with Amnis Flowsight).
(TIFF)

S14 Fig mRNAs of caspases and pro apoptotic genes with their miR-14 binding sites. The effector caspase, Drice carries the miR-14 binding site at the 3’UTR region of its mRNA; whereas DCP1 has the binding site for the same miR at the 5’ UTR end and pro apoptotic gene, grim mRNA posses the binding location at 3’UTR region.
(TIFF)

S15 Fig Bar diagram of completely formed ommatidia in adult eyes of different flies (genotype mentioned in the diagram). (TIFF)

S16 Fig Ectopic expression of miR-14 successfully rescued the *scalloped-GAL4* (*sd-GAL4*) driven *Ago-1* over expressed phenotype of fruit fly wing. (**A.**) Control wing, (**B**) *Ago-1* over expressed wing, (**C**) *Ago-1* and miR-14 co-expressed ectopically in fly wing. (**A’, B’, C’**) Diagram showing the changes in the adult wing as a result of different gene expression; pink and cyan blue shaded area indicates the lost part of the normal wing as a result of *Ago-1* over expression in the wing (B’).
(TIFF)

S1 File This is the supplemental Materials and methods. (PDF)

S2 File This is supplemental genetic crosses. (PDF)

S3 File This is micro array data. (XLSX)
