# Supplementary figures and images for: Two way controls of apoptotic regulators consign DmArgonaute-1 a better clasp on it
Source: PLoS One. 2018 Jan 31;13(1):e0190548. doi: 10.1371/journal.pone.0190548 (PMC5791970; doi:10.1371/journal.pone.0190548)

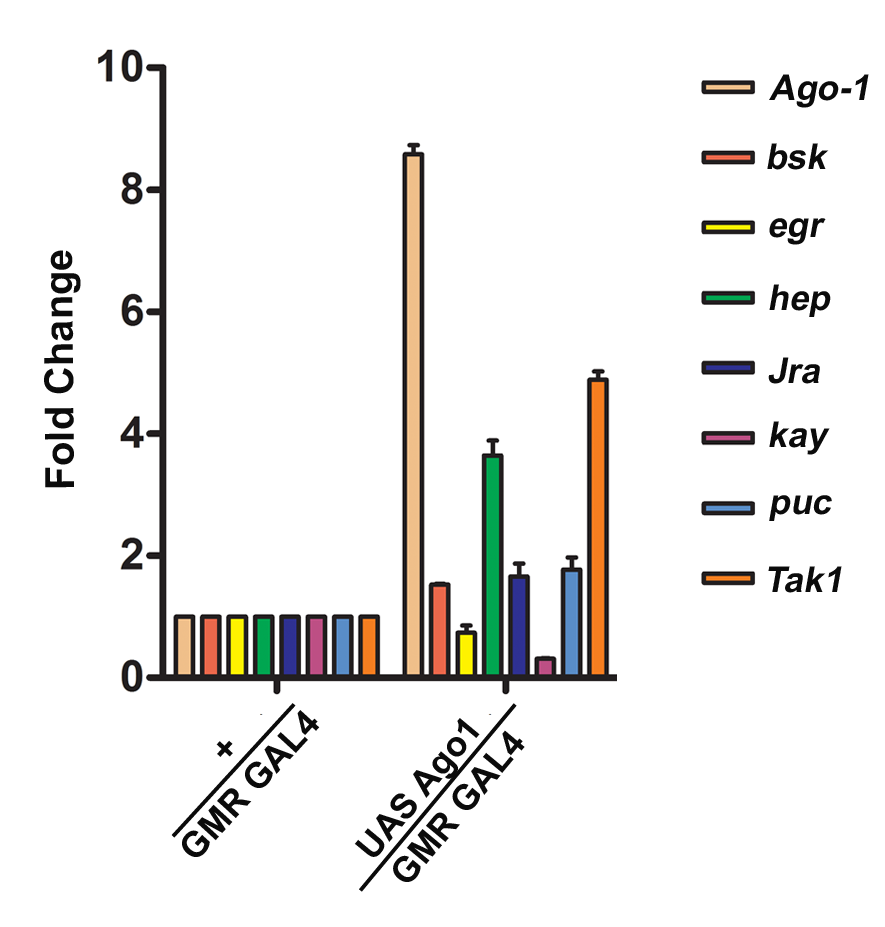

Supplement: S1 Fig — (TIFF) [file pone.0190548.s001.tiff]

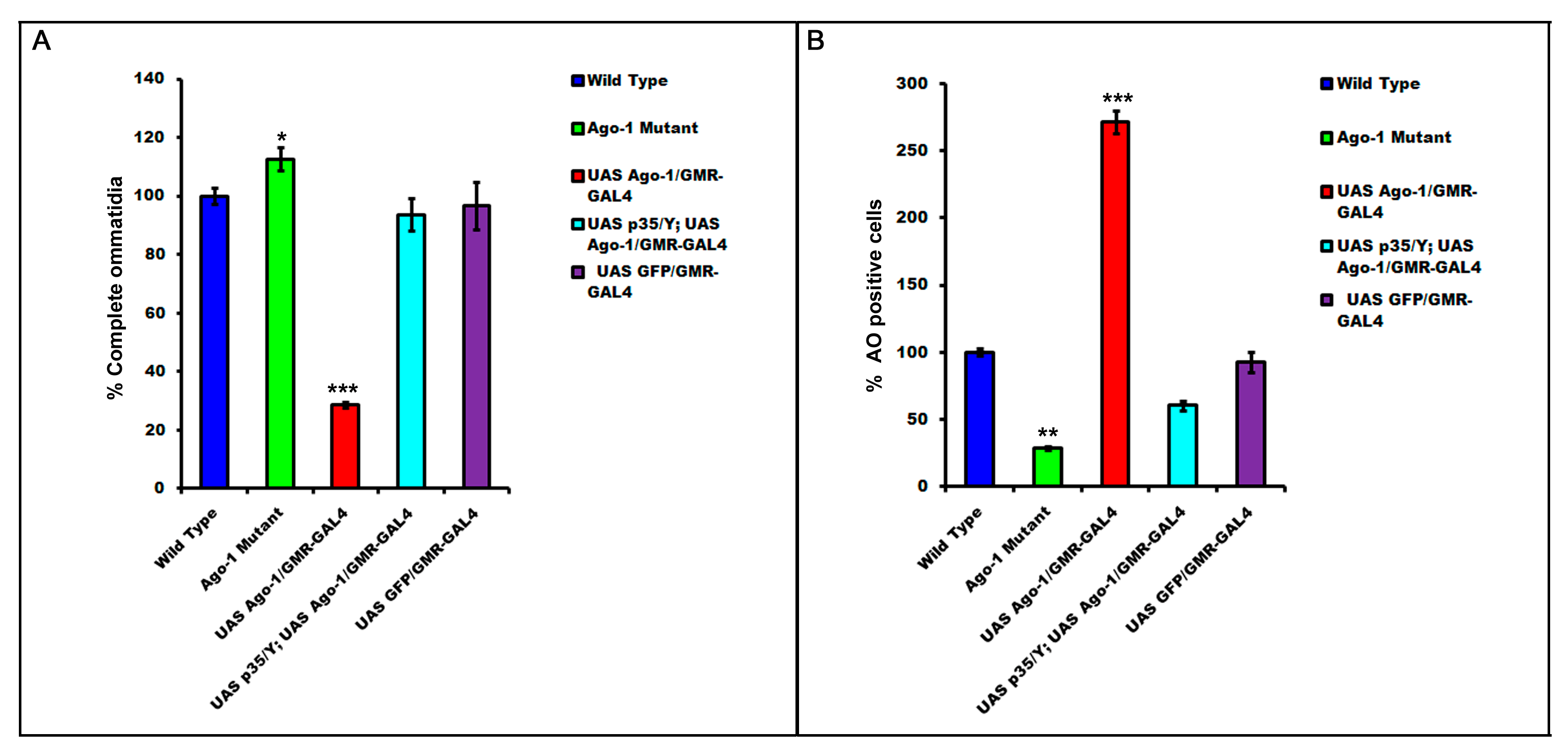

Supplement: S2 Fig — Graphical presentation of (A) ommatidia number in adult eye (n = 20) and (B) percentage of acridine orange (AO) positive cells in eye discs of different (genotype mentioned in the figure) flies. (TIF) [file pone.0190548.s002.tif]

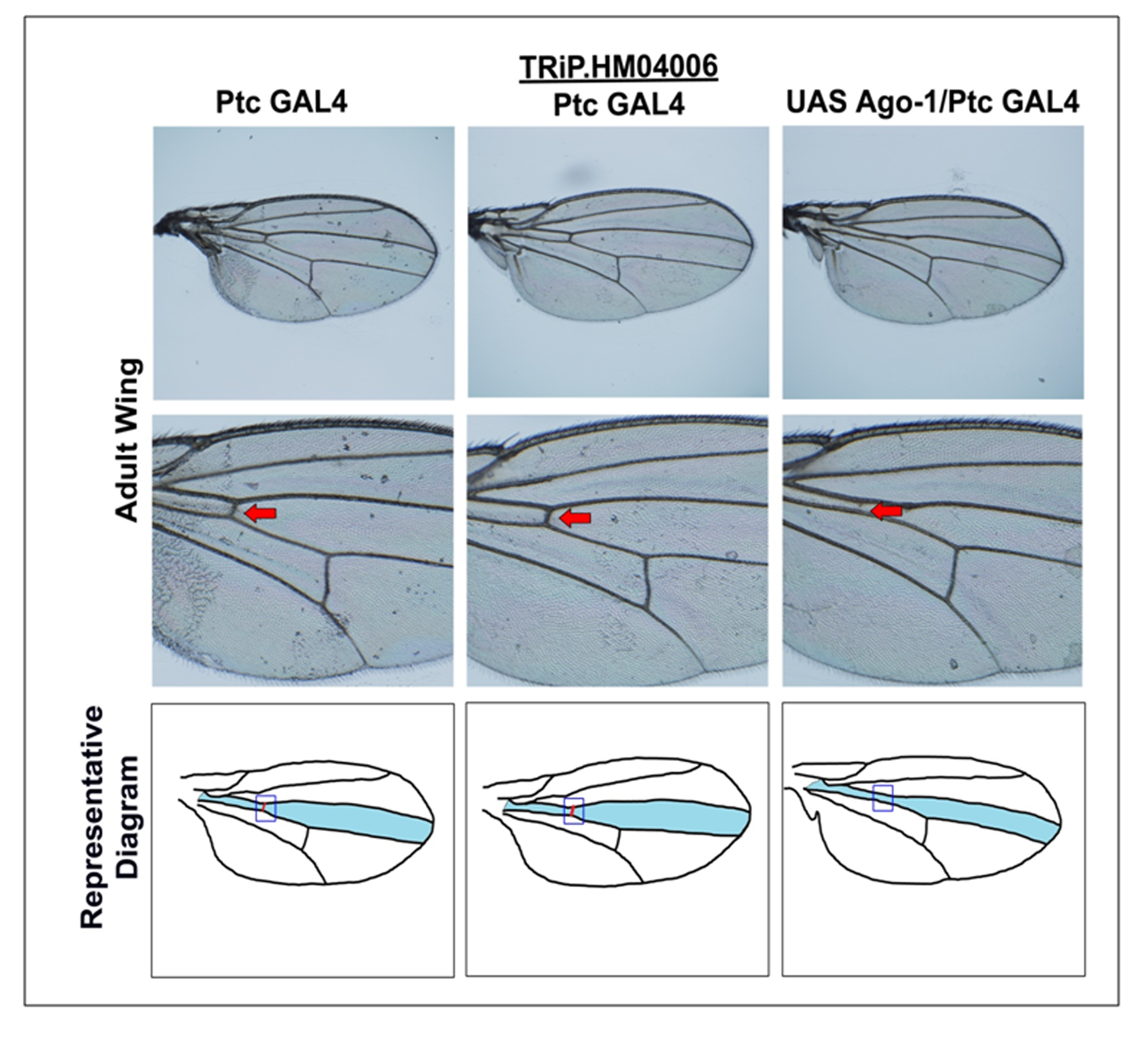

Supplement: S3 Fig — Ptc GAL4 driven Ago-1 over expression results lack of one cross vein in fly wing (Right panel), where as RNAi down regulation of Ago-1 in the same region causes relatively thicker cross vein (middle panel) compared to control (left panel). (TIFF) [file pone.0190548.s003.tiff]

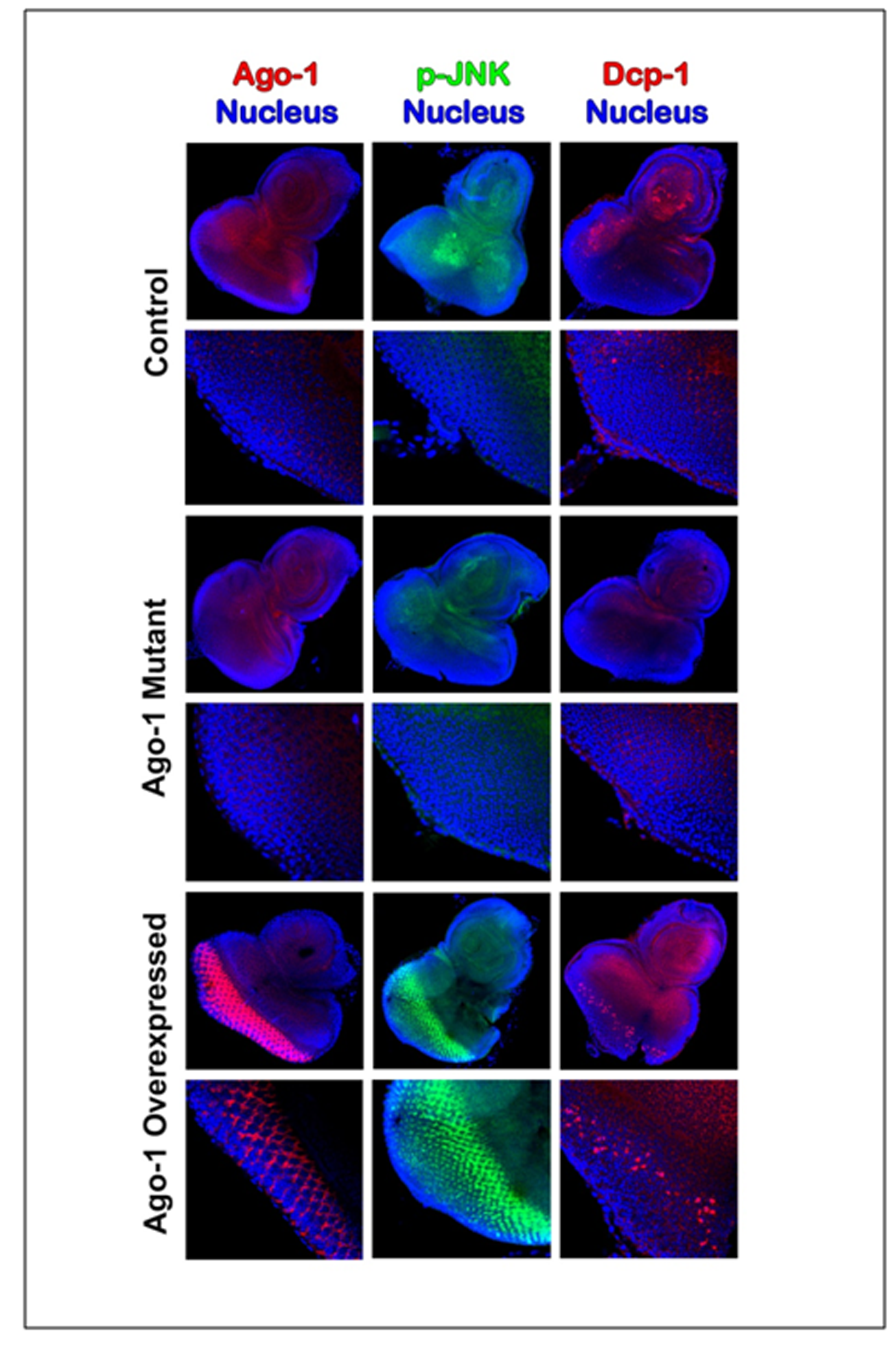

Supplement: S4 Fig — P-JNK is very low in mutant disc. Phospho JNK level is very high in the AGO1 over expressing part of the fly disc and the same region showing the more activation of Drosophila effector caspase DCP-1 (C-D’). (TIFF) [file pone.0190548.s004.tiff]

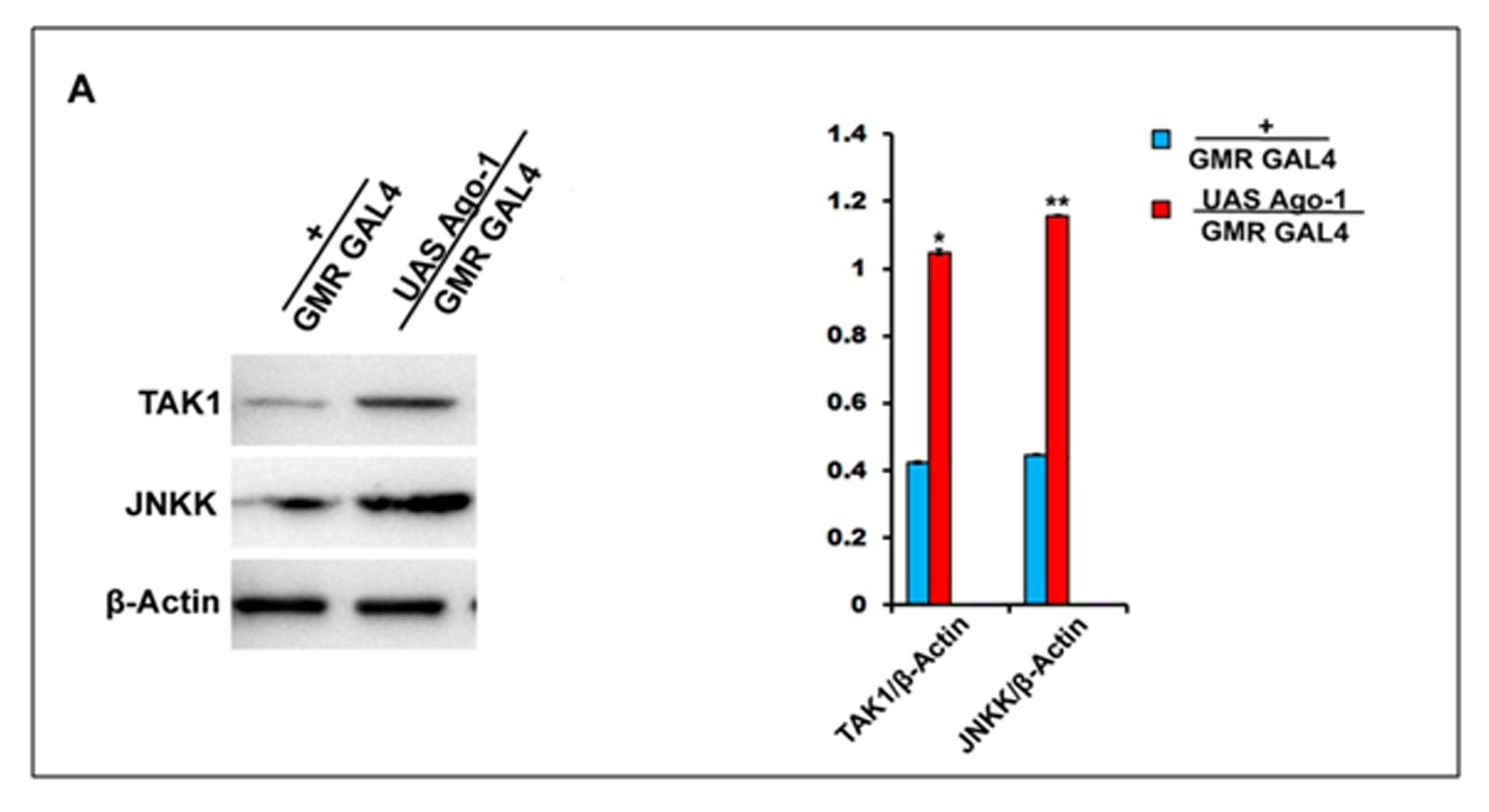

Supplement: S5 Fig — (TIFF) [file pone.0190548.s005.tiff]

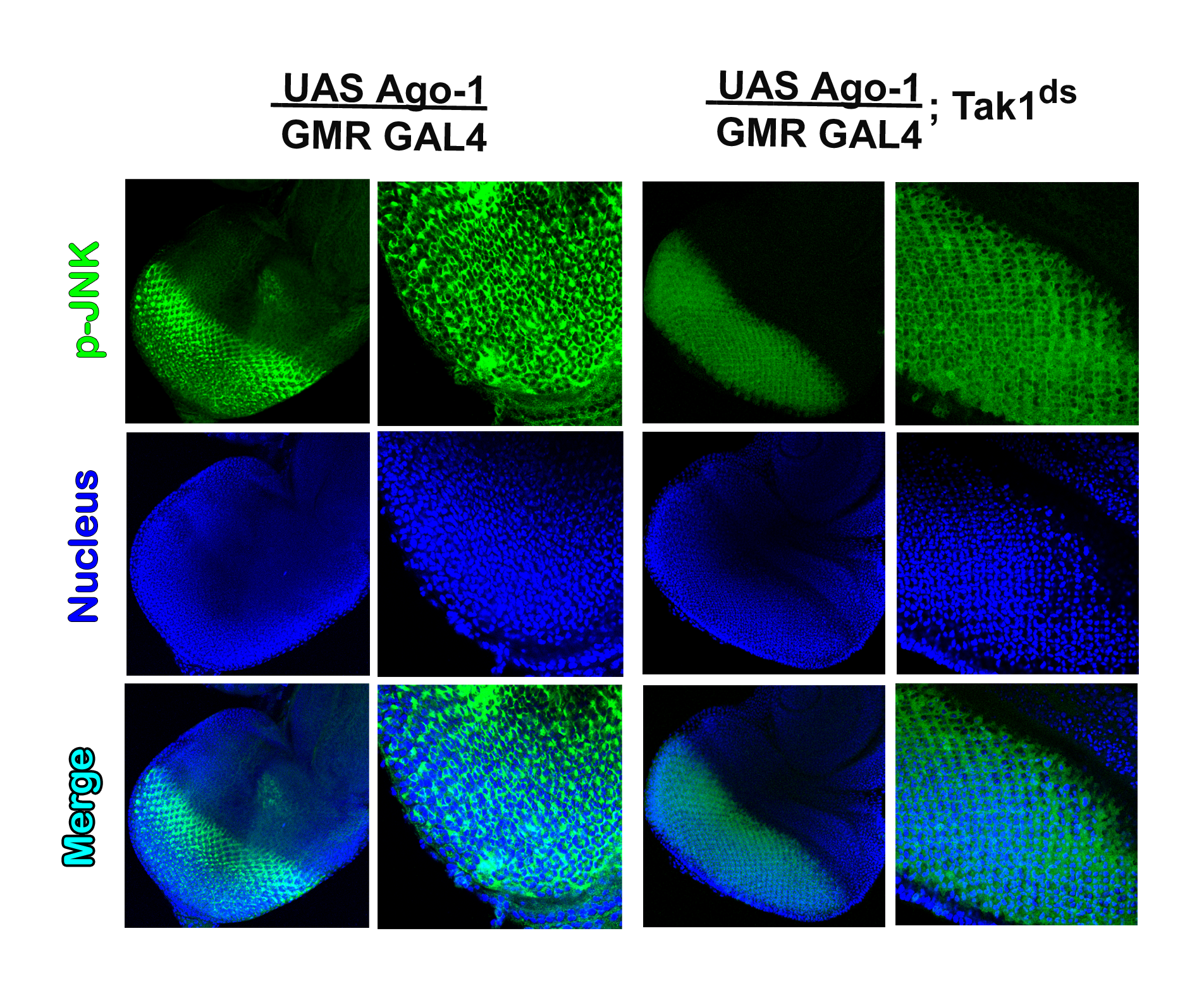

Supplement: S6 Fig — (TIF) [file pone.0190548.s006.tif]

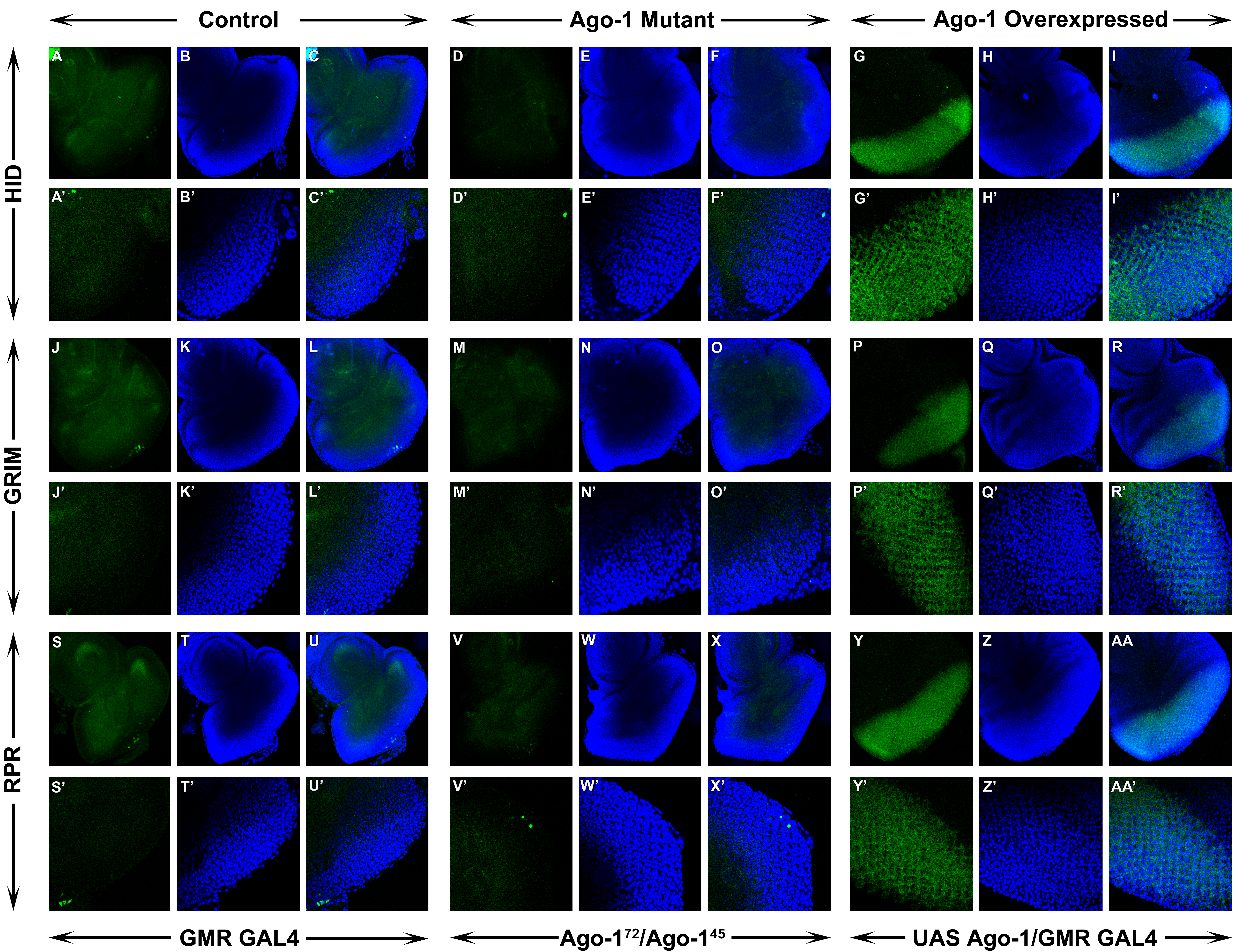

Supplement: S7 Fig — (TIFF) [file pone.0190548.s007.tiff]

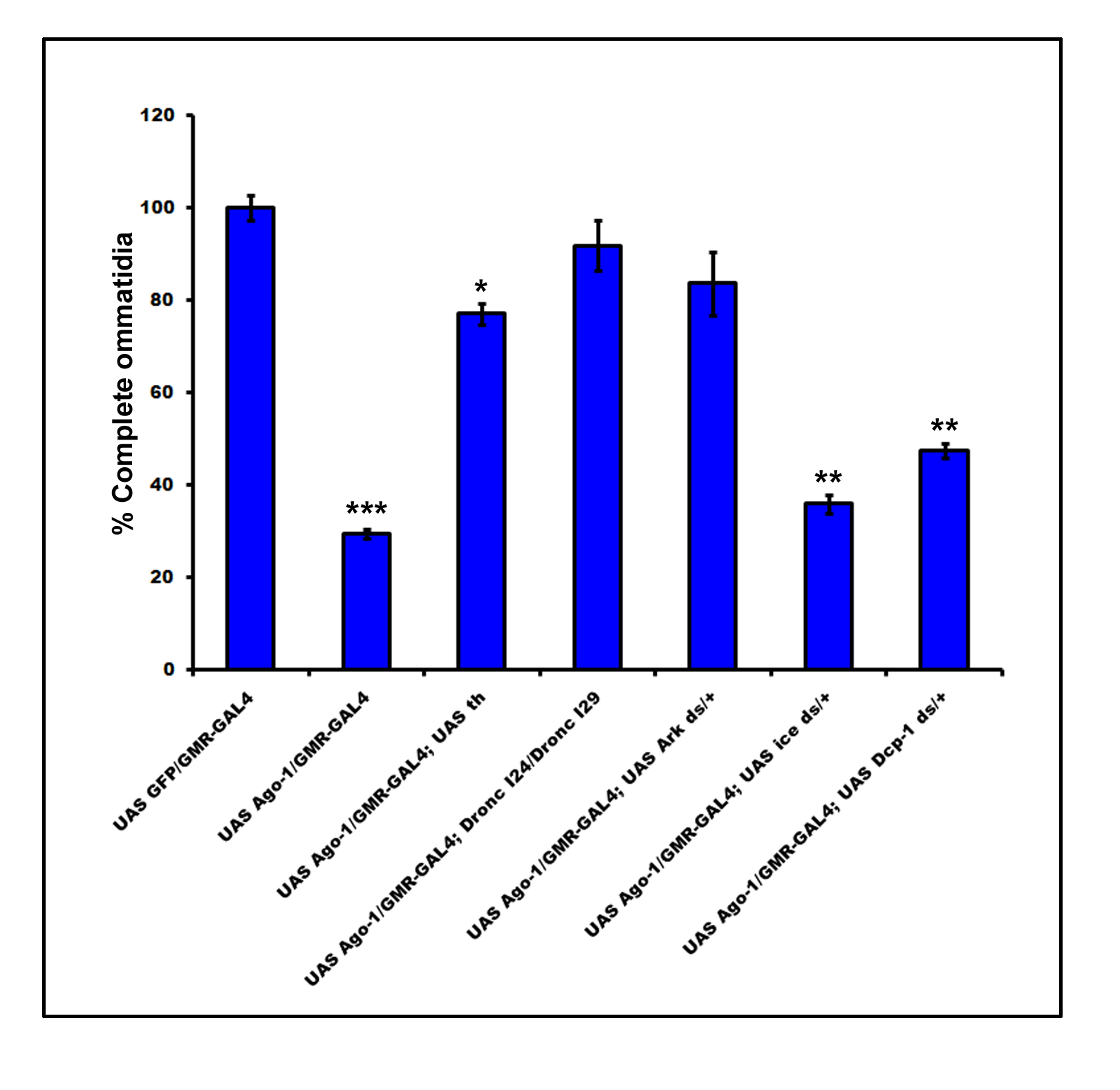

Supplement: S8 Fig — (TIFF) [file pone.0190548.s008.tiff]

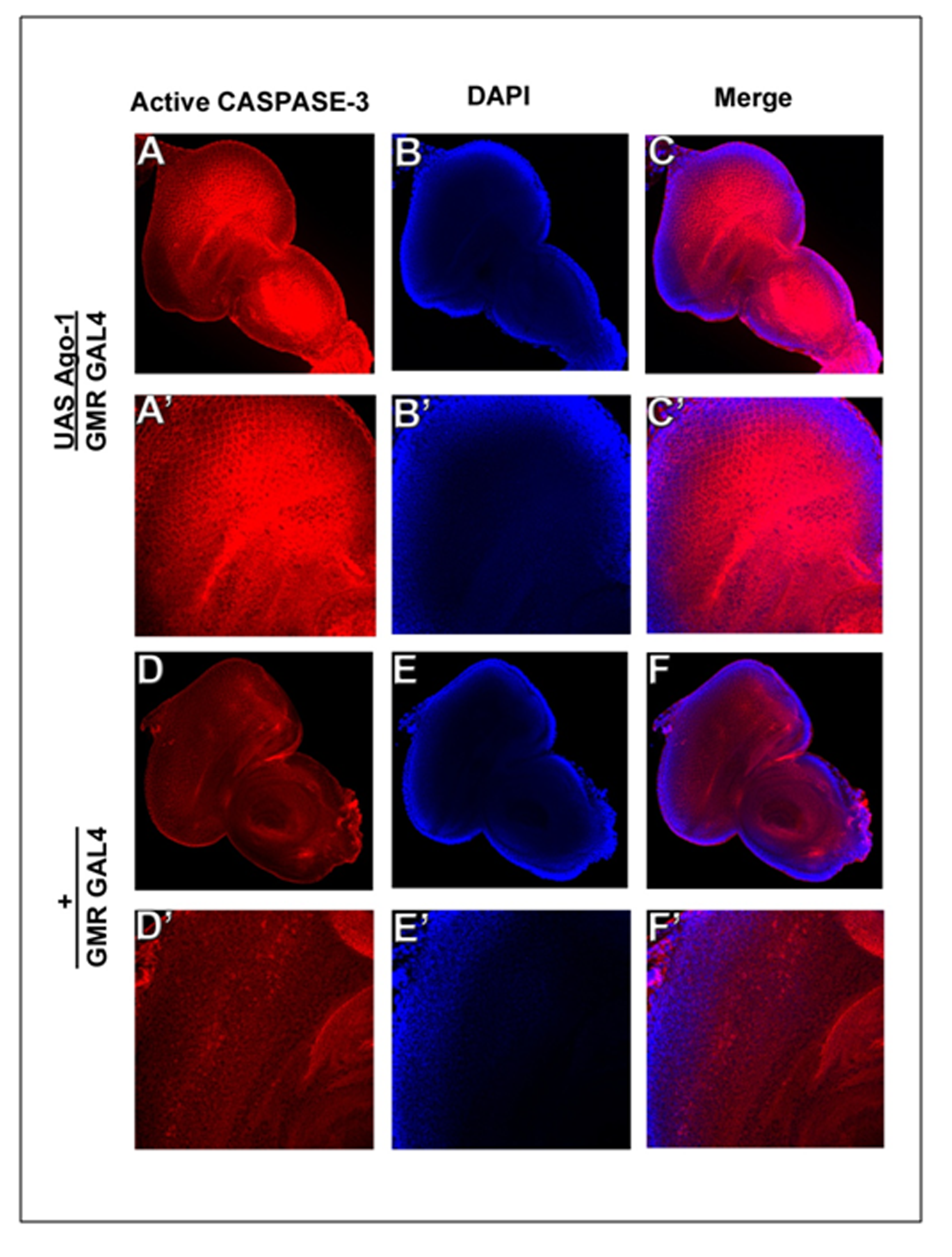

Supplement: S9 Fig — Eye discs dissected from Ago-1 over expressed (by eye specific GAL4 line- GMR GAL4) 3rd inster larvae and GMR GAL4 larve (used as a control) and probed using Human active caspase-3 antibody which actually reflects Drosophila initiator caspase, DRONC activity. Figure showing high level of DRONC activity as a result of Ago-1 over expression. (TIFF) [file pone.0190548.s009.tiff]

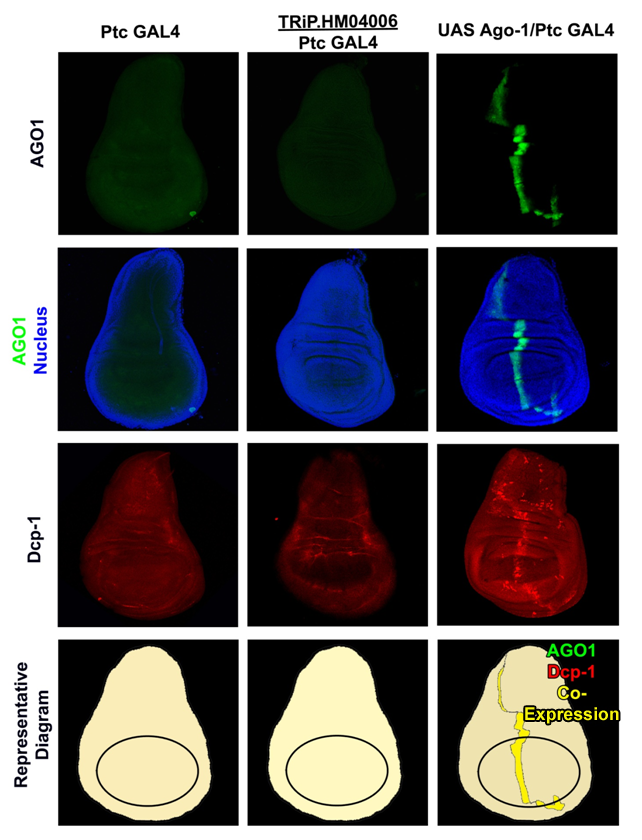

Supplement: S10 Fig — Ptc GAL4 driven Ago-1 over expression results increased AGO1 expression and Dcp-1 activation (Right panel), where as RNAi down regulation doesn’t show that pattern of expression (middle panel) compared to control (left panel). (TIF) [file pone.0190548.s010.tif]

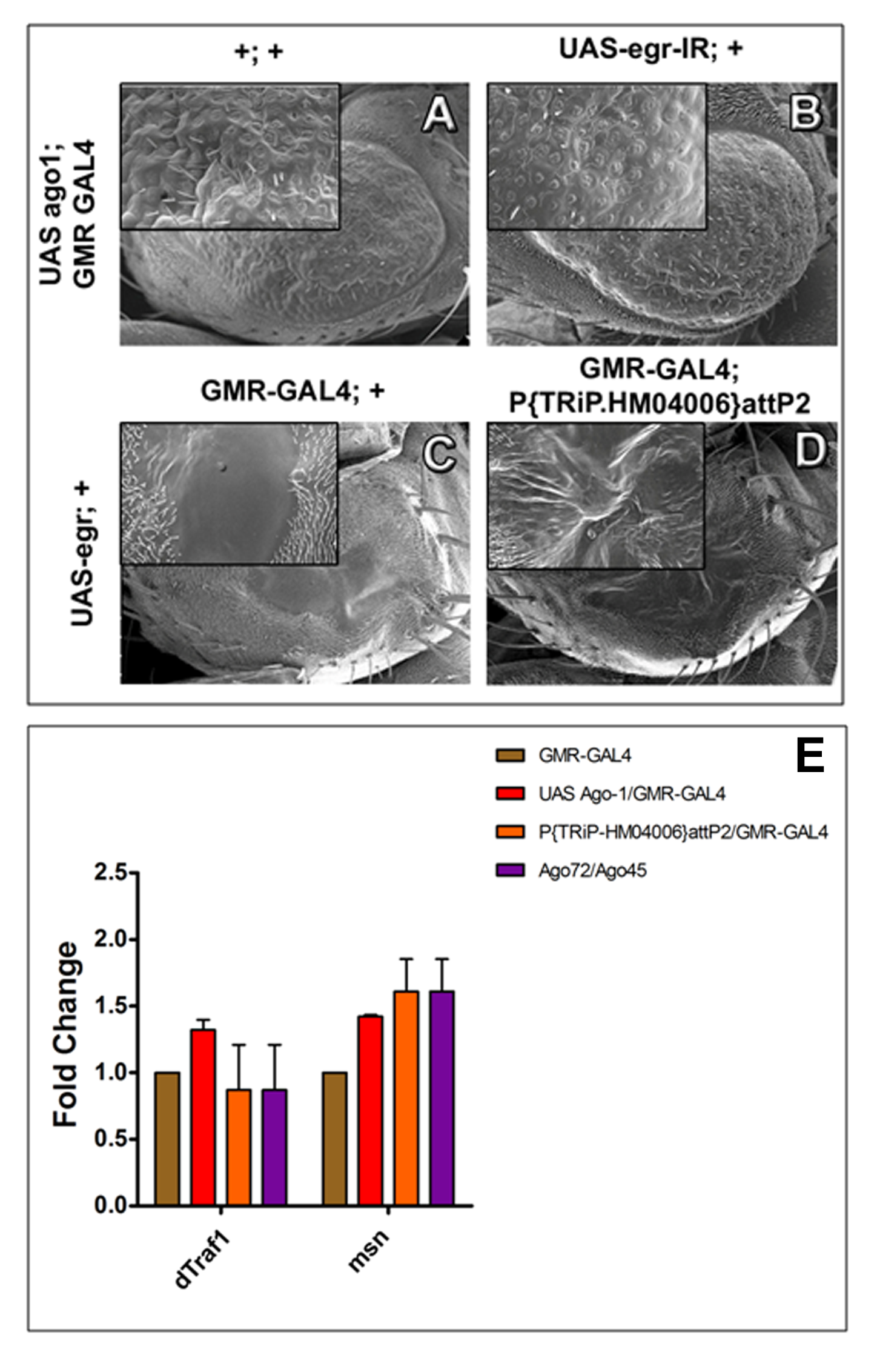

Supplement: S11 Fig — (A) Eye phenotype of Ago-1 over expressed line (B) Same with egr silenced line. (C) Eye phenotype of egr over expressed fly and (D) Ago-1 RNAi silenced flies having over expressed egr Note: Ago-1 RNAi silenced flies cannot recover over expressed egr induced small eye phenotype. (E) Real time PCR amplification graph indicates, Ago-1 over expression and mutation can not affect the expression of dTraf1 and msn. (TIFF) [file pone.0190548.s011.tiff]

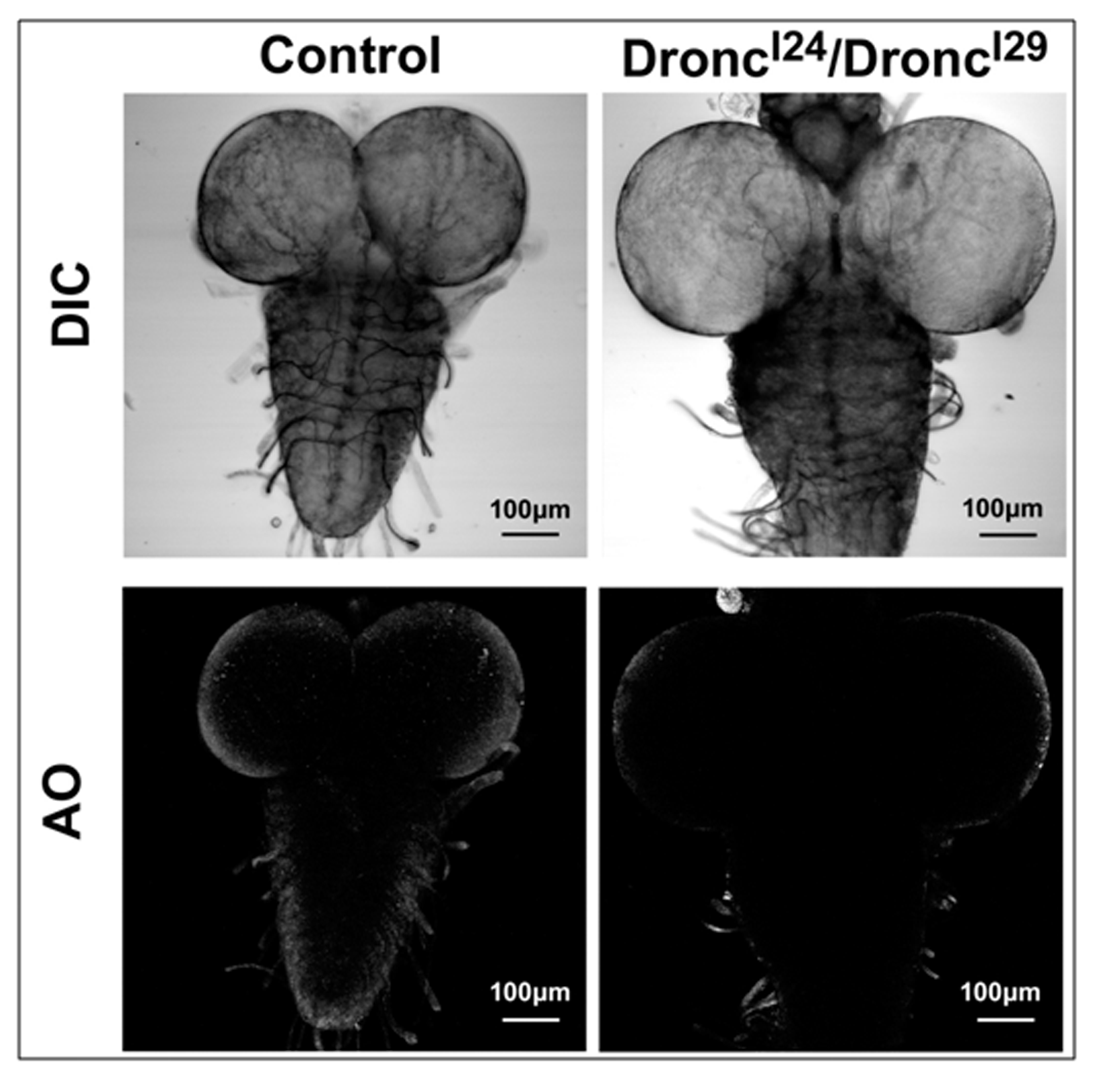

Supplement: S12 Fig — (TIFF) [file pone.0190548.s012.tiff]

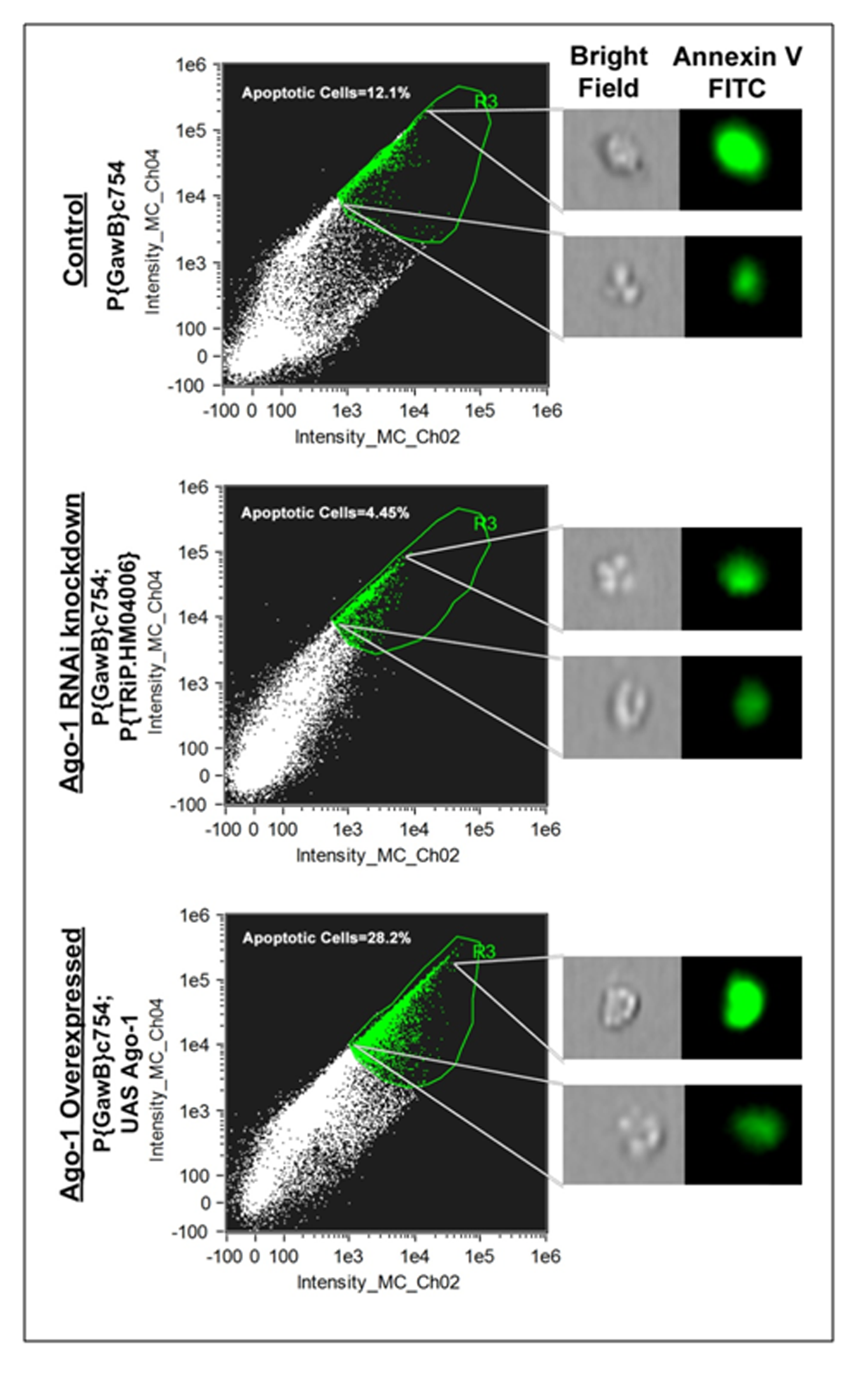

Supplement: S13 Fig — Cells from mutant line showing less apoptotic population compared to control. Right side image panels showing morphology of apoptotic cells (captured in the time of flow by 20X objective lense fitted with Amnis Flowsight). (TIFF) [file pone.0190548.s013.tiff]

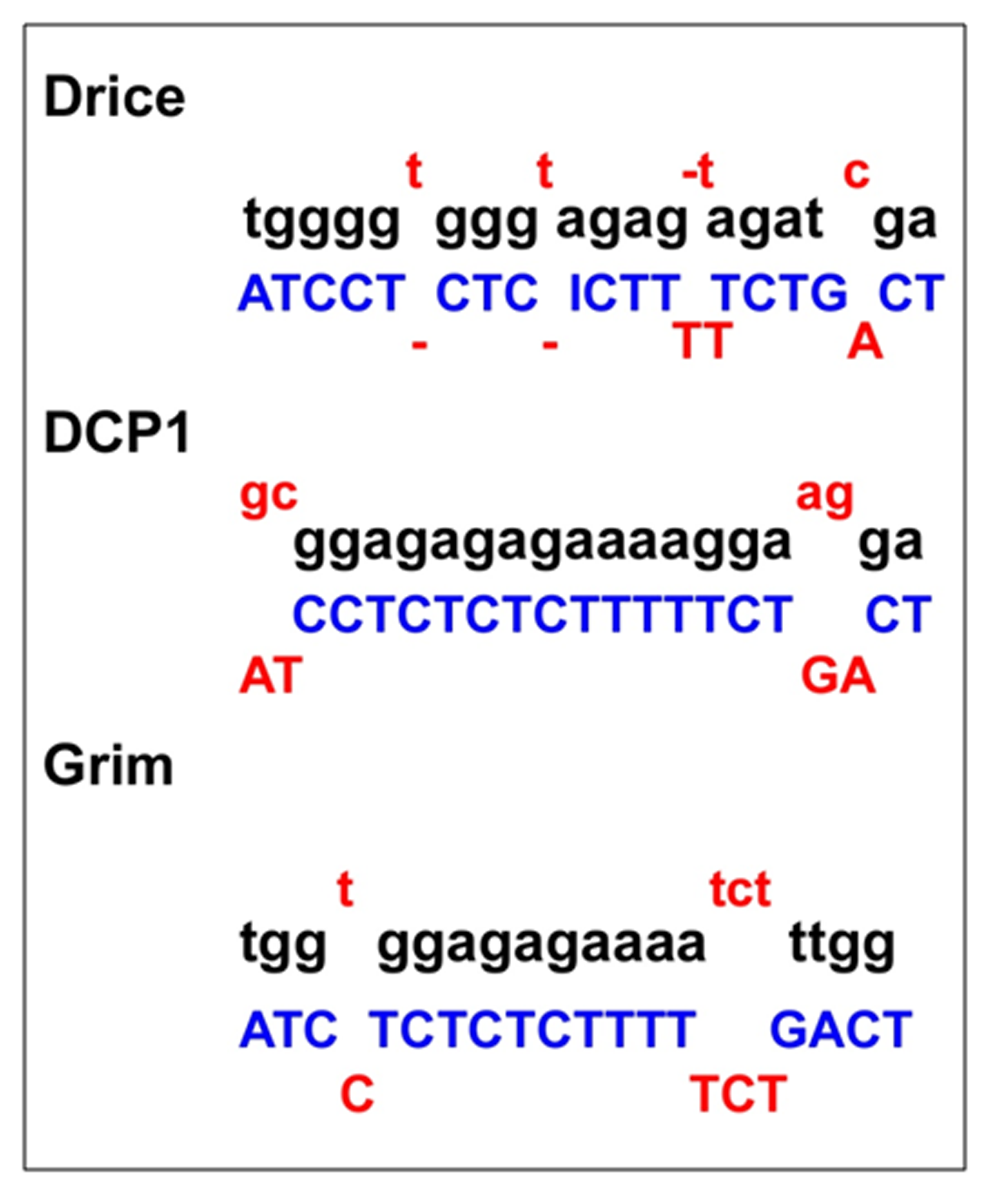

Supplement: S14 Fig — The effector caspase, Drice carries the miR-14 binding site at the 3’UTR region of its mRNA; whereas DCP1 has the binding site for the same miR at the 5’ UTR end and pro apoptotic gene, grim mRNA posses the binding location at 3’UTR region. (TIFF) [file pone.0190548.s014.tiff]

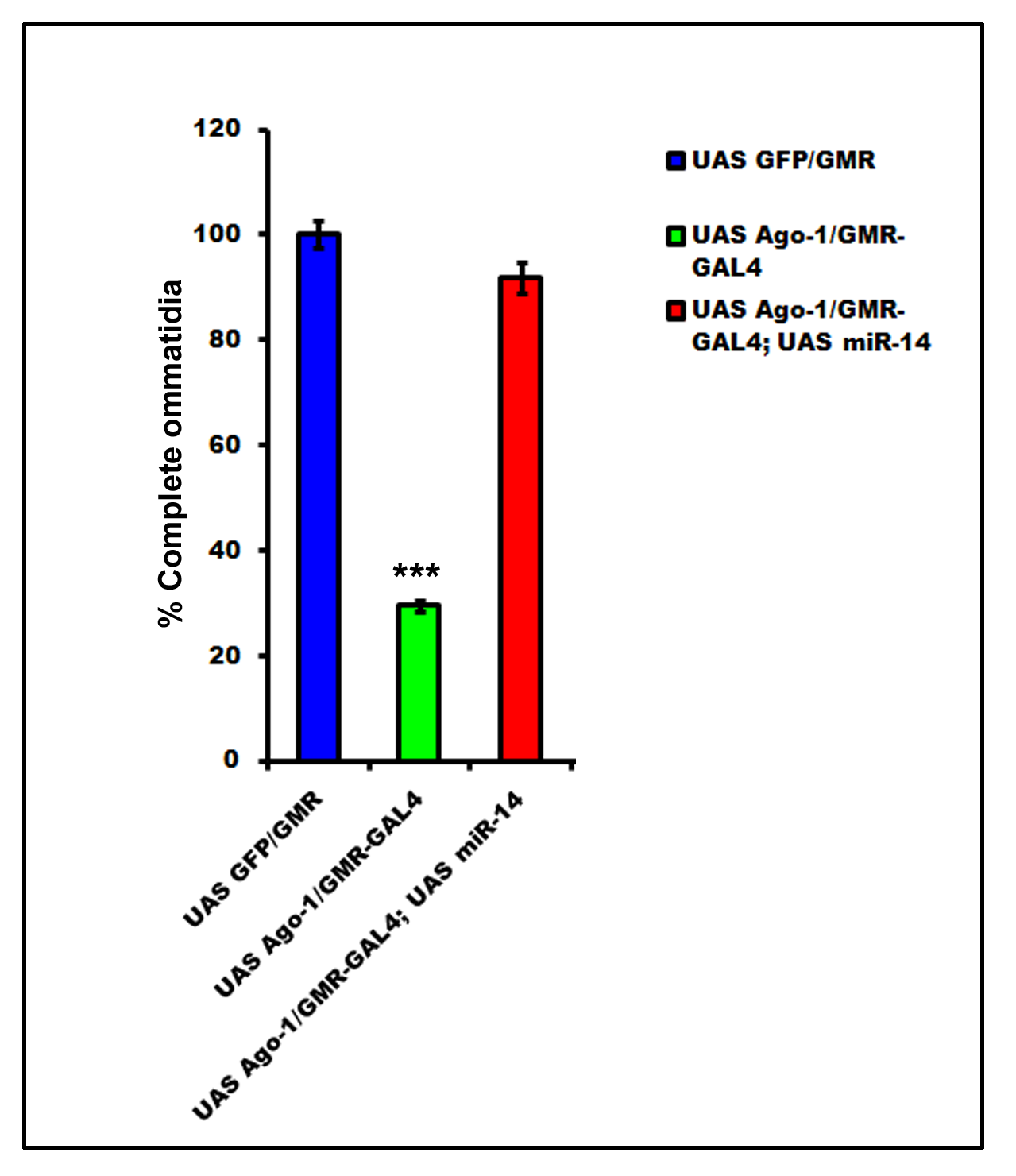

Supplement: S15 Fig — (TIFF) [file pone.0190548.s015.tiff]

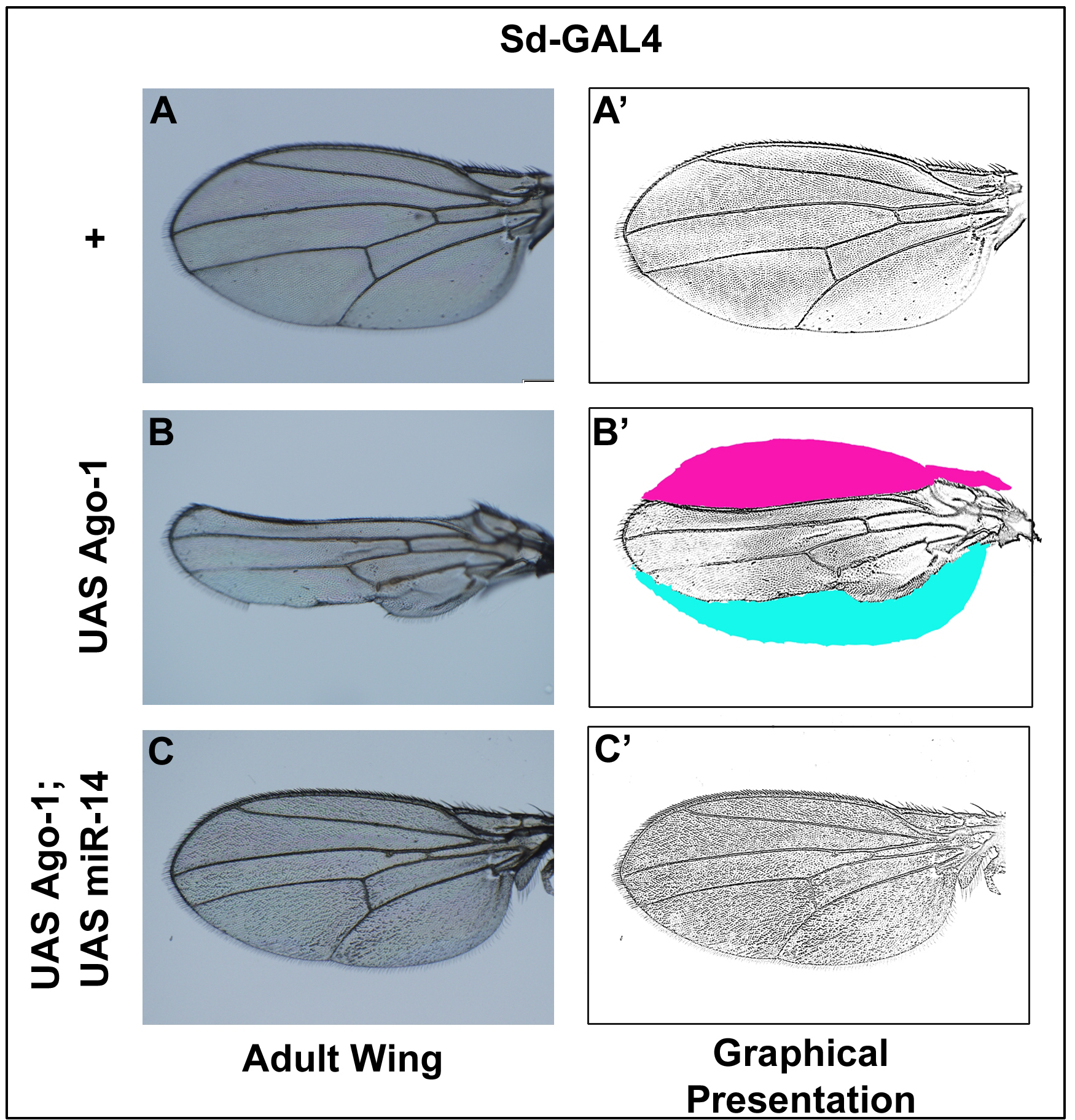

Supplement: S16 Fig — (A.) Control wing, (B) Ago-1 over expressed wing, (C) Ago-1 and miR-14 co-expressed ectopically in fly wing. (A’, B’, C’) Diagram showing the changes in the adult wing as a result of different gene expression; pink and cyan blue shaded area indicates the lost part of the normal wing as a result of Ago-1 over expression in the wing (B’). (TIFF) [file pone.0190548.s016.tiff]
