# Supplementary material for: Two way controls of apoptotic regulators consign DmArgonaute-1 a better clasp on it
Source: PLoS One. 2018 Jan 31;13(1):e0190548. doi: 10.1371/journal.pone.0190548 (PMC5791970; doi:10.1371/journal.pone.0190548)
Supplement: S1 File — (PDF) [file pone.0190548.s017.pdf]

Two way control of apoptotic regulators consign *DmArgonaute-1* a better clasp on it

Tanmoy Mondal[1,2], Indira Bag[1], Pushpavalli SNCVL [1,#], Koteswara Rao Garikapati[1], Utpal Bhadra[3] and Manika Pal Bhadra[1,2,*]

1 Department of Chemical Biology, CSIR-Indian Institute of Chemical Technology, Tarnaka, Hyderabad, Telangana State, India

2 Academy of Scientific and Innovative Research (AcSIR), CSIR-IICT Campus, Hyderabad, India

3 Gene Silencing and Functional Genomics Group, CSIR-Centre For Cellular and Molecular Biology, Uppal Road, Hyderabad, Telangana State, India

* Corresponding author

Email: manikapb@gmail.com, manika@iict.res.in (MPB)

Phone: 040-27193236

# Current Address- ARS Tandur, Dist: Ranga Reddy, Telangana State, India

Materials and Methods –

Fly stocks and genetic crosses - All flies were obtained from Bloomington Drosophila Stock Centre (BDSC) and were maintained at standard condition, grown at 24°C temperature using yeast-agar standard medium. All crosses were carried out at 18°C (except *GMR GAL4* and CyO marker related crosses). SEM (Scanning Electron Microscope) images of newly immerged flies were taken after maintaining them at 24°C for 3 days. RNAi (dsRNA) construct containing fly stocks of *ark (Dark), basket (bsk), tak1* and *ice (drice)* y^{1} *sc* v^{1}; P{TRiP.HMS00870}attP2 (BSC#33924),* y^{1} *sc** v^{1}*; P{TRiP.HMS00777}attP2 (BSC#32977),* y^{1} *sc** v^{1}*; P{TRiP.HMS00282}attP2 (BSC#33404) and* y^{1} *sc** v^{1}*; P{TRiP.HMS00398}attP2 (BSC#32403), y1 sc* v1; P{TRiP.HMS01779}attP2* (BSC#38315) are denoted as ark^{ds}, bsk^{ds}, $tak1^{ds}$, ice^{ds} and $Dcp\text{-}1^{ds}$ respectively. w^{1118} *P{UAS-bsk.DN}2 (BSC#6409),* the Dominant negative form of JNK [1] used in the study and denoted as bsk^{DN} , $dronc^{I24}$, $dronc^{I29}$ and $dronc^{2}$ are complete loss-of-function alleles. $dronc^{L32}$ is a hypomorphic allele [2], hid^{H109} is a loss-of-function allele induced by γ-rays[3], *UAS th* is the upstream activator sequence carrying allele of DIAP-1. *UAS-p35*(BSC#6298) and *UAS-GFP* was obtained from BDSC. *UAS-puc*, kind gifts from Dr N Tapon, *P{hs-Ago1}*, a gift from Dr T. Uemura. $Ago\text{-}1^{k08121}$ has been reported previously [4-6]. All genetic crosses are mentioned in S2 File.

Total RNA isolation and Real Time PCR-

Total RNA was isolated using TRIzol reagent (Invitrogen) according to the manufacturer's instructions and concentrations were determined at absorbance of 260 nm. cDNA were synthesized from total RNA (500 ng for miRNAs and 1 µg for mRNA) using Ecodry (Clonetech). Relative fold change of mRNA levels was determined by qPCR detection using primer sets mentioned in the Table-A. Primers against 18S RNA is used as an endogenous loading control. Primer sequences are presented in tabular form (Table-A).

qRT-PCR was performed using Applied Biosystem Power SYBR Green PCR Master Mix in 7900HT Fast Real-Time PCR System (Applied Biosystem, USA). Reaction mixture was prepared for a reaction volume –10µl containing following constituents: 3µl PCR-H2O, 1µl forward primer (0.5µM), 1µl reverse primer (0.5µM), 5 µl Power SYBR® Green PCR Master Mix and 1µl cDNA (50ng) as template. Following amplification protocol and quantification

procedure were carried out- Hot start (50°C for 2 min), Initial denaturation (95 °C for 10 min), denaturation (95°C for 15 sec) annealing and extension (60°C for 1 min) program repeated 40 cycles with each step under fluorescence measurement mode. Data presented as relative fold change to control by employing $2^{-\Delta\Delta CT}$, data collected from three biological triplicate experiment with each containing experimental triplicates. Error Bar indicates ±Standard Deviation.

QC Analysis for RNA for micro RNA microarray

Purity (Ratio of 260/280 and 260/230) and Concentration was assessed by NanoDrop 1000. Integrity of small RNA was assessed by small RNA assay on Agilent Bioanalyzer 2100.

Microarray: Labeling and hybridization

The miRNA labeling was performed using miRNA Complete Labeling and Hyb Kit (Agilent Technologies, Part Number: 5190-0456).The total RNA sample was diluted to 100ng/ul in nuclease free water. About 200ng of total RNA was dephosphorylated using Calf Intestinal Alkaline Phosphatase (CIP) master mix (Agilent Technologies, Part Number: 5190-0456) by incubating at 37ºC for 30 minutes. The dephosphorylated miRNA sample was denatured by adding Dimethyl Sulfoxide and heating at 100º C for 10 minutes and transferred to ice-water bath. The Ligation master mix (Agilent Technologies, Part Number: 5190-0456) containing Cyanine 3-pCp was added to the denatured miRNA sample and incubated at 16ºC for 2 hours. The Cyanine 3-pCp labeled miRNA sample was dried completely in the vacuum concentrator (Eppendorf, Concentrator Plus, Catalog Number 5305000) at 45º C for 2 hour. The dried sample was resuspended in nuclease free water and mixed with Hybridization Mix containing blocking solution (Agilent Technologies, Part Number: 5190-0456) and Hi-RPM Hybridization Buffer (Agilent Technologies, Part Number: 5190-0456) and incubated at 100º C for 5 minutes followed by snap chill on ice for 5 minutes. The samples were hybridized on the Human miRNA 8x60K Arrays. The hybridization was carried out at 55° C for 20 hours. After hybridization, the slides were washed using Gene Expression Wash Buffer1 (Agilent Technologies, Part Number 5188-5325) at room temperature for 5 minutes and Gene Expression Wash Buffer 2 (Agilent

Technologies, Part Number 5188-5326) at 37ºC for 5 minutes. The microarray slide was scanned on a G2600D scanner (Agilent Technologies)

Feature Extraction

Data extraction from Images was done using Feature Extraction software Version 11.5.1.1 of Agilent.

Microarray Data Analysis

Images were quantified using Feature Extraction Software (Version-11.5, Agilent). Feature extracted raw data was analyzed using GeneSpring GX Version 12.0 software from Agilent. Normalization of the data was done in GeneSpring GX using the 90th percentile shift (Percentile shift normalization is a global normalization, where the locations of all the spot intensities in an array are adjusted. This normalization takes each column in an experiment independently, and computes the nth percentile of the expression values for this array, across all spots (where n has a range from 0-100 and n=90 is the median). It subtracts this value from the expression value of each entity) and normalized to Specific control Samples. Significant miRNA up and down regulated in test samples with respect to control sample were identified. Statistical T-test p-value was calculated based on volcano Plot. The gene targets for the differentially regulated miRNA were identified using TargetScan database http://www.targetscan.org/ which is integrated in GeneSpring GX software. The differentially expressed miRNA were clustered using hierarchical clustering based on Pearson coefficient correlation algorithm to identify significant miRNA expression patterns across the different conditions of the experiment.

Western Blot Analysis- Western blot was carried out as mentioned previously [7]. Heads of adult flies of indicated genotypes were cut and homogenized in lysis buffer (composition). Supernatant collectedafter brief centrifugation was used for western blot analysis. Western blot was carried out using following primary antibodies- anti Tak1 (Sigma#SAB2700063), anti hep (a kind gift from Julian Ng), anti cleaved Caspase-3 antibody (Cell Signaling Technology# 966; 1:1000 dilution), Ago-1 (Abcam#ab5070, 1:1000), Anti-beta Actin (Abcam#ab8224, 1:1500), JNK (Santa Cruz Biotechnology# sc-571, 1:750), p-JNK (Santa Cruz Biotechnology# sc-6254, 1:750 and Cell Signaling Technology#9251, 1:1000), cleaved Dcp-1 (Cell Signaling

Technology#9578, 1:1000), DIAP-1 (Santa Cruz Biotechnology# sc-32414, 1:600), hid (Santa Cruz Biotechnology# sc-15767, 1:600), grim (Santa Cruz Biotechnology# sc-15763, 1:600), rpr (Santa Cruz Biotechnology# sc-15697, 1:600). Blots were developed using respective HRP conjugated secondary antibodies and were visualised by enhanced chemiluminescence. Band density of each blot was measured using ImageJ software and plotted. Every experiment was performed in triplicates and mean average was plotted.

Immuno staining- Each genotype was stained using equal antibody concentration (write the concentration) and imaged under confocal microscope Name and make of the microscope. Identical microscopic setting (Identical laser percentage, gain, HV and offset) were taken for all immune histochemistry studies to observe Ago-1 expression and localization, JNK phosphorylation site, activation of caspase, DCP-1 in *Drosophila* larval brain and eye-antennal discs. Brain and discs were dissected under chilled PBS, fixed in 4% PFA (Para Formaldehyde) solution on ice for 20 min. Rabbit anti cleaved caspase-3 antibody (Cell Signaling Technology# 9661; 1:150), Ago-1 (Abcam#5070, 1:150), p-JNK (Santa Cruz Biotechnology# sc-6254, 1:75 and Cell Signaling Technology#9251, 1:100), cleaved Dcp-1 (Cell Signaling Technology#9578, 1:100) was used as primary antibody. All the hybridization studies were performed overnight at 4ºC. Cy3 conjugated anti-rabbit and anti-mouse, FITC conjugated anti-mouse and anti-rabbit secondary antibodies were obtained from Jackson ImmunoResearch Laboratories, Inc, and used at 1:200 dilutions. After incubation with secondary antibodies for 2 hours the tissues were washed three times of 5 mins each in PBS. Tissue samples were mounted in DAPI containing mounting medium (Vectashield). Images were captured on a laser-scanning confocal microscope (OLYMPUS, FV1000) using 20X, 40X and 100X oil objective lenses. Images were compiled using Photoshop 7.0 and Photoshop CS3 (Adobe).

Acridine Orange (AO) staining- AO staining was carried out by previously mentioned method[8]. Larval brain and eye disc of indicated genotypes were dissected under chilled PBS and immediately stained with AO staining solution. Images were taken on a laser-scanning confocal microscope (OLYMPUS, FV1000) using 543nm laser with rhodamine red channel and 20X objective lens after mounting in PBS. Images were arranged using Photoshop 7.0 and Photoshop CS3 (Adobe).

TUNEL assay- Cells undergoing apoptosis in 3rd instar larval eye imaginal disc were detected by an in situ Apoptosis Detection Kit (TaKaRa#MK500) that works on the basis of terminal deoxynucleotidyl transferase-mediated dUTP nick-end labelling method (TUNEL). In this assay terminal deoxynucleotidyl transferase was used to label 3'-OH ends of fragmented DNA, generated during the process of apoptosis. Fluorescein-dUTP only labels cells that are undergoing apoptosis with high sensitivity.Labelling were carried out following manufacturer's protocol.

Chromatin immunoprecipitation (ChIP) –

Chromatin immunoprecipitation (ChIP) assay was performed after isolating chromatin from ~10,000 *UAS Ago-1/GMR GAL4* adult fly heads followed by crosslinking DNA-protein with formaldehyde. DNA was sheared to an average size of 100-150 bp using the Bioruptor sonicator set to 'high amplitude' with 30 sec ON and 30 sec OFF pulses for 14 min for a total number of 14 cycles. Chromatin was immunoprecipitated overnight at 4ºC using 8 µg of AGO1 antibody (ab5070, abcam). Following day, samples were incubated with 100 µl of 50% ProteinA Sepharose beads (GE healthcare) for 2 hrs at 4ºC. One separate set, containing ProteinA Sepharose beads along with crosslinked fragmented DNA without antibody was also incubated as negative control. Immunoprecipitated crosslinked materials were washed with low salt buffer, high salt buffer, LiCl buffer and TE buffer sequentially. Immunoprecipitated crosslinked materials were eluted with SDS containing elution buffer and reverse crosslinked with NaCl at 65ºC for 6 hours. ChIP DNA was treated with RNase A and Proteinase K followed by purification using PCR purification kit (MN-NucleoSpin® Gel and PCR Clean-up # 740609.10 / .50 / .250). Target amplification was carried out using region-specific primer sets (Table-B). All reactions were prepared in 50 µl volumes containing 300 ng chromatin DNA along with 25 µl EmeraldAmp GT PCR Master Mix (Clontech# RR310Q), and amplified for 35 cycles according to manufacturer's instruction. 5% crosslinked fragmented DNA was used as an input and the sample from "no antibody beads" was used as negative control. Gel image was taken after running samples at 80V current in 1% agarose gel.

Preparation of nuclear extracts from *Drosophila* heads -

Freshly dissected heads of *UAS Ago-1/GMR GAL4* adult flies were used for preparation of nuclear extract. Heads ware washed off under running 0.7% sodium chloride solution. Then heads were further washed with PBS. Finally, the solution which contains heads was filtered to form a hard cake of heads. The weight of the head cake was recorded.

The head cake was suspended in 1ml of NU-I buffer per gram of head. The heads were homogenized with mortor-driven Teflon pestle at 2500rpm at 4°C. The homogenate was passed through a single layer of mira cloth supported by a funnel over a GSA centrifugation tube. The mira cloth was washed with 2 ml of NU-I buffer per gram of heads. 3ml of NU-I buffer per gram of head was added directly to the filtrate. The filtrate was centrifuged at 8000 rpm for 15 min at 40C. The cytoplasm was collected into fresh tube. The nuclei were resuspended in 1ml of NU-II buffer per gram of head and 1/10th volume of 4M ammonium sulphate was added and mixed gently by rotation in cold room for 20 min. The contents were subjected to ultra centrifugation for 1h using Ti-60 rotor at 35,000rpm. The supernatant was removed carefully leaving behind the bulk of lipid layer. The nuclear extract of head was passed through the desalting column and fractions were collected in HEMG150 buffer. Those fractions having low salt and high amount of protein was aliquot and stored in –80ºC (all buffer compositions are mentioned in Table-C).

Immuno Precipitation -

50-100µl of proteinA sepharose beads were taken in two eppendorf tubes washed and equilibrated with HEMG150 to form 50% slurry. Equal amount (20µg) of IgG and RNA pol II antibody (abcam# ab817) were added to the tubes separately and incubated at 40C for 4h to allow the binding. The contents were centrifuged at 2000rpm at 4°C for 30 sec and the beads were collected discarding the supernatant. Equal amount of head nuclear extract (300µg) was added and incubated overnight at 40C. The beads were washed twice each with 1ml of HEMG150 and HEMG250 buffers and the bound proteins were eluted with 100µl SDS sample buffer followed by heating at 95°C for 5 minutes. The eluted proteins were run on SDS-PAGE along with 5% input sample and western blot was carried out using RNA pol II (abcam# ab817) and AGO1(abcam# ab5070) antibody.

Annexin V-FITC Apoptosis Detection-

Third instar larval brains from *UAS Ago-1/ P{GawB}c754* (*Ago-1* over expressed in brain), *P{TRiP.HM04006}attP2 / P{GawB}c754* (*Ago-1* RNAi in brain) and *P{GawB}c754* (Control) were dissected under Schneider's media and immediately transferred to a tube containing ice-cold PBS. After getting 40–45 brains, they were washed twice with 1 ml PBS. 500 µl of Trypsine-EDTA was added to each tube in a final concentration of 3 mg/ml. Brain cells were dissociated at 37°C in a Thermomixer : 500 rpm for 2.5 hour. Dissociated cells were passed through 100 µm filter. Annexin V-FITC Apoptosis Detection staining was performed using ApoAlert™ Annexin V-FITC Apoptosis Kit (Clontech# 630109) according to manufacturer's instruction and stained cells were detected using FACS (Amnis Flowsight) and data analysis was carried out using IDEAS 6.2 software. In the time of analysis apoptotic cells were identified through FITC signal and nuclear morphology of cells (Amnis Flowsight has imaging facility also). FITC positive cells along with condensed and fragmented nuclei were considered as apoptotic cells, and the area containing this kind of cell population was marked with green line in the histogram.

Table-A	
**Primer Name**	**Primer Sequence**
egr- FP	TCGATAATCTCCAGCAGCGT
egr-RP	CGCCAACATCATCCACAGAG
bsk-FP	ATGGACGCTAATCTCTGCCA
bsk-RP	GTACGTGCCAGACCGAAATC
hep-FP	CATTGATCACGGATGCAGCA
hep-RP	TTCTGTGGGGAATTGCTTGC
puc-FP	TCCACCAGCTCATCCGTATC
puc-RP	CCCTCGTCAAATTGCTAGCC
kay-FP	CATTGATGTCCTGGGCATGG
kay-RP	TGGGCACTTGAAGTATCGGT
Jra-FP	ATCAACTCACCGGATCTGTC
Jra-RP	TGCCTGGGAGTTAGTGTGAAG
dAgo-1-FP	CAATTTGGAGGAAGCTCTCG
dAgo-1-RP	TGAGCATCATCTTCCACTGC
dTak-1-FP	GAACAAGACATCGCCACAGA
dTak-1-RP	GGCAATCGATGGTTTCTTGT
Dark-FP	TGATGACAAAGGCTTGCTTG

Dark-RP	ACTCCGTCCACATAGCCAAC
Dcp-1-FP	GGAACGATATGCAAGCGAAT
Dcp-1-RP	TCAACTTGCAGTCCTTGTGC
dmHid-FP	TTCCTGCCCTCTTTCTTTG
dmHid-RP	GTCCTTATCCGCTTCCTTCC
dTRAF1-FP	CGCACGTCTCCGTCTACATA
dTRAF1-RP	CCGAATCCTAGCTGATCTGG
dm_msn-FP	TTGGTCGATCTCACCATTGA
dm_msn-RP	ATGCCATTTGAGTTGGGAAG
Dronc-FP	GAGATTGGAATGCCGAAGAG
Dronc-RP	GCTGGTTCTCCGTTCGTTTA
Grim-FP	GGGAAGTCAACAGGGATCGA
Grim-RP	CGTCGTCCTCATCGTTGTTC
Rpr-FP	AGTGGCATTCTACATACCCGA
Rpr-RP	CTTGCGATATTTGCCGGACT
Ice-FP	AATCTGACTCGGGTGCTCAA
Ice-RP	TGTGTCCTTGGCGTAGATGT
Diap-1-FP	TCAGAGGAAGAGCAGCAGAC
Diap-1-RP	TTGCACAACTTTTCCTCGGG

Table-B

Set No	Primer Name	Primer Sequence	Product Size	Predicted Promoter Sequence
**1**	**Forward Primer1**	TAAAAAGACGGCTCGTTGCT	**98bp**	**ATCGTCTGAA**
	**Reverse Primer1**	TGCACACAGCACCACACTTA		
**2**	**Forward Primer2**	AGTCAGTTGGTTGGAAATTGG	**100bp**	**AGTCAGTTGG**
	**Reverse Primer2**	TCACAACCGTAACATCAGCA		
**3**	**Forward Primer3**	TTCGGACATTATGCAAACCA	**134bp**	**TGCAAACCAG**
	**Reverse Primer3**	GCCCAGCTTTTTGTTCGTAA		

Preparation of nuclear extracts from *Drosophila* heads

**Table-C**
**Composition of Buffers-**
1. NU-I buffer [15mM Hepes-KOH pH 7.6, 10mM KCl, 5mM $MgCl_2$, 0.1mM EDTA pH 8.0, 0.5mM EGTA P^H 8.0, 350mM sucrose]
2. NU-II [15mM Hepes-KOH P^H 7.6, 110mM KCl, 5mM $MgCl_2$, 0.1mM EDTA P^H 8.0]
3. HEMG 0 [25mM Hepes-KOH P^H 7.6, 0mM KCl, 12.5mM $MgCl_2$, 0.1mM EDTA P^H 8.0, 10% Glycerol]
4. HEMG1000= HEMG0 + 1000mM KCl
5. HEMG250= HEMG0 + 250mM KCl
6. 4M Ammonium Sulphate
7. 100mM PMSF
8. Complete EDTA-free protease inhibitor tablets
9. 1M DTT
10. Desalting column

Supplemental References

1. Biteau B, Jasper H. EGF signaling regulates the proliferation of intestinal stem cells in Drosophila. Development. 2011;138(6):1045-55. Epub 2011/02/11. doi: 10.1242/dev.056671

dev.056671 [pii]. PubMed PMID: 21307097; PubMed Central PMCID: PMC3042864.
2. Xu D, Li Y, Arcaro M, Lackey M, Bergmann A. The CARD-carrying caspase Dronc is essential for most, but not all, developmental cell death in Drosophila. Development. 2005;132(9):2125-34. Epub 2005/04/01. doi: dev.01790 [pii]

10.1242/dev.01790. PubMed PMID: 15800001; PubMed Central PMCID: PMC2519871.
3. Abbott MK, Lengyel JA. Embryonic head involution and rotation of male terminalia require the Drosophila locus head involution defective. Genetics. 1991;129(3):783-9. Epub 1991/11/01. PubMed PMID: 1752422; PubMed Central PMCID: PMC1204746.
4. Jin P, Zarnescu DC, Ceman S, Nakamoto M, Mowrey J, Jongens TA, et al. Biochemical and genetic interaction between the fragile X mental retardation protein and the microRNA pathway. Nat Neurosci. 2004;7(2):113-7. doi: 10.1038/nn1174. PubMed PMID: 14703574.
5. Kataoka Y, Takeichi M, Uemura T. Developmental roles and molecular characterization of a Drosophila homologue of Arabidopsis Argonaute1, the founder of a novel gene superfamily. Genes to cells : devoted to molecular & cellular mechanisms. 2001;6(4):313-25. PubMed PMID: 11318874.
6. Williams RW, Rubin GM. ARGONAUTE1 is required for efficient RNA interference in Drosophila embryos. Proceedings of the National Academy of Sciences of the United States of America. 2002;99(10):6889-94. doi: 10.1073/pnas.072190799. PubMed PMID: 12011447; PubMed Central PMCID: PMC124499.
7. Bhadra U, Mondal T, Bag I, Mukhopadhyay D, Das P, Parida BB, et al. HDAC inhibitor misprocesses bantam oncomiRNA, but stimulates hid induced apoptotic pathway. Scientific reports. 2015;5:14747. doi: 10.1038/srep14747. PubMed PMID: 26442596; PubMed Central PMCID: PMC4595805.
8. Mondal T, Lavanya AVS, Mallick A, Dadmala TL, Kumbhare RM, Bhadra U, et al. Novel Triazole linked 2-phenyl benzoxazole derivatives induce apoptosis by inhibiting miR-2, miR-13 and miR-14 function in Drosophila melanogaster. Apoptosis : an international journal on programmed cell death. 2017;22(6):786-99. doi: 10.1007/s10495-017-1367-1. PubMed PMID: 28401354.
