# Supplementary material for: Two way controls of apoptotic regulators consign DmArgonaute-1 a better clasp on it
Source: PLoS One. 2018 Jan 31;13(1):e0190548. doi: 10.1371/journal.pone.0190548 (PMC5791970; doi:10.1371/journal.pone.0190548)
Supplement: S2 File — (PDF) [file pone.0190548.s018.pdf]

Two way control of apoptotic regulators consign *DmArgonaute-1* a better clasp on it

Tanmoy Mondal[1,2], Indira Bag[1], SNCVL Pushpavalli[1,#], Koteswara Rao Garikapati[1], Utpal Bhadra[3] and Manika Pal Bhadra[1,2,*]

1 Department of Chemical Biology, CSIR-Indian Institute of Chemical Technology, Tarnaka, Hyderabad, Telangana State, India

2 Academy of Scientific and Innovative Research (AcSIR), CSIR-IICT Campus, Hyderabad, India

3 Gene Silencing and Functional Genomics Group, CSIR-Centre For Cellular and Molecular Biology, Uppal Road, Hyderabad, Telangana State, India

* Corresponding author
Email: manikapb@gmail.com, manika@iict.res.in (MPB)
Phone: 040-27193236

# Current Address- ARS Tandur, Dist: Ranga Reddy, Telangana State

Materials and Methods

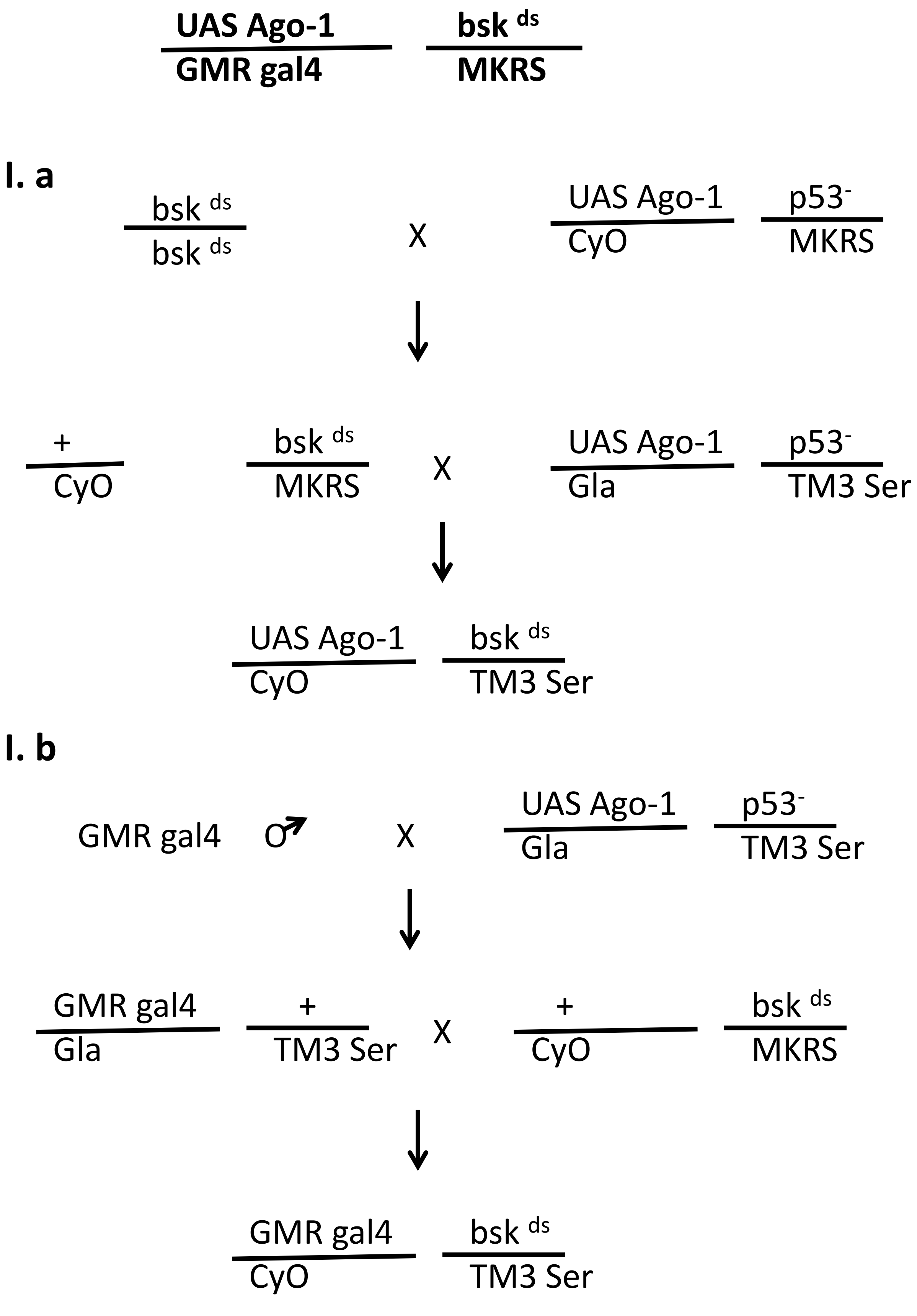
UAS Ago-1 / GMR gal4
bsk ds / MKRS
I. a
bsk ds / bsk ds
X
UAS Ago-1 / CyO
p53- / MKRS
+ / CyO
bsk ds / MKRS
X
UAS Ago-1 / Gla
p53- / TM3 Ser
UAS Ago-1 / CyO
bsk ds / TM3 Ser
I. b
GMR gal4 ♂
X
UAS Ago-1 / Gla
p53- / TM3 Ser
GMR gal4 / Gla
+ / TM3 Ser
X
+ / CyO
bsk ds / MKRS
GMR gal4 / CyO
bsk ds / TM3 Ser

II.

$$\frac{\text{UAS Ago-1}}{\text{CyO}} \; \frac{\text{p53}^{-}}{\text{MKRS}} \quad \text{X} \quad \frac{\text{GMR gal4}}{\text{CyO}} \; \frac{\text{bsk}^{\text{ds}}}{\text{TM3 Ser}}$$

↓

$$\frac{\text{UAS Ago-1}}{\text{GMR gal4}} \; \frac{\text{bsk}^{\text{ds}}}{\text{MKRS}}$$

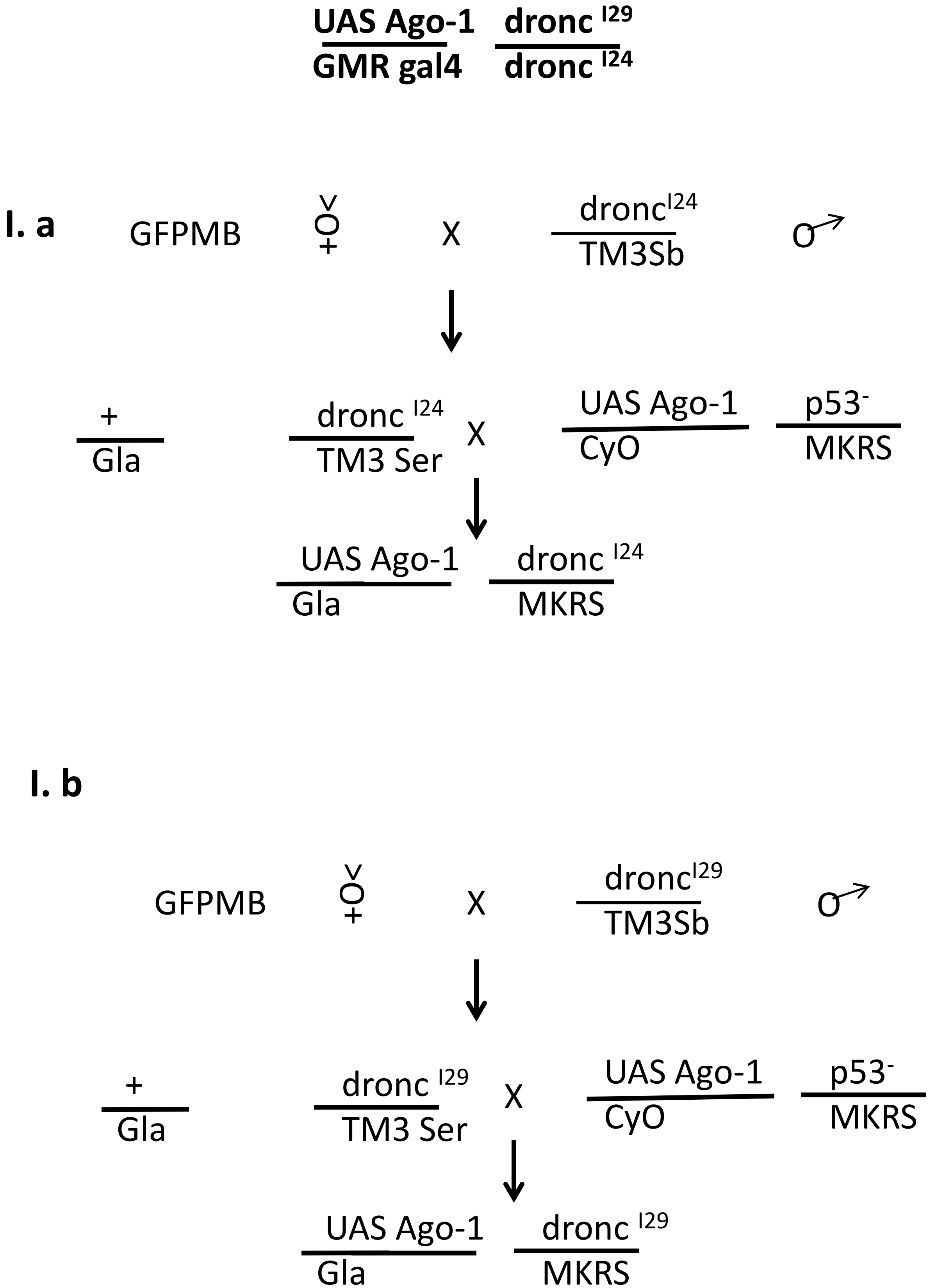
UAS Ago-1
GMR gal4
dronc I29
dronc I24
I. a
GFPMB ♀
X
dronc I24
TM3Sb
♂
+
Gla
dronc I24
TM3 Ser
X
UAS Ago-1
CyO
p53-
MKRS
UAS Ago-1
Gla
dronc I24
MKRS
I. b
GFPMB ♀
X
dronc I29
TM3Sb
♂
+
Gla
dronc I29
TM3 Ser
X
UAS Ago-1
CyO
p53-
MKRS
UAS Ago-1
Gla
dronc I29
MKRS

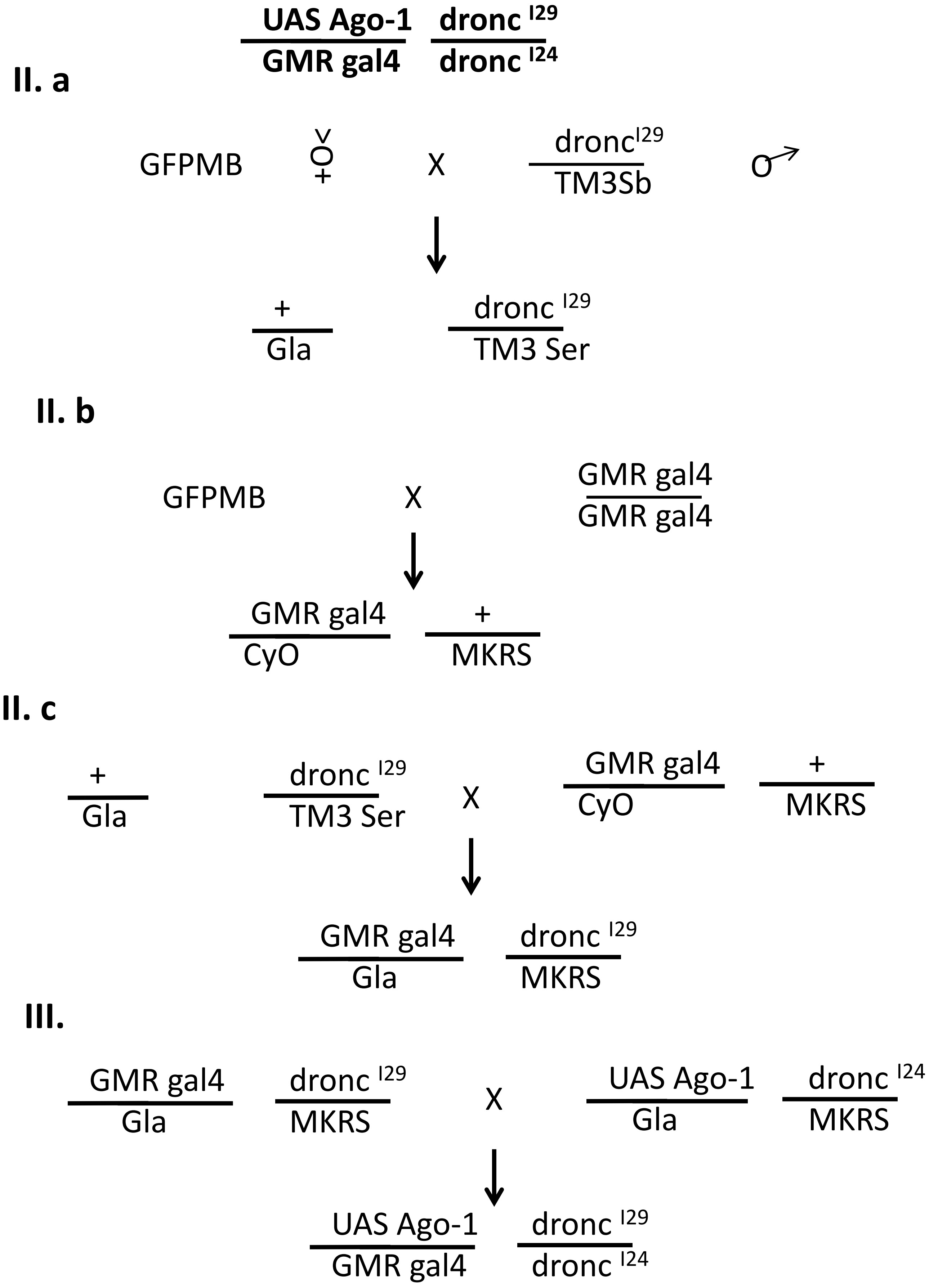
UAS Ago-1 / GMR gal4   dronc I29 / dronc I24
II. a
GFPMB ♀ X dronc I29 / TM3Sb ♂
+ / Gla   dronc I29 / TM3 Ser
II. b
GFPMB X GMR gal4 / GMR gal4
GMR gal4 / CyO   + / MKRS
II. c
+ / Gla   dronc I29 / TM3 Ser X GMR gal4 / CyO   + / MKRS
GMR gal4 / Gla   dronc I29 / MKRS
III.
GMR gal4 / Gla   dronc I29 / MKRS X UAS Ago-1 / Gla   dronc I24 / MKRS
UAS Ago-1 / GMR gal4   dronc I29 / dronc I24

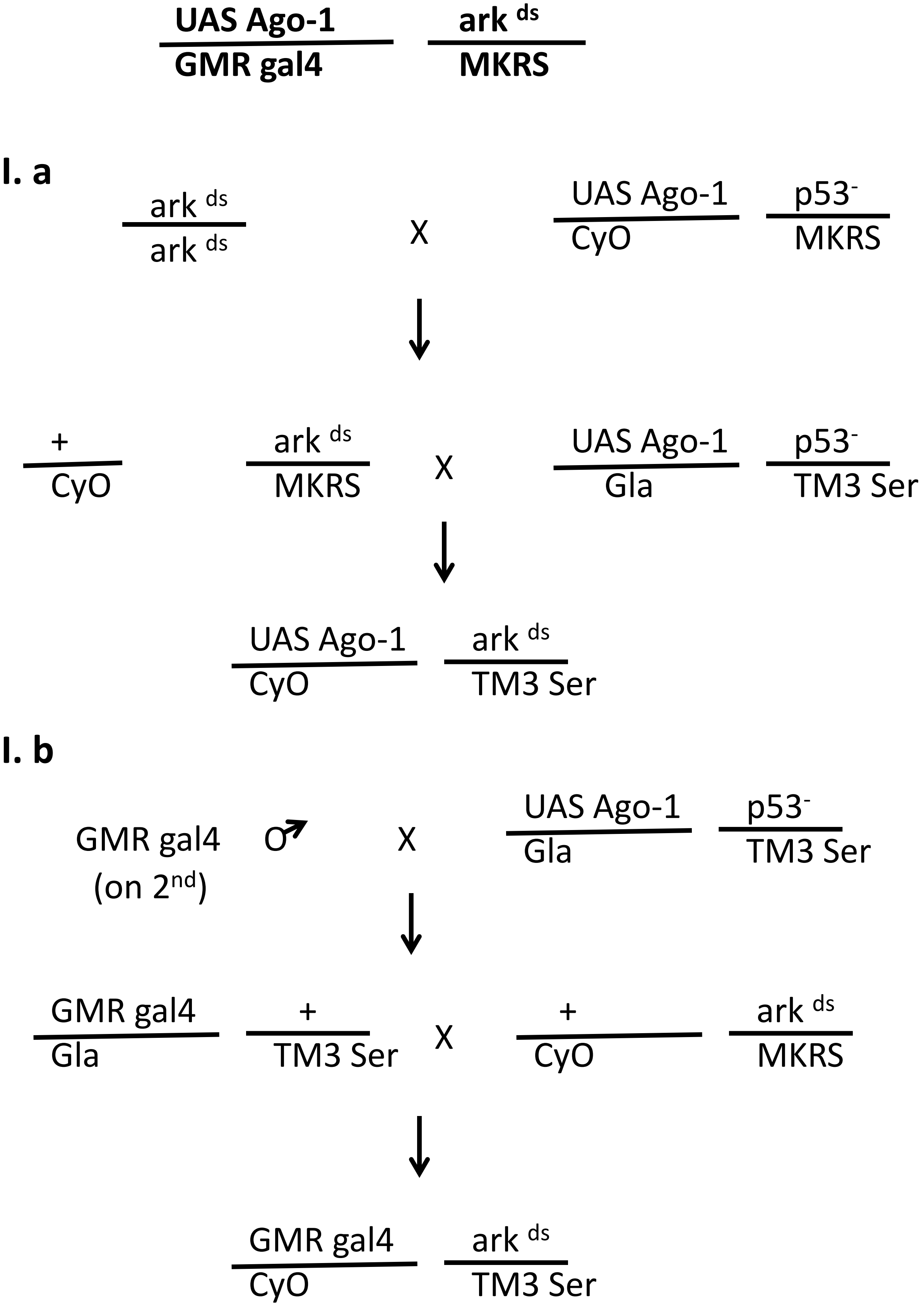
UAS Ago-1
GMR gal4
ark ds
MKRS
I. a
ark ds
ark ds
X
UAS Ago-1
CyO
p53-
MKRS
+
CyO
ark ds
MKRS
X
UAS Ago-1
Gla
p53-
TM3 Ser
UAS Ago-1
CyO
ark ds
TM3 Ser
I. b
GMR gal4 ♂
(on 2nd)
X
UAS Ago-1
Gla
p53-
TM3 Ser
GMR gal4
Gla
+
TM3 Ser
X
+
CyO
ark ds
MKRS
GMR gal4
CyO
ark ds
TM3 Ser

II.

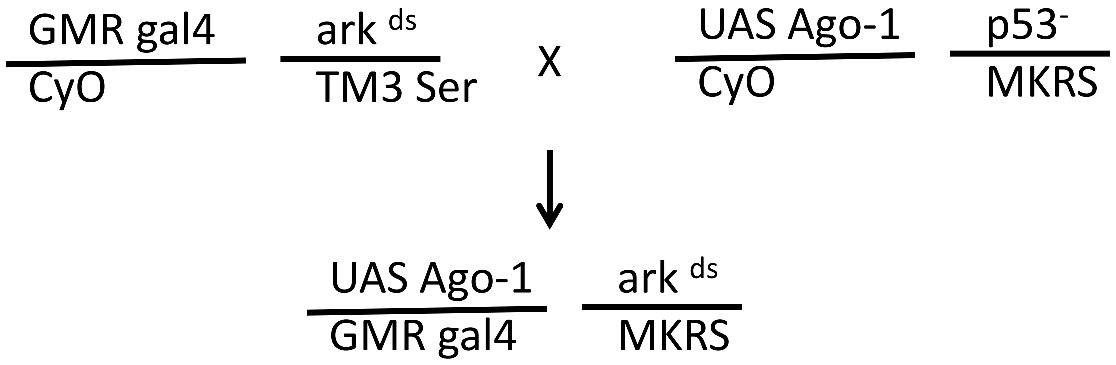
GMR gal4
CyO
ark ds
TM3 Ser
X
UAS Ago-1
CyO
p53-
MKRS
UAS Ago-1
GMR gal4
ark ds
MKRS

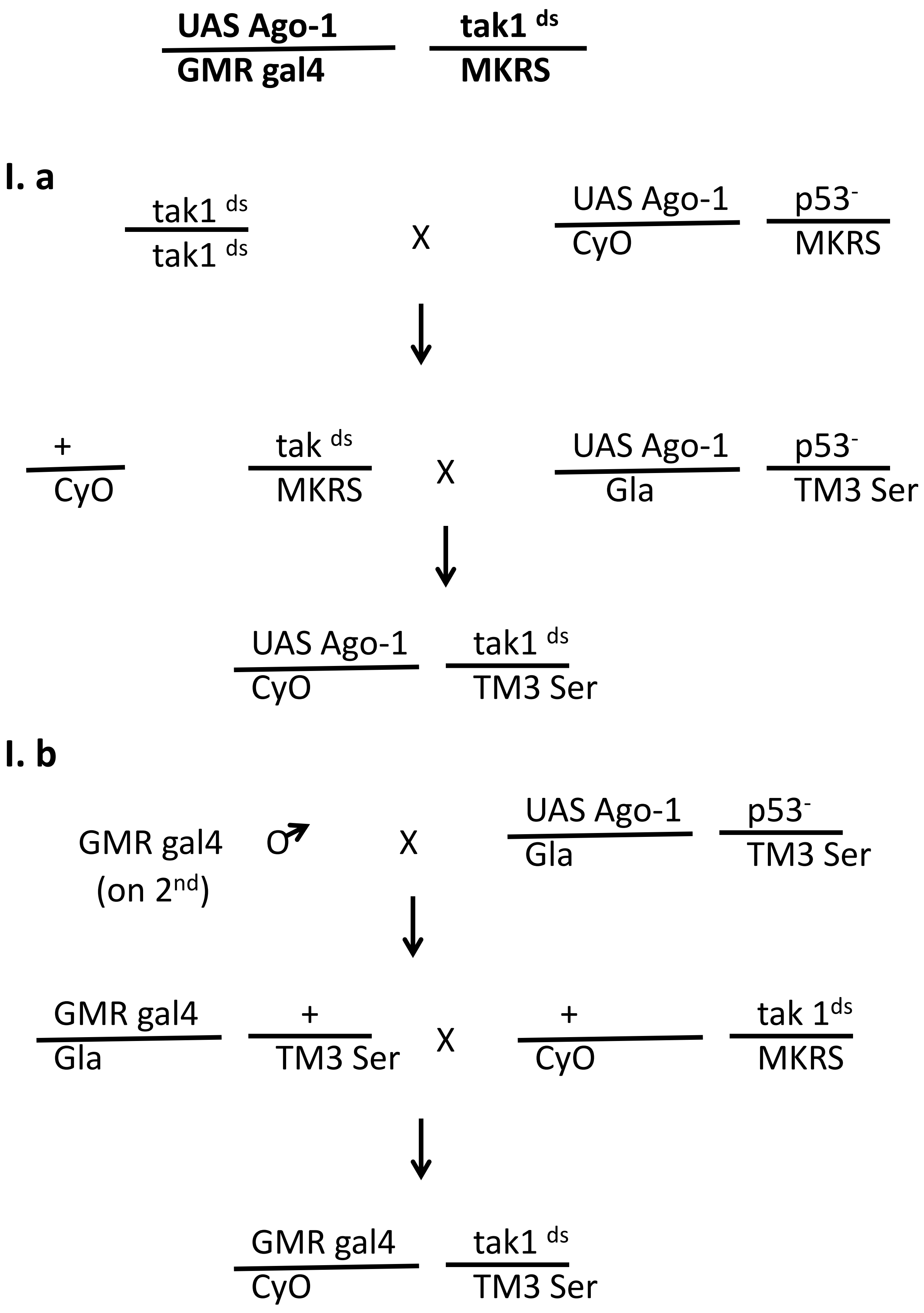
UAS Ago-1 / GMR gal4
tak1 ds / MKRS
I. a
tak1 ds / tak1 ds
X
UAS Ago-1 / CyO
p53- / MKRS
+ / CyO
tak ds / MKRS
X
UAS Ago-1 / Gla
p53- / TM3 Ser
UAS Ago-1 / CyO
tak1 ds / TM3 Ser
I. b
GMR gal4 (on 2nd) ♂
X
UAS Ago-1 / Gla
p53- / TM3 Ser
GMR gal4 / Gla
+ / TM3 Ser
X
+ / CyO
tak 1ds / MKRS
GMR gal4 / CyO
tak1 ds / TM3 Ser

II.

$$\frac{\text{GMR gal4}}{\text{CyO}} \;\; \frac{\text{tak1}^{\text{ds}}}{\text{TM3 Ser}} \quad \text{X} \quad \frac{\text{UAS Ago-1}}{\text{CyO}} \;\; \frac{\text{p53}^{-}}{\text{MKRS}}$$

$$\downarrow$$

$$\frac{\text{UAS Ago-1}}{\text{GMR gal4}} \;\; \frac{\text{tak1}^{\text{ds}}}{\text{MKRS}}$$

$$\frac{\textbf{UAS Ago-1}}{\textbf{GMR gal4}} \quad \frac{\textbf{ice}^{\textbf{ds}}}{\textbf{MKRS}}$$

I. a

BSC#32403 $\frac{\text{ice}^{ds}}{\text{ice}^{ds}}$ X $\frac{\text{UAS Ago-1}}{\text{CyO}} \quad \frac{\text{p53}^{-}}{\text{MKRS}}$

↓

$$\frac{+}{\text{CyO}} \quad \frac{\text{ice}^{ds}}{\text{MKRS}}$$

I. b

GMR gal4 (on 2nd) ♂ X $\frac{\text{UAS Ago-1}}{\text{Gla}} \quad \frac{\text{p53}^{-}}{\text{TM3 Ser}}$

↓

$\frac{\text{GMR gal4}}{\text{Gla}} \quad \frac{+}{\text{TM3 Ser}}$ X $\frac{+}{\text{CyO}} \quad \frac{\text{ice}^{ds}}{\text{MKRS}}$

↓

$$\frac{\text{GMR gal4}}{\text{CyO}} \quad \frac{\text{ice}^{ds}}{\text{TM3 Ser}}$$

II.

$$\frac{\text{GMR gal4}}{\text{CyO}} \; \frac{\text{ice}^{\text{ds}}}{\text{TM3 Ser}} \quad \text{X} \quad \frac{\text{UAS Ago-1}}{\text{CyO}} \; \frac{\text{p53}^{-}}{\text{MKRS}}$$

$$\downarrow$$

$$\frac{\text{UAS Ago-1}}{\text{GMR gal4}} \; \frac{\text{ice}^{\text{ds}}}{\text{MKRS}}$$

$\frac{\text{UAS Ago-1}}{\text{GMR gal4}}$ $\frac{\text{ice}^{ds}}{\text{ice}^{ds}}$

I. a

$\frac{\text{ice}^{ds}}{\text{ice}^{ds}}$ X $\frac{\text{UAS Ago-1}}{\text{CyO}}$ $\frac{\text{p53}^{-}}{\text{MKRS}}$

↓

$\frac{+}{\text{CyO}}$ $\frac{\text{ice}^{ds}}{\text{MKRS}}$ X $\frac{\text{UAS Ago-1}}{\text{Gla}}$ $\frac{\text{p53}^{-}}{\text{TM3 Ser}}$

↓

$\frac{\text{UAS Ago-1}}{\text{CyO}}$ $\frac{\text{ice}^{ds}}{\text{TM3 Ser}}$

I. b

GMR gal4 (on 2nd) ♂ X $\frac{\text{UAS Ago-1}}{\text{Gla}}$ $\frac{\text{p53}^{-}}{\text{TM3 Ser}}$

↓

$\frac{\text{GMR gal4}}{\text{Gla}}$ $\frac{+}{\text{TM3 Ser}}$ X $\frac{+}{\text{CyO}}$ $\frac{\text{ice}^{ds}}{\text{MKRS}}$

↓

$\frac{\text{GMR gal4}}{\text{CyO}}$ $\frac{\text{ice}^{ds}}{\text{TM3 Ser}}$

II.

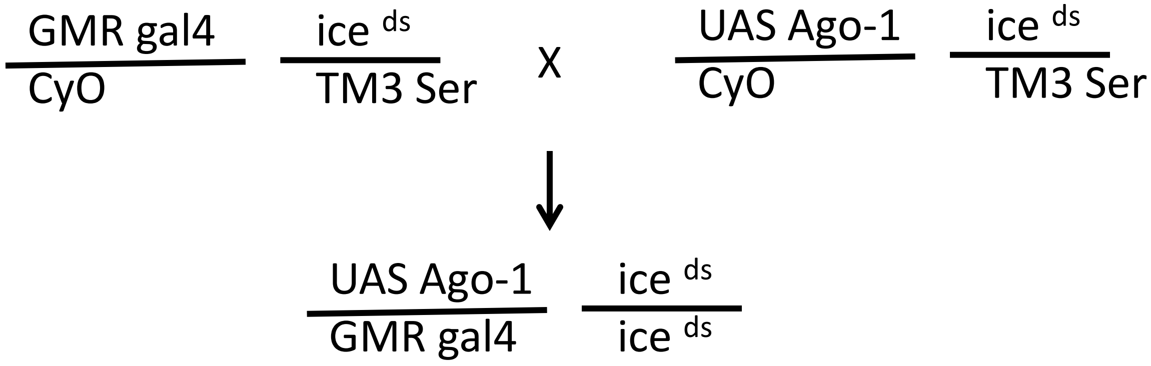
GMR gal4
CyO
ice ds
TM3 Ser
X
UAS Ago-1
CyO
ice ds
TM3 Ser
UAS Ago-1
GMR gal4
ice ds
ice ds

UAS Ago-1 / GMR gal4 ; th1 / MKRS

I.

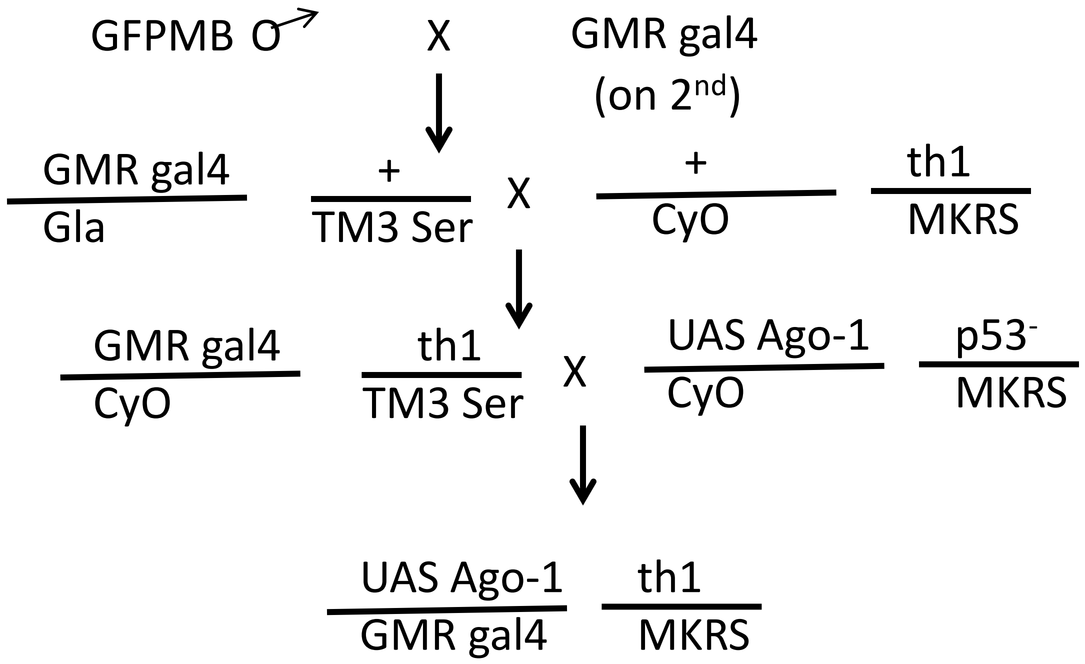

Ago^{72}/Ago^{45} ; GMR-hid/GMR-hid

a. UAS-Ago-1/Gla; p53-/Ser X GMR-hid/GMR-hid

↓

+/Gla; GMR-hid/Ser

b. UAS-Ago-1/Gla; p53-/Ser X GMR-grim/GMR-grim

↓

+/Gla; GMR-grim/Ser

c. UAS-Ago-1/Gla; p53-/Ser X GMR-rpr/GMR-rpr

↓

+/Gla; GMR-rpr/Ser

Ago^{72}/Ago^{45} ; GMR-hid/GMR-hid

a. Ago^{72}/GFP CyO X UAS Ago-1/CyO; p53/MKRS

↓

Ago^{72}/CyO ; +/MKRS X +/Gla; GMR-hid/Ser

↓

Ago^{72}/Gla ; GMR-hid/CyO

b. Ago^{45}/GFP Gla X UAS Ago-1/CyO; p53/MKRS

↓

Ago^{45}/CyO ; +/MKRS X +/Gla; GMR-hid/Ser

↓

Ago^{45}/Gla ; GMR-hid/MKRS

c. Ago^{72}/Gla ; GMR-hid/CyO X Ago^{45}/Gla ; GMR-hid/MKRS

↓

Ago^{72}/Ago^{45} ; GMR-hid/GMR-hid

Ago^{72}/Ago^{45} ; GMR-grim/GMR-grim

a. Ago^{72}/GFP CyO X UAS Ago-1/CyO; p53/MKRS

↓

Ago^{72}/CyO ; +/MKRS X +/Gla; GMR-grim/Ser

↓

Ago^{72}/Gla ; GMR-grim/CyO

b. Ago^{45}/GFP Gla X UAS Ago-1/CyO; p53/MKRS

↓

Ago^{45}/CyO ; +/MKRS X +/Gla; GMR-grim/Ser

↓

Ago^{45}/Gla ; GMR-grim/MKRS

C. Ago^{72}/Gla ; GMR-grim/CyO X Ago^{45}/Gla ; GMR-grim/MKRS

↓

Ago^{72}/Ago^{45} ; GMR-grim/GMR-grim

Ago^{72}/Ago^{45} ; GMR-rpr/GMR-rpr

a. Ago^{72}/GFP CyO X UAS Ago-1/CyO; p53/MKRS

↓

Ago^{72}/CyO ; +/MKRS X +/Gla; GMR-rpr/Ser

↓

Ago^{72}/Gla ; GMR-rpr/CyO

b. Ago^{45}/GFP Gla X UAS Ago-1/CyO; p53/MKRS

↓

Ago^{45}/CyO ; +/MKRS X +/Gla; GMR-rpr/Ser

↓

Ago^{45}/Gla ; GMR-rpr/MKRS

c. Ago^{72}/Gla ; GMR-rpr/CyO X Ago^{45}/Gla ; GMR-rpr/MKRS

↓

Ago^{72}/Ago^{45} ; GMR-rpr/GMR-rpr

UAS Ago-1/GMR GAL4; UAS puc/UAS puc

UAS puc/UAS puc X ywMB

↓

+/CyO; UAS puc/MKRS X UAS Ago-1/Gla; p53-/Ser

↓

UAS Ago-1/CyO; UAS puc/Ser X GMR GAL4; Ser/MKRS

↓

UAS Ago-1/GMR GAL4; UAS puc/MKRS

UAS Ago-1/CyO; UAS puc/Ser X UAS Ago-1/GMR GAL4; UAS puc/MKRS

↓

UAS Ago-1/GMR GAL4; UAS puc/UAS puc

UAS Ago-1 /UAS Ago-1 → Can not survive

hep^{1} /+; UAS Ago-1/GMR GAL4

a. ♂ GMR GAL4 on 2nd X ywMB ♀

↓

y ; GMR GAL4 / CyO

b. hep^{1} / FM6 X ywMB

↓

hep^{1} / y ; +/ Gla

c.

y ; GMR GAL4 / CyO X hep^{1} / y ; +/ Gla

↓

hep^{1} ; GMR GAL4/CyO X UAS Ago-1/Gla; p53-/Ser

↓

hep^{1} /+; UAS Ago-1/GMR GAL4

i. P{GawB}c754 , w^{1118} X P{TRiP.HM04006}attP2

↓

P{GawB}c754/{TRiP.HM04006}attP2

ii. UAS Ago-1/CKG X P{GawB}c754

↓

P{GawB}c754 /+; UAS Ago-1/+

And

P{GawB}c754 /+; CKG/+ (Control)
